# Customized reaction route for ruthenium oxide towards stabilized water oxidation in high-performance PEM electrolyzers

**DOI:** 10.1038/s41467-023-36380-9

**Published:** 2023-02-15

**Authors:** Zhaoping Shi, Ji Li, Yibo Wang, Shiwei Liu, Jianbing Zhu, Jiahao Yang, Xian Wang, Jing Ni, Zheng Jiang, Lijuan Zhang, Ying Wang, Changpeng Liu, Wei Xing, Junjie Ge

**Affiliations:** 1 State Key Laboratory of Electroanalytic Chemistry, Jilin Province Key Laboratory of Low Carbon Chemistry Power, Changchun Institute of Applied Chemistry, Chinese Academy of Sciences, Changchun, 130022 China; 2 School of Applied Chemistry and Engineering, University of Science and Technology of China, Hefei, 230026 China; 3 Shanghai Institute of Applied Physics, Chinese Academy of Sciences, Shanghai, 201204 China; 4 University of Chinese Academy of Sciences, Beijing, 100049 China; 5 Shanghai Synchrotron Radiation Facility, Zhangjiang National Lab, Shanghai Advanced Research Institute, Chinese Academy of Science, Shanghai, 201204 China; 6 State Key Laboratory of Rare Earth Resource Utilization, Changchun Institute of Applied Chemistry, Chinese Academy of Sciences, Changchun, 130022 China

**Keywords:** Electrocatalysis, Electrocatalysis

## Abstract

The poor stability of Ru-based acidic oxygen evolution (OER) electrocatalysts has greatly hampered their application in polymer electrolyte membrane electrolyzers (PEMWEs). Traditional understanding of performance degradation centered on influence of bias fails in describing the stability trend, calling for deep dive into the essential origin of inactivation. Here we uncover the decisive role of reaction route (including catalytic mechanism and intermediates binding strength) on operational stability of Ru-based catalysts. Using MRuO_x_ (M = Ce^4+^, Sn^4+^, Ru^4+^, Cr^4+^) solid solution as structure model, we find the reaction route, thereby stability, can be customized by controlling the Ru charge. The screened SnRuO_x_ thus exhibits orders of magnitude lifespan extension. A scalable PEMWE single cell using SnRuO_x_ anode conveys an ever-smallest degradation rate of 53 μV h^−1^ during a 1300 h operation at 1 A cm^−2^.

## Abstract (web summary)

The poor stability of ruthenium-based catalysts has greatly hampered their application in polymer electrolyte membrane water electrolysis. Here, the authors uncover the decisive role of reaction route on catalytic performance, which enables the screening of efficient ruthenium-based water oxidation catalysts.

## Introduction

Water electrolysis using a polymer electrolyte membrane (PEM) is deemed most suitable to propel a carbon-neutral future by converting the intermittent renewable energy resources into high purity H_2_^1–5^, due to its innate advantages over alkaline water electrolyzer in terms of current density and instant current response^6–8^. In practice, however, large-scale deployment of PEM water splitting is greatly constrained by the water oxidation catalysis half-reaction, which provides protons and electrons for hydrogen production^9,10^, with two essential limitations: (i) the high capital cost of scarce and expensive Ir (US$60,670 kg^−1^) catalysts currently used^11,12^; (ii) the high overpotentials and thereby low energy efficiency of Ir in catalyzing the oxygen evolution reaction (OER)^13^. These two limitations in combination make Ir-based catalysts difficult to meet the 2025 goal of the US Department of Energy (DOE) (H_2_ production cost <US $2 kg_H2_^−1^)^14,15^. Given this, Ru has attracted research interests worldwide with lower prices (US$9,523 kg^−1^), higher earth abundance, and higher intrinsic OER activity^16–18^. However, the inferior dissolution resistance of Ru oxides in harsh anodic and acidic conditions remains long-unsolved, making it unrealistic for practical utilization due to the limited lifespan of only dozens of hours^19–22^. Thus, improving the operational stability of Ru-based catalysts is highly desired^23^.

The lack of pertinent descriptors that could successfully capture the essential origin of the Ru inactivation in operation conditions has led to slow progress in stability improvement^24^. Since 1966, the over-oxidation of Ru to volatile RuO_4_ or soluble RuO_5_^2−^ at a high potential (>1.4 V) has been claimed responsible for its poor stability according to the Pourbaix diagram^25–27^. Thereafter, two main strategies, i.e., either introducing electron-donating elements to lower the Ru oxidation state at fixed anodic potential^28–31^ or enhancing the intrinsic activity to reduce the working potential of the catalysts, were adopted to extend operating life^21,32^. Such efforts, however, have led to only limited progress despite decades of study, and these ideas centered on the influence of bias have been challenged by a few observations in recent years. First, despite that some electron-donating elements (Sr, La, Mn, Ce, etc.) can increase the proportion of low-valent Ru species (i.e., Ru^3+^) and effectively suppress the Ru over-oxidation, inferior operating stabilities were still achieved on these catalysts that bind oxygen too weakly^33–36^. Second, it was only recently, with technological advances allowing for precise reaction mechanism probing, that the involvement of lattice oxygen in OER^37^ has been identified as one major cause for the extremely poor stability according to the previous work from Wohlfahrt-Mehrens and Heitbaum, in spite of boosted activity^18,38–41^. These demonstrations highlight the importance of the long-ignored role of the OER routes (including the reaction mechanism and binding strength of intermediates) in determining catalyst durability. However, delineating the path to constructing a well-stabilized catalyst is still highly challenging and obstructed, as it would require the development of a universal descriptor that could embody both the reaction path and binding energies in a sole structural parameter, which is currently unavailable^42,43^.

Herein, we present an intuitive descriptor, i.e., the Ru charge, to describe the overall activity and stability of the catalysts. We reveal that, despite the complexity, catalyst destabilization is in close association with Ru charge and level of difficulty in Ru-O bond breaking, i.e., the balance of formation energy between oxygen vacancy (Δ*G*_VO_) and the Ru vacancy (Δ*G*_VRu_). By varying the chelating elements surrounding the RuO6 motif via controlling the M-O-Ru structure, we succeeded not only in regulating the reaction mechanism, i.e., choosing either or not to involve the lattice oxygen into the reaction (LOM, supplementary Fig. 1a)^44,45^, but also in tailoring the activity and stability under adsorbate evolution reaction (AEM, supplementary Fig. 1b)^46^. Guided by this understanding, we screened out SnRuO_x_ solid solution with Sn-O-Ru local structure that follows the AEM with appropriate Ru-O binding strength. The SnRuO_x_ catalysts exhibit the high intrinsic activity of 2360 A g_Ru_^−1^ at 1.48 V vs. RHE, with TOF representing the highest value reported for the catalysts following the AEM mechanism, i.e., 0.63 s^−1^. Moreover, the catalyst exhibits the best operational stability to date, with only a 26.8 mV increase in overpotential during the 250 h test under 100 mA cm^−2^ at a low catalyst loading of 41.65 μg_cat_ cm^−2^. In a scalable PEM electrolyzer, an ever-smallest degradation rate of 53 μV h^−1^ is demonstrated during a 1300 h durability test at 1 A cm^−2^, which successfully extends the lifespan of Ru-based catalysts by orders of magnitude, thus unambiguously validating our idea to improve the stability. We put forward a feasible idea to construct a stable and highly-efficient Ru-based OER catalyst, which laid the foundation for the application of Ru-based materials in PEMWE.

## Results

### Rational design of the intuitive descriptor

We initiate by recognizing the lack of pertinent physical parameters to describe the overall performance of the Ru-based catalysts, irrespective of the reaction mechanism. Previously, reactivity descriptors, including the *e*_g_ filling^47,48^, *d* band center^28^, *p* band center^11^, and the adsorption energy difference between catalytic sites and dopants proposed by ref. ^49^. were found feasible in describing the activity trend of the catalysts following either the AEM or LOM mechanism. However, a unified scale, which comprises information on activity and stability in combined reaction mechanisms (both AEM and LOM), is lacking, thereby obscuring the advancing direction of the catalysts.

We reasoned that, despite the complexity, the catalytic activity and stability are interrelated and bridged by the Ru-O bonding nature, i.e. bond covalency and level of difficulty in Ru-O bond breaking^50^, as follows. Weakening the Ru-O bond covalency implies the burying of the O 2*p* orbital under the Fermi level as well as the Ru 3*d* front orbital, thereby prohibiting lattice oxygen from participating in OER and the formation of oxygen vacancy (Δ*G*_VO_)^45,51^. In thus formed catalysts dominated by AEM, too low degree of Ru-O hybridization indicates too weak oxygen binding strength, which causes decreased Ru vacancy formation energy (Δ*G*_VRu_) and eases Ru loss^52^, resulting in a simultaneous decrease in activity and stability. Strengthening the Ru-O bonding will firstly lead to a moderate intermediate binding energy as well as an increased Δ*G*_VRu_ under AEM, thereby improving both the activity and stability^53,54^. However, further increasing the bonding strength leads to arise of lattice oxygen redox, where O 2*p* becomes the front orbital and thereby causes lowered Δ*G*_VO_ value^18^. Such a condition, though greatly boosts the intrinsic activity, causes the formation of oxygen defects and easy detachment of Ru-Ox moieties from defects' neck, leading to rapid catalyst degradation^28,37^. To this end, an appropriate performance indicator might be a physical parameter that could precisely describe the local Ru-O bonding nature.

**Fig. 1:**
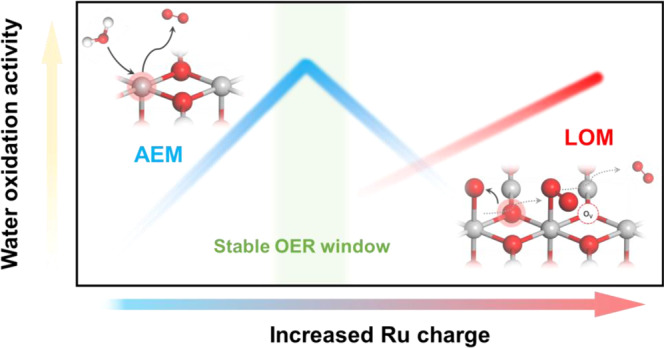
Relation between catalytic performance and Ru charge. Schematic illustration of OER activity and stability of Ru-based catalysts within different reaction routes on the scale of Ru charge.

We postulate that the Ru charge might be able to serve as such an intuitive descriptor, because it could not only perfectly reflect the local Ru-O bonding structure, i.e., the ionic or covalent bonding, but also the bonding strength. Therefore, it is possible to customize both the reaction activity and stability, using Ru charge as a regulator (Fig. 1). We, therefore, begin our study by looking for a suitable model catalyst system to verify this idea, and found that the MRuO_x_ solid solution (M in +4 valence to avoid additional structure influence) with a clear Ru-O-M structure motif might be suitable for such a proposal. Specifically, the Ru charge can be regulated by adjusting the ionic electronegativity of M, where M^4+^ in higher ionic electronegativity than Ru^4+^ leads to reduced electron density on Ru, and vice versa. Consequently, we selected M = Ce^4+^, Sn^4+^, Ru^4+^, and Cr^4+^ with an ionic electronegativity of 1.608, 1.706, 1.848, and 1.861 to carry out our further study^55,56^.

### DFT calculational predictions

**Fig. 2:**
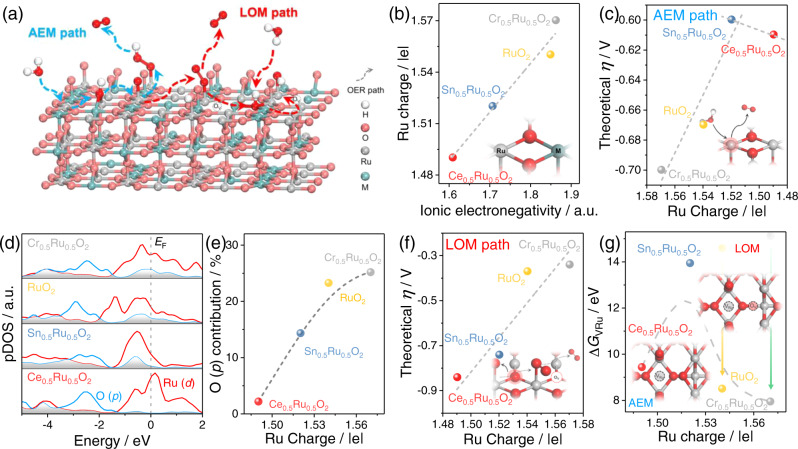
Prediction of the OER performance based on DFT calculation. **a** Schematics of two OER pathway (AEM and LOM) on the surface of M_0.5_Ru_0.5_O_2_ (110). **b** The variation of Ru charge in M_0.5_Ru_0.5_O_2_ with an ionic electronegativity of different M. **c** Theoretical overpotential volcano plot under AEM pathway as a function of the Ru charge. **d** Projected DOS plots of Ru (*d*) and O (*p*) near the Fermi level for M_0.5_Ru_0.5_O_2_. The light gray filling shows the overlap between Ru (*d*) and O (*p*). **e** Variation of O (*p*) contribution near Fermi level with Ru charge. **f** Theoretical overpotential plot under LOM pathway as a function of the Ru charge. **g** The Ru vacancy formation energy (Δ*G*_VRu_) of M_0.5_Ru_0.5_O_2_ is characterized by Ru charge. For RuO_2_ and Cr_0.5_Ru_0.5_O_2_, Δ*G*_VRu_ were calculated without (transparent dots in yellow and green) and with (opaque dots in yellow and green) the consideration of lattice oxygen vacancies. Source data are provided as a Source Data file.

We firstly carried out DFT calculations with the simulation model of M_0.5_Ru_0.5_O_2_ with Ru-O-M motifs constructed (Fig. 2a) based on the stable rutile RuO_2_ (110)^18,57^ (Supplementary Figs. 2–6 and Supplementary Note 1). The charge of Ru in Ru-O-M was first calculated. As the electronegativity of M^4+^ increases from 1.608 (Ce^4+^) to 1.861 (Cr^4+^), the charge of Ru increased from 1.49 (Ce_0.5_Ru_0.5_O_2_) to 1.57 (Cr_0.5_Ru_0.5_O_2_) (Fig. 2b). We then evaluated the dependence of theoretical OER activity on the Ru charge, in both AEM and LOM reaction pathways on Ru sites, as the M sites were calculated to possess extremely poor activities under both reaction pathways (M = Ce, Sn, Cr) (Supplementary Figs. 7–13 and Supplementary Note 2).

The AEM pathway activities of the materials are revealed to follow a volcanic-like trend as a function of Ru charge. The theoretical OER overpotentials of M_0.5_Ru_0.5_O_2_ were calculated first, with the Sn_0.5_Ru_0.5_O_2_ sample exhibiting moderate Ru charge (1.52) representing the lowest theoretical OER overpotential. For the samples with both lower (Ce_0.5_Ru_0.5_O_2_) and higher (RuO_2_ and Cr_0.5_Ru_0.5_O_2_) Ru charge, increases in OER overpotentials are observed (Fig. 2c and Supplementary Figs. 14–17). While this phenomenon implies that an optimum Ru charge that is neither too high nor too low is beneficial for OER, the underlying reason is that Ru charge can be successfully used to reflect the Ru-O bonding interaction. Specifically, we calculated the overlap band center of Ru *d* orbital and O *p* orbital in the partial density of states (pDOS, Supplementary Fig. 18)^58^ and found that the overlap band center (ε-Ru*d*-O*p*) takes the same trending with the change in Ru charge, i.e., increases from −4.19 to −2.95 eV with an increase in Ru charge (1.49–1.57). To this end, the Ru charge can truly be utilized as the intuitive descriptor because it reflects the status of both Ru itself and the Ru-O orbital interaction. Indeed, as the Ru charge increases, the M_0.5_Ru_0.5_O_2_ undergoes alteration in the potential determine step (PDS) from OH*/O* to O*/OOH* (Supplementary Figs. 13, 14), due to strengthening in Ru-O* binding, in line with bond shrinkage from 1.749 to 1.732 Å (Supplementary Fig. 19). Therefore, Ru sites charge can be successfully used to indicate the AEM reaction activity.

We then move to calculate the reactivity of lattice oxygen in different M_0.5_Ru_0.5_O_2_ structures and the feasibility of the LOM route. First, upshifting in O 2*p* band center (Fig. 2d, e and Supplementary Fig. 20) and strengthening in Ru-O bond covalency are observed with increases in Ru charge, as indicated by increases in both ε-O*p*-Ru*d*, O 2*p* band contribution near the *E*_F_ (from 2.2 to 25.2%)^40,59,60^ and downshifted ICOHP between Ru and O (from −1.280 to −1.733 eV, Supplementary Figs. 21, 22). Second, we calculated the formation energies of oxygen vacancy(Δ*G*_VO_) in different samples, to analyze the occurrence possibility of LOM^18^. The significantly downshifted Δ*G*_VO_ from 7.20 eV (Ce_0.5_Ru_0.5_O_2_) and 8.25 eV (Sn_0.5_Ru_0.5_O_2_) to 5.68 eV (RuO_2_) and 5.29 eV (Cr_0.5_Ru_0.5_O_2_) suggests the higher possibility of LOM involvement in RuO_2_ and Cr_0.5_Ru_0.5_O_2_ with high Ru charge (Supplementary Fig. 23). The lower Δ*G*_VO_ of Ce_0.5_Ru_0.5_O_2_ compared to that of Sn_0.5_Ru_0.5_O_2_ may originate from the distortion of the crystal structure^61^ (Supplementary Figs. 24–27 and Supplementary Note 3). Third, we turned to calculate the LOM energy variation for M_0.5_Ru_0.5_O_2_ (Fig. 2f and Supplementary Figs. 28–31). Clearly, the theoretical overpotential of OER under the LOM path gradually decreases from 0.84 V for Ce_0.5_Ru_0.5_O_2_ to 0.34 V for Cr_0.5_Ru_0.5_O_2_. Therefore, the direct O-O coupling on the O *p* band above the *E*_F_ is more thermodynamically favored with a higher Ru charge (i.e., in RuO_2_ and Cr_0.5_Ru_0.5_O_2_). Based on the above calculations, the correlation between a charge of the Ru site and the reaction path (AEM and LOM) as well as reaction energy on M_0.5_Ru_0.5_O_2_ (Fig. 1) is unveiled. While low charge (Ce_0.5_Ru_0.5_O_2_ and Sn_0.5_Ru_0.5_O_2_) at Ru sites leads to the domination of the AEM path, the LOM route is energetically more favored at elevated Ru charge. Moreover, by changing the Ru charge, the OER catalytic activity of catalysts following AEM can also be adjusted due to the regulation of Ru-O interaction in M_0.5_Ru_0.5_O_2_.

We then move to our next step, i.e., probing the applicability of the Ru charge in describing the stability of the catalysts. Two indicators, i.e., the formation energies of Ru (Δ*G*_VRu_) and O (Δ*G*_VO_) vacancies, are used jointly to describe the stability of the catalysts. On one hand, it is expected that increasing the Ru charge results in strengthened Ru-O bonding and thereby increased Δ*G*_VRu_ in perfectly crystallized M_0.5_Ru_0.5_O_2_ samples, with Δ*G*_VRu_ increasing gradually from 9.44 eV in Ce_0.5_Ru_0.5_O_2_ to13.92, 14.60, and 15.13 eV for Sn_0.5_Ru_0.5_O_2_, RuO_2_, and Cr_0.5_Ru_0.5_O_2_, respectively (Fig. 2g). On the other hand, however, increase in Ru charge causes ease formation of O_V_ (Supplementary Fig. 23) and thereby decreases in Δ*G*_VRu_ (Supplementary Fig. 32) due to lower overall Ru-O chelation number. Specifically, with the formation of one neighboring O_V_, the Δ*G*_VRu_ value for RuO_2_ and Cr_0.5_Ru_0.5_O_2_ decreases from 14.60 and 15.13 eV to 8.51 and 7.95 eV, respectively, resulting in poor stability (Fig. 2g). To this end, the Sn_0.5_Ru_0.5_O_2_ near the apex of the volcano curve under the AEM path demonstrates the best stability. The rationale behind this is that the low Ru charge (Ce_0.5_Ru_0.5_O_2_) makes the Ru species prone to dissolution in acid, too high Ru charge leads to the readily generation of O_V_ defects and smaller Δ*G*_VRu_, thus resulting in the overall destabilization. Since metal dissolution may occur during acidic OER, defective structures with Ru and M vacancies were also constructed for calculations (Supplementary Figs. 33–43 and Supplementary Note 4). Although the inclusion of metal center vacancies leads to the trend of crossover between AEM and LOM pathways (Supplementary Fig. 44), in line with recent work from Alexandrov et al.^62,63^, Ru charge is still valid in describing the reaction path and energy as the vacancies influence the Ru charge and OER reaction in the same way. Specifically, the inclusion of vacancies leads to the evolution of the Ru-O bonding feature and thereby Ru charge (Supplementary Fig. 45 and Supplementary Note 5), which thus determines the theoretical activity under different paths. As a result, the theoretical performance of defective M_0.5_Ru_0.5_O_2_ on the scale of Ru charge perfectly matches that of the non-defective structure. To here, it is clear that the Ru charge can be used to describe not only the major reaction path and OER activity, but also the structural stability, due to its successful reflection in both electronic features of Ru and overall Ru-O bonding interactions (Fig. 1).

### Synthesis and characterization

To experimentally validate the DFT prediction, MRuO_x_ oxide solid solution with a M/Ru ratio close to 1 was synthesized according to the previous reports (see Methods for details)^16,35^. Powder X-ray diffraction (XRD) patterns show that all prepared MRuO_x_ samples possess a rutile-type structure without any distinct diffraction peaks corresponding to MO_x_ and RuO_2_ (Supplementary Fig. 46), indicating the formation of MRuO_x_ oxide solid solution, in line with the uniform elements distribution revealed by energy-dispersive X-ray spectroscopy (EDX) (Supplementary Figs. 47–50). Rietveld refinement analysis (Supplementary Fig. 51 and Supplementary Table 1) suggests the random displacement of Ru by M in MRuO_x_ solid solution, giving the formation of Ru-O-M structure motif (Supplementary Fig. 52). The atomic ratios of M and Ru were close to 1, as analyzed by inductively coupled plasma optical emission spectrometer (ICP-OES) and EDX (Supplementary Fig. 53 and Supplementary Table 2).

**Fig. 3:**
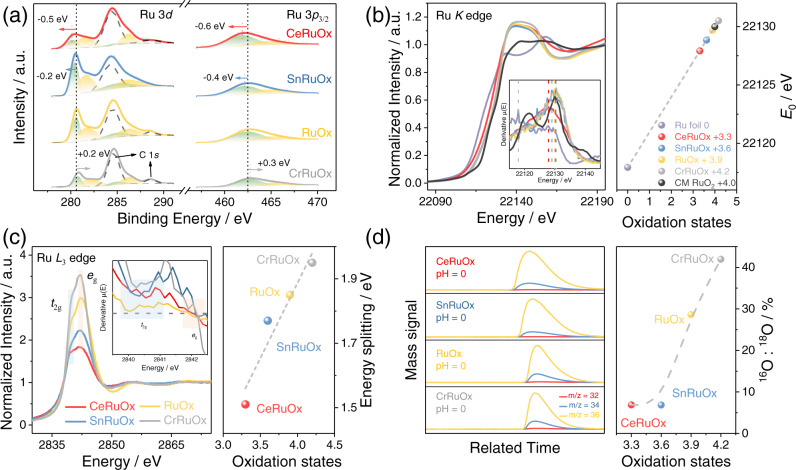
Structure properties of MRuO_x_ oxide solid solution experimentally obtained. **a** High-resolution Ru 3*d* and Ru 3*p*_3/2_ XPS pattern of MRuO_x_, demonstrating the variation of Ru chemical states. **b** Ru *K* edge XANES spectra and corresponding first derivative (insert in the left part of **b**) of MRuO_x_, Ru foil, and oxidized commercial RuO_2_. The right part of **b** show the relation between the Ru *K*-edge absorption energy (*E*_0_) and oxidation states for MRuO_x_ and reference samples. **c** Ru *L*_3_-edge XAS spectra and the corresponding second derivative (insert in the left part of **c**) of MRuO_x_. The right part of **c** illustrated the relation between the energy splitting Δ*E* and Ru oxidation states of MRuO_x_. **d** DEMS signals of ^32^O_2_ (^16^O^16^O), ^34^O_2_ (^16^O^18^O), and ^36^O_2_ (^18^O^18^O) from the reaction products for MRu^16^O_x_ in H^18^O aqueous sulfuric acid electrolyte. The right part of **d** presented the variation of the ^16^O: ^18^O ratio with Ru oxidation states of MRuO_x_. The dashed lines in **c** and **d** are used to guide the eyes. Source data are provided as a Source Data file.

We then move on to probe the local structure and chemical states of Ru and O in MRuO_x_. X-ray photoelectron spectra (XPS) show that both Ru 3*d* and Ru 3*p*_3/2_ (Fig. 3a) signals could be deconvoluted into two doublets, representing Ru^4+^ (fill in green) and the satellite peaks (fill in yellow)^54,64^. Notably, the Ru 3*d* and Ru 3*p* binding energies for CeRuO_x_ (280.1 and 461.9 eV), SnRuO_x_ (280.4 and 462.1 eV), RuO_x_ (280.6 and 462.5 eV), and CrRuO_x_ (280.8 and 462.8 eV) consecutively shifted to more positive values, implying a successive increase in Ru valence states, in line with the DFT calculation results. We further collected the Ru *K* edge spectra through X-ray absorption near-edge spectroscopy (XANES) to quantitatively describe the oxidation state of Ru. As presented in Fig. 3b, taking Ru foil and commercial RuO_2_ as references, the oxidation states of Ru in MRuO_x_ were estimated to be +3.3, +3.6, +3.9, and +4.2 for CeRuO_x_, SnRuO_x_, RuO_x_, and CrRuO_x_, respectively. The variation in Ru valence and its consistency with DFT calculation results verifies the feasibility of tailoring the Ru electron density via controlling the element M in the Ru-O-M motif, making it promising for reaction path and O* adsorption energy regulation.

We next turned to validate experimentally if the OER path of MRuO_x_ truly follows the DFT calculation results as a function of Ru charge, initiated by monitoring the Ru-O bond covalency via Ru XANES spectrum at *L*_3_ edge (Fig. 3c)^65^. As reported previously, the Ru XANES spectrum at the *L*_3_ edge can be fitted by two peaks ascribable to *e*_g_ and *t*_2g_, and the energy splitting Δ*E* (Δ*E* = *E*_*e*g_ - *E*_*t*2g_) is positively correlated with the Ru-O bond covalency. Notably, we find that Δ*E* increases from 1.51 to 1.95 eV (Fig. 3c, right) with the increase in valence state (+3.3 to +4.2), thus testifying to the greatly enhanced Ru-O bond covalency with increased Ru charge, in line with the increase in Ru*d*–O*p* overlap in the calculation (Fig. 2d, e). O 1 *s* XPS spectra (Supplementary Fig. 54 and Supplementary Table 3) further suggest significant increases in O_V_ contents in RuO_x_ (36%) and CrRuO_x_ (41%) compared with that of CeRuO_x_ (25%) and SnRuO_x_ (15%), in good compliance with the increase in bond covalency^11^. The occurrence of LOM is further verified by in situ ^18^O isotope-related DEMS measurements on all MRuO_x_ samples (Fig. 3d, Supplementary Figs. 55–63, and Supplementary Note 6) according to the previous reports^66–68^. By extrapolating the integration of the mass spectra signal (^16^O/^18^O) per second to the OER onset, the content of the LOM path during OER is determined (Supplementary Figs. 55–63e). Specifically, the AEM path is revealed to be dominant on CeRuO_x_ and SnRuO_x_, as the ^16^O/^18^O ratio of O_2_ catalyzed by CeRu^16^O_x_ and SnRu^16^O_x_ in ^18^O-labeled 0.5 M H_2_SO_4_ is 6.88 and 6.85% (Supplementary Figs. 56, 57e), respectively, which is consistent with that of the commercial IrO_2_ (6.56%, Supplementary Fig. 55), attributable to the isotopic abundance of ^16^O in H_2_^18^O. On Ru^18^O_x_ and CrRu^18^O_x_, however, the ^16^O/^18^O ratio in products significantly increased to 28.63 and 41.96%, leading to an increment in the LOM ratio of RuO_x_ and CrRuO_x_ to 12.6 and 36.7%, respectively (Supplementary Figs. 58–59). Besides, the DEMS measurements in ^18^O-labeled 0.1 M PBS (pH = 6) also demonstrate similar results (Supplementary Figs. 60–63). It is noted that the LOM ratio differs significantly in literature as reported by refs. ^67–69^, etc., mainly owing to the difference in structural properties of the catalysts, while our result is in high accordance with that reported by ref. ^38^, due to the similar solid solution structure (Supplementary Note 7). To here, it is clear that the Ru charge is a useful parameter to customize the OER path on MRuO_x_.

### OER catalytic performance in a three-electrode configuration

**Fig. 4:**
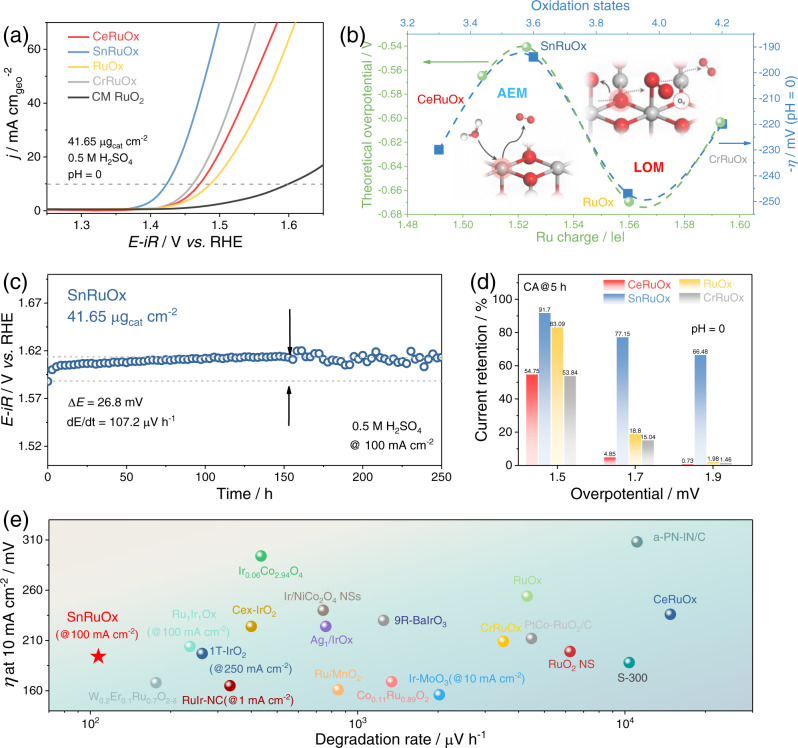
Electrocatalytic performance of MRuO_x_ in three-electrode setup. **a** Geometric area normalized LSV curves of MRuO_x_. **b** The variation of apparent overpotential at 10 mA cm^−2^ with Ru oxidation states. The theoretical overpotential of MRuO_x_ is the weighted value of the theoretical activity of different reaction mechanisms to its proportions (from Supplementary Fig. 56-59). The apparent overpotential experimentally obtained is given as the blue dots scaled with the right Y-axis. The theoretical overpotential is given as the green dots scaled with the left Y-axis. **c** Chronopotentiometry curve of SnRuO_x_ nanocatalyst operated at 100 mA cm^−2^ during the 250 h test. **d** Current retention of MRuO_x_ after chronoamperometry test at various potentials (1.5 V, 1.7 V, and 1.9 V) for 5 h. **e** Comparison of OER performance (*η* at 10 mA cm^−2^ and activity degradation rate) for variously reported electrocatalysts. SnRuO_x_ is presented by a red star. The electrocatalytic performance is collected with a catalyst loading of 41.65 μg_cat_ cm^−2^. Source data are provided as a Source Data file.

The OER performance of the prepared MRuO_x_ was firstly evaluated in a conventional three-electrode setup at a catalyst loading of 41.65 μg_cat_ cm^−2^ (Supplementary Figs. 64, 65 and Supplementary Note 8) with 0.5 M H_2_SO_4_ electrolyte according to the guideline recently set^70,71^. The linear sweep voltammetry (LSV) curves normalized by the geometric area (Fig. 4a), electrochemical active surface area (ECSA) (Supplementary Fig. 66a, b), Ru mass loading on the electrode (Supplementary Fig. 66c) and corresponding Tafel slopes (Supplementary Fig. 66d–f) revealed the variation in OER catalytic activity of MRuO_x_. As summarized in Fig. 4b and Supplementary Fig. 67, the activity trend of MRuO_x_ is in good agreement with DFT prediction. Specifically, on samples dominated by AEM path (CeRuO_x_ and SnRuO_x_), the OER activity is significantly enhanced with an increase in Ru charge (strengthened O* adsorption), accompanied by the variation in reaction determine step as indicated by the difference in Tafel slope (60.2 and 38.2 mV dec^−1^) (Supplementary Fig. 66d and Supplementary Note 9). Further increase in the Ru charge leads to a performance decline in AEM but the occurrence of LOM. Therefore, an activity drop in RuO_x_ and a slight lift in CrRuO_x_ are observed, in line with the DFT calculation. Notably, the SnRuO_x_ customized near the apex of AEM exhibited remarkable OER catalytic activity. Satisfactorily, a small catalytic overpotential (*η*) of 194 mV is needed for SnRuO_x_ to deliver a current density of 10 mA cm^−2^, which is 176 mV lower than that of commercial RuO_2_ (Fig. 4a). Furthermore, the mass activity and turnover frequency (TOF) of SnRuO_x_ at 1.48 V vs. RHE is 36.4 times that of commercial RuO_2_, reaching 2360 A g_Ru_^−1^ and 0.63 s^−1^ (Supplementary Fig. 68). These results unambiguously suggest SnRuO_x_ as one of the most active Ru-based catalysts towards acidic OER (Fig. 4e and Supplementary Table 4).

We then tested the stability of MRuO_x_ during operation to probe into the effectiveness of Ru charge in describing the stability. To start with, chronoamperometry (CA) tests (5 h) suggest SnRuO_x_ as the most stable catalyst, with high current retention of 91.7, 77.15, and 66.48% after operating at 1.5, 1.7, and 1.9 V vs. RHE, respectively (Supplementary Fig. 69). By contrast, the CeRuO_x_, CrRuO_x_, and RuO_x_ only demonstrate current retention of 54.75, 83.09, and 53.84% at 1.5 V vs. RHE, respectively, implying their poor stability. Furthermore, to eliminate the interference of other factors beyond the catalyst, i.e., thin film quality^72,73^, active sites blockage^74^, and back electrode passivation^75,76^, the stability of MRuO_x_ was evaluated by monitoring the dissolution behavior of M and Ru during CA tests. By calculating the Ru stability number (S-number)^77^ during CA tests, the stability of MRuO_x_ is found to be in line with the Δ*G*_VRu_ from the DFT calculations (Supplementary Fig. 70). Given the perfect match in catalytic stability between experiments and theoretical computations, we further run a CP test at 100 mA cm^−2^ for the SnRuO_x_ sample to verify its stability (Fig. 4c). Excitingly, during a 250 h test, the overpotential only increased by 26.8 mV, giving a degradation rate of merely 107.2 μV h^−1^, three orders of magnitude smaller than that of commercial RuO_2_ (Supplementary Fig. 71) and comparable to that of the advanced Ir-based catalysts (Fig. 4e and Supplementary Tables 5, 6)^12,14,18,19,38,52–54,78–90^. Besides, by carrying out CA tests in a weak acidic environment (0.1 M PBS, pH = 6), the contribution of M (M = Ce, Sn, and Cr) dissolution on the performance degradation of MRuO_x_ is eliminated (Supplementary Figs. 72, 73 and Supplementary Note 10), further demonstrating the stability of MRuO_x_ is dependent on the Ru stability. These results corroborate the effectiveness of the Ru charge not only in describing the trend in catalytical activity, but more importantly, in screening out the most durable catalysts ever reported.

### The origin of the high stability

**Fig. 5:**
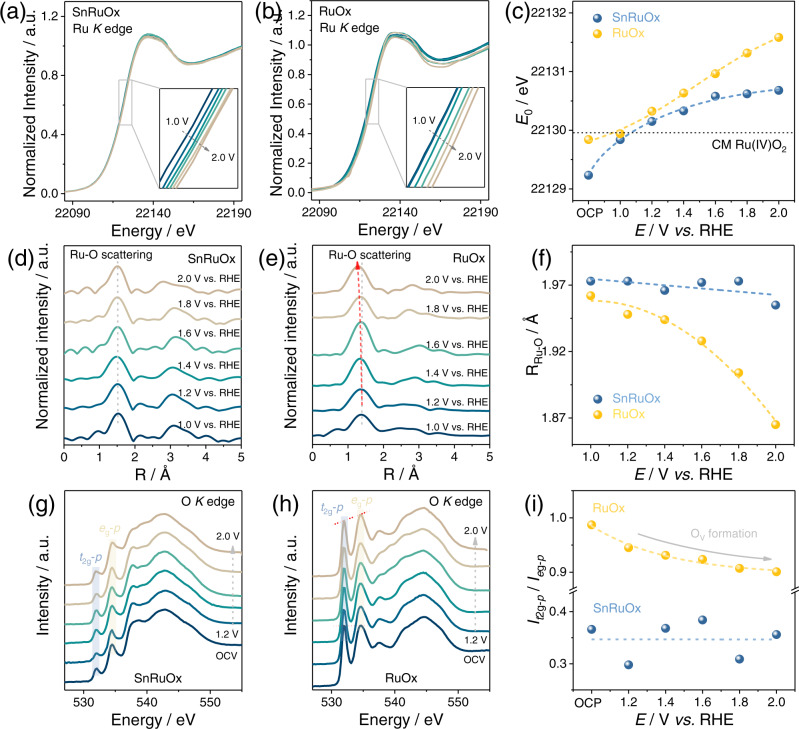
Investigating the stability mechanism of SnRuO_x_ during OER with RuO_x_ as control. **a**, **b** In situ Ru *K* edge XANES spectra of SnRuO_x_ (**a**) and RuO_x_ (**b**) with applied bias rise from 1.0 V to 2.0 V vs. RHE. **c** The variation trend of Ru *K*-edge absorption energy (*E*_0_) of SnRuO_x_ and RuO_x_ under different potentials. **d**, **e** Fourier transforms of *k*^2^-weighted EXAFS signals of SnRuO_x_ (**d**) and RuO_x_ (**e**) with applied bias rise from 1.0 to 2.0 V vs. RHE. **f** Summary of the Ru-O bond length of SnRuO_x_ and RuO_x_ under various potentials according to the quantitative fitting results (Supplementary Table 7). **g**, **h** Quasi in situ O *K* edge XAS TEY spectra of SnRuO_x_ (**g**) and RuO_x_ (**h**) with an applied potential increase from 1.2 to 2.0 V vs. RHE. **i** The variation of *I*_*t*2g-*p*_/*I*_*e*g-*p*_ with applied potential for SnRuO_x_ and RuO_x_. The dashed lines in **c**, **f**, **i** are used to guide the eyes. Source data are provided as a Source Data file.

To further probe into the stabilization mechanism of SnRuO_x_ during OER operation, we adopted in situ XAS and in situ Raman spectroscopy to investigate the dynamic variation in local Ru structure and chemical environment. RuO_x_ was also tested as the counterpart catalyst for comparison. According to the literature, the over-oxidation of Ru originating from the structure damage under high oxidation potential is one of the main culprits leading to severe Ru dissolution^91^. Thus, Ru *K* edge XANES spectra were collected to monitor the change in Ru oxidation states of SnRuO_x_ and RuO_x_ during operation. As presented in Fig. 5a, b and summarized in Fig. 5c, SnRuO_x_ and RuO_x_ show big differences in Ru valence states during operation (Supplementary Fig. 74), especially at higher applied potentials. Specifically, switching on the electrode potential from open circuit potential (OCP) to 1.4 V vs. RHE leads to a rapid rise in Ru valence from +3.6 to +4.2 in SnRuO_x_, followed by a gentle and steady change from +4.2 to +4.4 with potential further lifted to 2.0 V vs. RHE. On the contrary, the Ru oxidation states in RuO_x_ increase at a constant rate from +3.9 to +4.9 with the applied potential ranging from OCP to 2.0 V vs. RHE, showing no sign of stabilization. This result indicates that even at high oxidation potentials, the stable structure of SnRuO_x_ can be maintained under the customized AEM path, whereas RuO_x_ undergoes significant structural evolution, probably ascribable to the involvement of LOM.

We then carried out in situ Raman spectroscopy to clarify the structure evolution during OER. For both SnRuO_x_ and RuO_x_, two major peaks at ~435 and 595 cm^−1^ assignable to *E*_g_ and *A*_1g_ vibration modes were observed (Supplementary Fig. 75)^92,93^. With the potential increases from OCP to 1.8 V vs. RHE, the SnRuO_x_ sample maintains a constant Raman shift, suggesting the consistency in Ru-O bonding structure during OER. However, a *ca*. 9 cm^−1^ positive shift in RuO_x_ is noticed, implying the shrinkage in Ru-O bonding length during OER. To intuitively uncover the local structure evolution of SnRuO_x_ and RuO_x_ during OER, Fourier transforms (FTs) analysis of extended X-ray absorption fine structure (EXAFS) spectra was carried out to provide *R*-space information. A prominent peak of ~1.45 Å (phase uncorrected) corresponds to the first Ru-O coordination shell can be observed (Fig. 5d, e and Supplementary Figs. 76, 77) for both samples. Interestingly, while only small fluctuations in Ru-O bond length (r_Ru-O_) are observed for SnRuO_x_ (from 1.97 to 1.96 Å) with potential increases from 1.0 to 2.0 V vs. RHE, a significant r_Ru-O_ shrinkage from 1.96 to 1.86 Å is observed in RuO_x_ (Fig. 5f), in well accordance with the Raman results. Meanwhile, while quantitative Ru *K* edge fitting results (Supplementary Table 7) show no significant change in Ru-O coordination number (CN) for SnRuO_x_, a significant decrease in CN from 6.4 to 4.7 is evidenced in RuO_x_, ascribable to the generation of O_V_ due to the occurrence of LOM.

We further performed quasi in situ soft-XAS measurements to collect the O *K* edge information, to directly evidence the formation of O_V_ by recording the total electron yield (TEY) intensity before and after operating under different potentials. Two pre-edge peaks at ~532 and 534.5 eV assignable to the unoccupied orbital of O 2*p* hybridized with Ru 4*d t*_2g_ and *e*_g_ orbitals are observed for both SnRuO_x_ and RuO_x_ (Fig. 5g–i)^45^. According to previous reports, the reduction in *t*_2g_-*p*/*e*_g_-*p* peak intensity (*I*_*t*2g-*p*_/*I*_*e*g-*p*_) implies the formation of the O_V_ due to the electron occupation of the lowest energy state (*t*_2g_) of octahedral symmetry^94,95^. For RuO_x_, the ratio of *I*_*t*2g-*p*_/*I*_*e*g-*p*_ decreases monotonously with potential increases from 1.2 to 2.0 V vs. RHE, implying the generation of a high amount of O_V_ during OER. On the contrary, no obvious variation in *I*_*t*2g-*p*_/*I*_*e*g-*p*_ is observed for SnRuO_x_, suggesting that there is no increase in O_V_ content and thus ruling out the occurrence of LOM, which endows it with excellent operational stability. These results are in good agreement with the catalyst design idea as SnRuO_x_ was customized to follow the AEM path and exhibited high stability during OER due to moderate Ru charge.

### Performance of PEMWE devices

**Fig. 6:**
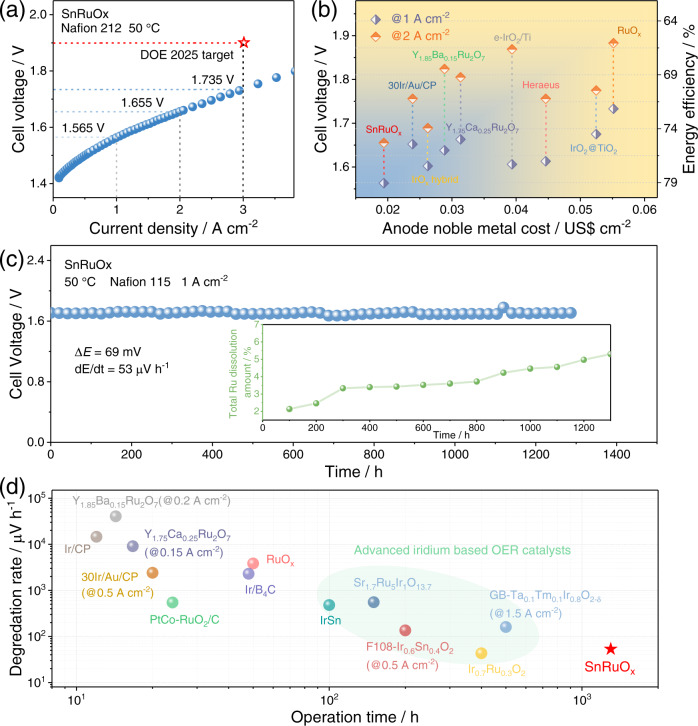
Performance of PEM electrolyzer using SnRuO_x_ as anode electrocatalyst. **a** Polarization curve of the PEM electrolyzer obtained at 50 °C with Nafion 212 membrane. **b** Comparison of the efficiency of previously reported anode electrocatalysts. The X-axis represents the anode noble metal cost (Supplementary Table 8). The left and right Y-axis represent the cell voltage and energy efficiency at 1 and 2 A cm^−2^ respectively. **c** Chronopotentiometry curve of the PEM electrolyzer using SnRuO_x_ catalyst operated at 1 A cm^−2^ at 50 °C with Nafion 115 membrane. Insert in **c** show the total dissolution amount of Ru during the 1300 h test. **d** Comparison of the operational stability (operation time and performance degradation rate) of SnRuO_x_ with reported anode catalysts. Source data are provided as a Source Data file.

Encouraged by the overall high activity and stability of SnRuO_x_ towards OER, we finally assembled a single cell using the catalyst as the anode to evaluate its performance in a real PEMWE device (Supplementary Fig. 78, see Methods for details). The steady-state polarization curve of membrane electrode assemblies (MEAs) operated at 50 °C (Fig. 6a) shows that the performance of PEMWE can be greatly improved with the utilization of SnRuO_x_. Specifically, cell voltages of only 1.565, 1.655, and 1.735 V are needed to reach current densities of 1, 2, and 3 A cm^−2^, respectively, far superior to that obtained with commercial RuO_2_ (1.733 V@1 A cm^−2^ and 1.883 V@2 A cm^−2^) (Supplementary Fig. 79) and also well surpassing the DOE 2025 target (3 A cm^−2^@1.9 V)^96^. Taking noble metal cost and cell performance into account, the overall advantages of SnRuO_x_ are self-evident (Fig. 6b). With anode noble metal cost of US$ 0.0194 cm^−2^, high energy efficiencies at 78.7 and 74.3% were achieved at 1 and 2 A cm^−2^, respectively, corresponding to energy consumptions at 41.9 and 44.4 kWh kg^−1^H_2_, outperforming the prior reported most cost-efficient OER catalysts (Fig. 6b, Supplementary Table 8, and Supplementary Note 11)^20,97–102^. According to the calculation from US DOE, only US$ 0.838 is required for this PEMWE device to produce 1 kg H_2_, which is much lower than the DOE target (<US$2 kg_H2_^−1^), further proving the efficiency of the SnRuO_x_ at the anode.

The long-term operational stability is even more exciting, as shown in Fig. 6c, the SnRuO_x_-based cell demonstrates well-maintained PEMWE performance after running under 1 A cm^−2^ for 1300 h, with apparent degradation rate only at 53 μV h^−1^. Such stability not only represents a record for Ru-based catalysts, but even superior to that of the advanced Ir-based OER catalysts recently reported (Fig. 6d and Supplementary Table 9)^14,20,38,52,97,99,103–107^. To more accurately determine the lifetime of SnRuO_x_ in PEMWE, a modified MEA setup without metallic parts in the anode and cathode water cycle was employed according to the previous reports (Supplementary Fig. 80–84, Supplementary Table 10, and Supplementary Note 12)^108^. With all the Ru dissolved in the anode/cathode water and membrane monitored during 10 h operation at 1 A cm^−2^, the S-number of SnRuO_x_ is calculated to be 1.04 × 10^6^ (Supplementary Note 13)^77^. These results reveal the inherently robust nature of SnRuO_x_ as an OER catalyst with highlighted practical application prospects.

## Discussion

In summary, we presented a practical and feasible idea, namely customizing the reaction route, to design a highly active and stable Ru-based OER catalyst that can afford long-term operation in PEMWE devices. Based on the widely recognized OER mechanism, we found that Ru charge can be used as a scale for the reaction path of the catalyst, only those located close to the apex of the volcano plot in AEM can exhibit optimal stability. Subsequently, we verified the feasibility of this idea in an MRuO_x_ model catalyst. By screening M^4+^ with different ionic electronegativity, the Ru charge can be effectively regulated attributed to the charge redistribution effect in a Ru-O-M structure motif. As a result, the SnRuO_x_ customized exhibited unparallel OER catalytic activity and stability. A PEMWE device assembled with SnRuO_x_ as an OER catalyst can afford a cell voltage of only 1.565 V at 1 A cm^−2^ as well as long-term stability over 1300 h with a degradation rate of only 53 μV h^−1^. This work provides a rational catalyst design concept at the level of reaction path customization, and provides a feasible solution for the practical application of Ir-free Ru-based catalysts in commercial PEMWE systems.

## Methods

### Materials

All chemicals were obtained from commercial suppliers at analytical grade and used as received without further purification. The commercial RuO_2_ and Water−^18^O (H_2_^18^O) were purchased from Aladdin. Carbon paper was obtained from Engineered Fibers Technology, LLC. Commercial Pt/C (40 wt% Pt) catalyst was purchased from Johnson Matthey Company. Titanium felt were purchased from Bekaert New Materials. 5 wt% Nafion® ionomer, Nafion 115 membrane, and Nafion 212 membrane were purchased from DuPont Co. The L shape gold electrode with an area of 0.07065 cm^2^ is obtained from Tianjin Aida Hengsheng Technology Development Co., Ltd.

### Preparation of CeRuO_x_ solid solution

The CeRuO_x_ solid solution was fabricated via a pyrolysis-thermal annealing strategy. Typically, glucose, RuCl_3_, and CeCl_3_·7H_2_O were mixed and dried to obtain a xerogel with Ru and Ce uniformly distributed. Then the precursor was heated in air at 350 °C for 5 h with a heating rate of 5 °C min^−1^. After cooling to room temperature, the collected samples were carefully ground and ultrasonically washed in 1 M HCl for at least 6 h to remove acid-labile components.

### Preparation of SnRuO_x_ solid solution

The SnRuO_x_ solid solution was prepared following the procedure of ion exchange and thermal annealing. Firstly, an Sn-based metal-organic framework (MOF) was fabricated according to the previous reports. The ethanol solution of SnCl_4_ and HMTA (Hexamethylenetetramine) is uniformly mixed and stirred at room temperature for 1 h, washed, and dried to obtain Sn-HMTA. Then Sn-HMTA were dispersed in the THF (Tetrahydrofuran) solution of RuCl_3_. After stirring for 24 h, the mixture is centrifuged and washed with THF to obtain an Sn/Ru-HMTA precursor. The dried products are conducted to heat treatment in air at 350 °C for 5 h with a heating rate of 5 °C min^−1^. After cooling to room temperature, the collected samples were carefully ground and ultrasonically washed in 1 M HCl for at least 6 h to remove acid-labile components.

### Preparation of RuO_x_

The preparation process of RuO_x_ is similar to that of SnRuO_x_. The THF solution of RuCl_3_ and HMTA was ultrasonically mixed and stirred at room temperature for 24 h, followed by centrifugation and washing. After drying in the oven, the Ru-HMTA precursor were annealed at 350 °C for 5 h with a heating rate of 5 °C min^−1^ in air. After cooling to room temperature, the collected samples were carefully ground and ultrasonically washed in 1 M HCl for at least 6 h to remove acid-labile components.

### Preparation of CrRuO_x_ solid solution

The CrRuO_x_ solid solution were prepared according to the previous reports. The Cr, Ru containing precursors were obtained by mixing RuCl_3_ and MIL-101(Cr) in THF and stirring for 24 h. The result RuCl_3_-MIL-101(Cr) were then heated to 450 °C and held for 5 h in air with a heating rate of 5 °C min^−1^. After cooling to room temperature, the collected samples were carefully ground and ultrasonically washed in 1 M HCl for at least 6 h to remove acid-labile components.

### Structural characterizations

Powder X-ray diffraction (XRD) patterns were collected using a Rigaku Smartlab diffractometer with Cu Kα radiation (λ = 1.5418 Å) in the scan range of 10° to 90° at a step of 0.01°. Scanning electron microscopy (SEM) measurements were performed on an XL 30 ESEMFEG field emission scanning electron microscopy. Transmission electron microscopy (TEM), high-resolution transmission electron microscopy (HRTEM), and elemental mapping analysis were performed on a Philips TECNAI G2 electron microscope operating at 200 kV. High-annular dark-field scanning transmission electron microscopy (HAADF-STEM) images were performed on a Titan 80-300 scanning/transmission electron microscopy operated at 300 kV. The surface electronic states were determined by X-ray photoelectron spectroscopy (XPS) using a Thermo Fisher Scientific ESCALAB 250Xi unit with Al-Kα (1486.6 eV) as the X-ray source. Inductively coupled plasma optical emission spectroscopy (ICP-OES; X Series 2, Thermo Scientific USA) was used to determine the ratio of M/Ru in the as-prepared samples and the dissolution of M and Ru after the stability tests.

### XAFS data collection and analysis

The ex situ and in situ Ru *K*-edge XAFS measurements were performed at BL14W1 station in Shanghai Synchrotron Radiation Facility (SSRF, 3.5 GeV, 220 mA maximum, Si(111) double crystals). The Ru *K*-edge spectra of the samples were collected in transmission mode using a Lytle detector. The XAFS raw data were background-subtracted and normalized by the ATHENA program. Least-squares curve-fitting analysis of the EXAFS χ(k) data (including different coordination shells, in the *R*-space (1.0−3.0 Å)) and Fourier transforms (in the *k*-space 3.1−11.0 Å^−1^) were carried out using the ARTEMIS program. The theoretical scattering amplitudes, phase shifts, and the photoelectron mean free path for all paths were calculated by using a Hanning window (d*k* = 1.0 Å^−1^) in the IFEFFIT package. The data were fitted in *R*-space with theoretical models constructed on the basis of the crystal structure derived from XRD. The ex situ Ru *L*_3_ edge measurements were carried out at the soft X-ray microcharacterizaiton beamline (SXRMB) in Canadian Light Source (CLS). The O *K* edge XAFS was measured at the BL08U1A beamline, SSRF.

### Electrochemical measurements in a three-electrode cell

The electrochemical performance of the as-prepared catalysts were measured in a standard three-electrode cell on PARSTAT MC (AMETEK®). The electrocatalysts (1 mg) were dispersed in a mixture of 0.890 mL ethanol, 100 μL ethylene glycol, and 10 μL Nafion® solution (5 wt%). The ethylene glycol is introduced to improve the quality of the catalyst thin film and suppress the coffee-ring effect according to the previous work from ref. ^6^. After ultrasonication for 1 h, the desired amount of homogeneous ink was dropped on a polished L shape gold electrode (area 0.07065 cm^−2^) and dried in an oven (60 °C) for 30 min and then serve as a working electrode. After loading optimization, the mass loading of the sample catalyst and controls are determined to be 41.65 μg_cat_ cm^−2^. The Ru loading on the electrode is estimated to be 13.44, 15.21, 25.50, and 19.02 μg cm^−2^ for CeRuO_x_, SnRuO_x_, RuO_x_, and CrRuO_x_, respectively. The electrochemical testing is carried out in a conventional three-electrode set-up with a carbon rod and a mercurous sulfate electrode (Hg/Hg_2_SO_4_) used as the counter electrode and reference electrode respectively. O_2_-saturated 0.5 M H_2_SO_4_ was used as an electrolyte to mimic the environment of the anode in PEMWE. When conducting LSV, CA, and CP tests, the electrolyte is convected by external magnetic stirring with a speed of 300 rpm, which is sufficient to remove the visible oxygen bubble formed on the surface of the Au electrode. When collecting the CV for ECSA calculation, no magnetic stirring is applied. To conduct the CP at 100 mA cm^−2^, the working electrode is prepared by air spraying the homogeneous catalyst ink on a piece of carbon paper (area 1 × 1 cm^−2^) with optimized catalyst loading (41.65 μg_cat_ cm^−2^). All the electrochemical testing in a three-electrode set-up is conducted at 30 °C. To investigate the dissolution of M during OER, O_2_-saturated 0.1 M PBS (pH = 6) was also used as an electrolyte. For the measurement with *iR*-correction, *R* was referred to as the ohmic resistance arising from the electrolyte/contact resistance of the setup. The *iR* compensation was performed manually after the measurement for 100% of the *R*.

### Determine the ECSA of MRuO_x_

The ECSAs were obtained from the electrochemical double-layer capacitance of the catalytic surface. The electrical double-layer capacitor (C_DL_) were measured from double-layer charging curves using cyclic voltammograms in a non-Faradaic region with a scan rate from 5 to 25 mV s^−1^. The CDL was measured by the following equation:

1 $${{{{{\rm{j}}}}}}={{{{{{\rm{\nu }}}}}}{{{{{\rm{C}}}}}}}_{{{{{{\rm{DL}}}}}}}$$

where j is the no-Faradaic capacitive current obtained from the CV curve, ν is the scan rate. The slope of j-ν curve is regarded as C_DL_. The ECSA is calculated from the double-layer capacitance according to:

2 $${{{{{\rm{ECSA}}}}}}={{{{{{\rm{C}}}}}}}_{{{{{{\rm{DL}}}}}}}/{{{{{{\rm{C}}}}}}}_{{{{{{\rm{S}}}}}}}$$

Where C_S_ is the specific capacitance of the sample (set as 0.035 mF cm^−2^ according to the previous reports).

### Mass activity calculation

The mass activity (j_mass_) of MRuO_x_ were determined using the following equation:

3 $${j}_{{{mass}}}=\frac{{i}_{{{geo}}}}{{m}_{{{cat}}}\times {{Ru}}\,{{wt}}\%}$$

where *i*_geo_ is the geometric current (A) obtained from LSV, m_cat_ is the loading of catalysts on the electrode and Ru wt% is the mass ratio of Ru in MRuO_x_, which is calculated from the results of ICP-OES.

### Calculation of the TOF

The TOF of MRuO_x_ and commercial RuO_2_ were calculated based on the following equation:

4 $${{{{{\rm{TOF}}}}}}=\frac{{n}_{{{{{{{\mathrm{O}}}}}}}_{2}}}{{n}_{{{Ru}}}}$$

where ${n}_{{{{{{{\mathrm{O}}}}}}}_{2}}$ is the number of oxygen per second, ${n}_{{{{{{{\mathrm{Ru}}}}}}}}$ is the number of Ru participating in the OER. Here in this work, all Ru on the electrode are assumed to be the active center during OER. Thus, the ${n}_{{Ru}}$ can be calculated as:

5 $${n}_{Ru}=\frac{{m}_{cat}\times Ru\,wt\%}{{M}_{Ru}}$$

in which ${M}_{{Ru}}$ is the molar mass of Ru (101.07 g mol^−1^). ${m}_{{cat}}$ and ${Ru\; wt}\%$ are same as those in Eq. 3.

The ${n}_{{O}_{2}}$ in Eq. (4) were calculated from the LSV curve by the following equation:

6 $${n}_{{O}_{2}}=\frac{{i}_{geo}}{z\ast F}$$

where ${i}_{{geo}}$ is the geometric current (A), z is the number of electrons required per oxygen molecule, F is the Faraday constant (96,484 C mol^−1^).

### In situ DEMS with isotope labeling

In situ DEMS experiments was performed on an in situ differential electrochemical mass spectrometer provided by Linglu Instruments (Shanghai) Co. Ltd with a delay time of 1 s. A porous PTFE membrane is employed for gas-liquid separation. A typical test was carried out in a three-electrode cell with ^18^O-labeled 0.5 M H_2_SO_4_ or ^18^O-labeled 0.1 M PBS as electrolyte and MRu^16^O_x_ as a catalyst. Before the DEMS measurement, cyclic voltammetry was performed in the range of 0.6 − 1.2 V at 50 mV s^−1^ to further purge the adsorbed ^16^O species on the electrode. After these processes, the in situ DEMS measurement was carried out with five consecutive LSV test on MRuO_x_. To exclude the influence of the abundance of ^16^O in H_2_^18^O, control experiments on commercial IrO_2_ were carried out. The total signal intensity of O_2_ is calculated to determine the OER onset potential.

### Electrochemical measurements in PEM electrolyzer

To construct the membrane electrode assembly (MEA), SnRuO_x_ or commercial RuO_2_ were used as the anode catalysts, and commercial Pt/C (40 wt%) was used as the cathode catalyst. To prepare the anode and cathode ink, catalysts were dispersed to a mixture of isopropanol and distilled water with a ratio of 1:1. Then, Nafion® solution (5 wt%) was added in to obtain an ionomer amount of 30 wt% for an anode and 40 wt% for a cathode. After ultrasonicated for at least 1 h in a low-temperature water bath, a uniform catalyst ink can be obtained.

To prepare the MEA with Nafion 212 membrane as an electrolyte, the anode and cathode catalysts were directly air sprayed on the two sides of the as-received Nafion 212 membrane in the ultrasonic spray coating system. The anode and cathode catalysts loading are controlled to be 4 mg_cat_ cm^−2^ (1.46 mg_Ru_ cm^−2^) and 1 mg cm^−2^ (0.4 mg_Pt_ cm^−2^) after loading optimization. To prepare the MEA with Nafion 115 membrane as an electrolyte, the anode and cathode catalysts are first sprayed onto sheets of polytetrafluoroethylene (PTFE). After that, the PTFE-supported Pt/C, Nafion 115, and PTFE-supported anode catalysts are hot pressed together at 135 °C for 3 min under a pressure of 2 T. After cooling, the PTFE on the surface were carefully peeled off to get the catalyst-coated membrane (CCM). The anode and cathode catalysts loading are controlled to be 2 mg_Ru_ cm^−2^ and 1 mg cm^−2^ after loading optimization. The CCM prepared was preserved in distilled water for further measurements.

To construct the PEM electrolyzers for performance evaluation, the titanium felt with a thickness of 1 mm were used as the porous transport layers (PTL) in both the anode and cathode. The assembly pressure of the fixture is set to 5 N m. The active area of the electrode is measured to be 4 cm^2^.

The PEM electrolyzers were operated at 80 °C with distilled water as reactant under a flow rate of 40 mL min^−1^. The polarization curves were collected from 0.1 to 4.4 A cm^−2^. The stability of the PEM electrolyzers were evaluated by measuring chronopotentiometry at 1 A cm^−2^ for 1300 h. The anode dissolution amount were monitored every 100 h by ICP-OES.

### DFT calculation

The geometries and energies were performed by density functional theory (DFT) with Vienna ab initio simulation package (VASP)^109–116^. The interactions between ion cores and valence electrons were described by the projector augmented wave (PAW) method. The generalized gradient approximation with the Perdew–Burke–Ernzerhof was adopted as the exchange-correlation functional. The wave functions at each k-point were expanded with a plane wave basis set. The kinetic cutoff energy was set to 400 eV. The integration of the Brillouin zone is conducted using a 1 × 1 × 1 Monkhorst-Pack grid. The geometries were optimized using a force-based conjugate-gradient method until the energy was converged to 1.0 × 10^−4^ eV/atom and the force to 0.05 eV/Å. Spin polarization was considered in our current study and the U parameter used for M atoms was 4.0 eV.

The structure of M_0.5_Ru_0.5_O_2_ were based on the results from XRD refinement. M_0.5_Ru_0.5_O_2_ (110) was constructed by cutting the bulk M_0.5_Ru_0.5_O_2_ alone in 110 direction and four layers were selected. For defective structures, Ru and M vacancies are considered (see Supplementary Note 4). During the optimization, the up-layer atoms were allowed to relax and the atoms in the last layer were fixed to keep the bulk structure. A vacuum layer of 15 Å was used along the c direction normal to the surface to avoid periodic interactions.

To illustrate the activity of AEM (adsorbate evolution mechanism) and LOM (a lattice-oxygen-mediate mechanism), the free energy diagrams was estimated as follows:

$$\Delta {{{{{\rm{G}}}}}}=\Delta {{{{{\rm{E}}}}}}+\Delta {{{{{\rm{ZPE}}}}}}-{{{{{\rm{T}}}}}}\Delta {{{{{\rm{S}}}}}}$$

where *ΔE* is the total energy change based on the DFT calculations, *ZPE* and *S* is the zero-point energy and entropy, respectively, *T* is the temperature (here, 298.15 K is selected). The free energy of (H^+^ + e^-^) at standard conditions is assumed as the energy of 1/2 H_2_.

## Supplementary information

Supplementary Information

## Data Availability

The data that support the findings of this study are available within the article and its Supplementary Information files. All other relevant data supporting the findings of this study are available from the corresponding authors upon request. Source data are provided with this paper.

## Source data

Source Data

## References

[1] Lagadec MF, Grimaud A (2020). Water electrolysers with closed and open electrochemical systems. Nat. Mater..

[2] Seitz LC (2016). A highly active and stable IrO_x_/SrIrO_3_ catalyst for the oxygen evolution reaction. Science.

[3] Li A (2022). Enhancing the stability of cobalt spinel oxide towards sustainable oxygen evolution in acid. Nat. Catal..

[4] Lin C (2021). In-situ reconstructed Ru atom array on α-MnO_2_ with enhanced performance for acidic water oxidation. Nat. Catal..

[5] Wei Ju-Cai SLWX (2022). Simultaneous hydrogen and (NH_4_)_2_SO_4_ productions from desulfurization wastewater electrolysis using MEA electrolyser. J. Electrochem..

[6] Zhao F (2021). Increasing iridium oxide activity for the oxygen evolution reaction with hafnium modification. J. Am. Chem. Soc..

[7] Carmo M (2013). A comprehensive review on PEM water electrolysis. Int. J. Hydrog. Energy.

[8] Xie Wen-Fu SM-F (2022). Alkaline water electrolysis for efficient hydrogen production. J. Electrochem..

[9] Nong HN (2020). Key role of chemistry versus bias in electrocatalytic oxygen evolution. Nature.

[10] Nong HN (2018). A unique oxygen ligand environment facilitates water oxidation in hole-doped IrNiOx core–shell electrocatalysts. Nat. Catal..

[11] Kuznetsov DA (2020). Tailoring lattice oxygen binding in ruthenium pyrochlores to enhance oxygen evolution activity. J. Am. Chem. Soc..

[12] Li N (2021). Identification of the active-layer structures for acidic oxygen evolution from 9R-BaIrO_3_ electrocatalyst with enhanced iridium mass activity. J. Am. Chem. Soc..

[13] Wang H (2021). Significantly enhanced overall water splitting performance by partial oxidation of Ir through Au modification in core-shell alloy structure. J. Am. Chem. Soc..

[14] Hao S (2021). Torsion strained iridium oxide for efficient acidic water oxidation in proton exchange membrane electrolyzers. Nat. Nanotechnol..

[15] Guido, B. & Dinh, H. N. *HydroGEN: Low-temperature Electrolysis (LTE) and LTE/Hybrid Supernode.* Report No*.* NREL/PR-5900-76549 (National Renewable Energy Lab, 2020)

[16] Lin Y (2019). Chromium-ruthenium oxide solid solution electrocatalyst for highly efficient oxygen evolution reaction in acidic media. Nat. Commun..

[17] Cao L (2019). Dynamic oxygen adsorption on single-atomic Ruthenium catalyst with high performance for acidic oxygen evolution reaction. Nat. Commun..

[18] Hao S (2020). Dopants fixation of Ruthenium for boosting acidic oxygen evolution stability and activity. Nat. Commun..

[19] Zhao ZL (2020). Boosting oxygen evolution reaction using defect-rich ultra-thin ruthenium oxide nanosheets in acidic media. Energy Environ. Sci..

[20] Feng Q (2020). Influence of surface oxygen vacancies and ruthenium valence state on the catalysis of pyrochlore oxides. ACS Appl. Mater. Interfaces.

[21] Hodnik N (2015). New insights into corrosion of ruthenium and ruthenium oxide nanoparticles in acidic media. J. Phys. Chem. C..

[22] Cherevko S (2014). Dissolution of noble metals during oxygen evolution in acidic media. ChemCatChem.

[23] Roy C (2018). Trends in activity and dissolution on RuO_2_ under oxygen evolution conditions: particles versus well-defined extended surfaces. ACS Energy Lett..

[24] Kasian O (2016). On the origin of the improved ruthenium stability in RuO_2_–IrO_2_ mixed oxides. J. Electrochem. Soc..

[25] Spori C (2017). The stability challenges of oxygen evolving catalysts: towards a common fundamental understanding and mitigation of catalyst degradation. Angew. Chem. Int. Ed..

[26] Wang Z (2020). Predicting aqueous stability of solid with computed Pourbaix diagram using SCAN functional. NPJ Comput. Mater..

[27] Cherevko S (2016). Oxygen and hydrogen evolution reactions on Ru, RuO_2_, Ir, and IrO_2_ thin film electrodes in acidic and alkaline electrolytes: a comparative study on activity and stability. Catal. Today.

[28] Yao Y (2019). Engineering the electronic structure of single atom Ru sites via compressive strain boosts acidic water oxidation electrocatalysis. Nat. Catal..

[29] Chen S (2019). Mn-doped RuO_2_ nanocrystals as highly active electrocatalysts for enhanced oxygen evolution in acidic media. ACS Catal..

[30] Li Z (2020). Mg-Doping improves the performance of Ru-based electrocatalysts for the acidic oxygen evolution reaction. Chem. Commun..

[31] Wang, J. et al. Exceptionally active and stable RuO_2_ with interstitial carbon for water oxidation in acid. *Chem*10.1016/j.chempr.2022.02.003 (2022).

[32] Laha S (2019). Ruthenium oxide nanosheets for enhanced oxygen evolution catalysis in acidic medium. Adv. Energy Mater..

[33] Kim B-J (2017). Unraveling thermodynamics, stability, and oxygen evolution activity of strontium ruthenium perovskite oxide. ACS Catal..

[34] Arčon I (2007). X-ray absorption spectroscopy analysis of Ru in La_2_RuO_5_. X-Ray Spectrom..

[35] He J (2021). Tuning electron correlations of RuO_2_ by co-doping of Mo and Ce for boosting electrocatalytic water oxidation in acidic media. Appl. Catal. B.

[36] Li Q (2020). Identifying the activation mechanism and boosting electrocatalytic activity of layered perovskite ruthenate. Small.

[37] Wohlfahrt-Mehrens M, Heitbaum J (1987). Oxygen evolution on Ru and RuO_2_ electrodes studied using isotope labelling and on-line mass spectrometry. J. Electroanal. Chem..

[38] Wen Y (2021). Stabilizing highly active Ru sites by suppressing lattice oxygen participation in acidic water oxidation. J. Am. Chem. Soc..

[39] Grimaud A (2016). Activation of surface oxygen sites on an iridium-based model catalyst for the oxygen evolution reaction. Nat. Energy.

[40] Grimaud A (2017). Activating lattice oxygen redox reactions in metal oxides to catalyse oxygen evolution. Nat. Chem..

[41] Huang Z-F (2019). Chemical and structural origin of lattice oxygen oxidation in Co–Zn oxyhydroxide oxygen evolution electrocatalysts. Nat. Energy.

[42] Song J (2020). A review on fundamentals for designing oxygen evolution electrocatalysts. Chem. Soc. Rev..

[43] Shi Z (2020). Fundamental understanding of the acidic oxygen evolution reaction: mechanism study and state-of-the-art catalysts. Nanoscale.

[44] Pan Y (2020). Direct evidence of boosted oxygen evolution over perovskite by enhanced lattice oxygen participation. Nat. Commun..

[45] Miao X (2019). Quadruple perovskite ruthenate as a highly efficient catalyst for acidic water oxidation. Nat. Commun..

[46] Man IC (2011). Universality in oxygen evolution electrocatalysis on oxide surfaces. ChemCatChem.

[47] Hwang J (2017). Perovskites in catalysis and electrocatalysis. Science.

[48] Shen G (2020). Regulating the spin state of Fe(III) by atomically anchoring on ultrathin titanium dioxide for efficient oxygen evolution electrocatalysis. Angew. Chem. Int. Ed..

[49] Buvat G (2020). OER performances of cationic substituted (100)-oriented IrO2 thin films: a joint experimental and theoretical study. ACS Appl. Energy Mater..

[50] Yagi S (2015). Covalency-reinforced oxygen evolution reaction catalyst. Nat. Commun..

[51] Wang J (2022). Single-site Pt-doped RuO_2_ hollow nanospheres with interstitial C for high-performance acidic overall water splitting. Sci. Adv..

[52] Jin H (2021). Safeguarding RuO_2_ phase against lattice oxygen oxidation during acidic water electrooxidation. Energy Environ. Sci..

[53] Tian Y (2019). A Co-doped nanorod-like RuO_2_ electrocatalyst with abundant oxygen vacancies for acidic water oxidation. iScience.

[54] Su J (2018). Assembling ultrasmall copper-doped ruthenium oxide nanocrystals into hollow porous polyhedra: highly robust electrocatalysts for oxygen evolution in acidic media. Adv. Mater..

[55] Li K, Xue D (2006). Estimation of electronegativity values of elements in different valence states. J. Phys. Chem. A.

[56] Shannon RD (1976). Revised effective ionic-radii and systematic studies of interatomic distances in halides and chalogenids. Acta Crystallogr. A.

[57] Rao RR (2017). Towards identifying the active sites on RuO_2_(110) in catalyzing oxygen evolution. Energy Environ. Sci..

[58] Shi Z (2021). Confined Ir single sites with triggered lattice oxygen redox: toward boosted and sustained water oxidation catalysis. Joule.

[59] Mefford JT (2016). Water electrolysis on La_1-x_Sr_x_CoO_3-d_ perovskite electrocatalysts. Nat. Commun..

[60] Kim J (2017). High-performance pyrochlore-type yttrium ruthenate electrocatalyst for oxygen evolution reaction in acidic media. J. Am. Chem. Soc..

[61] Lou Y (2014). Ultralow-temperature CO oxidation on an In_2_O_3_–Co_3_O_4_ catalyst: a strategy to tune CO adsorption strength and oxygen activation simultaneously. Chem. Commun..

[62] Zagalskaya A, Alexandrov V (2020). Role of defects in the interplay between adsorbate evolving and lattice oxygen mechanisms of the oxygen evolution reaction in RuO_2_ and IrO_2_. ACS Catal..

[63] Klyukin K (2019). Role of dissolution intermediates in promoting oxygen evolution reaction at RuO2(110) surface. J. Phys. Chem. C..

[64] Su, J. et al. Assembling ultrasmall copper-doped ruthenium oxide nanocrystals into hollow porous polyhedra: highly robust electrocatalysts for oxygen evolution in acidic media. *Adv. Mater*. 10.1002/adma.201801351 (2018). 10.1002/adma.201801351 29870585

[65] Kim J-Y (2001). 4d Electronic structure analysis of ruthenium in the perovskite oxides by Ru K- and L-edge XAS. J. Synchrotron Radiat..

[66] Stoerzinger KA (2017). Orientation-dependent oxygen evolution on RuO_2_ without lattice exchange. ACS Energy Lett..

[67] Harzandi AM (2021). Ruthenium core–shell engineering with nickel single atoms for selective oxygen evolution via nondestructive mechanism. Adv. Energy Mater..

[68] Scott SB (2022). The low overpotential regime of acidic water oxidation part II: trends in metal and oxygen stability numbers. Energy Environ. Sci..

[69] Wu, Z. Y. et al. Non-iridium-based electrocatalyst for durable acidic oxygen evolution reaction in proton exchange membrane water electrolysis. *Nat. Mater*. 10.1038/s41563-022-01380-5 (2022). 10.1038/s41563-022-01380-5 36266572

[70] Lazaridis T (2022). Capabilities and limitations of rotating disk electrodes versus membrane electrode assemblies in the investigation of electrocatalysts. Nat. Catal..

[71] Ehelebe K (2021). Limitations of aqueous model systems in the stability assessment of electrocatalysts for oxygen reactions in fuel cell and electrolyzers. Curr. Opin. Electrochem..

[72] Inaba M (2017). pH matters: the influence of the catalyst ink on the oxygen reduction activity determined in thin film rotating disk electrode measurements. J. Power Sources.

[73] Browne MP (2017). A mechanical, high surface area and solvent-free ‘powder-to-electrode’ fabrication method for screening OER catalysts. Electrochem. Commun..

[74] El-Sayed HA (2019). OER catalyst stability investigation using RDE technique: a stability measure or an artifact?. J. Electrochem. Soc..

[75] Browne MP, Mills A (2018). Determining the importance of the electrode support and fabrication method during the initial screening process of an active catalyst for the oxygen evolution reaction. J. Mater. Chem. A.

[76] Geiger S (2017). Catalyst stability benchmarking for the oxygen evolution reaction: the importance of backing electrode material and dissolution in accelerated aging studies. ChemSusChem.

[77] Geiger S (2018). The stability number as a metric for electrocatalyst stability benchmarking. Nat. Catal..

[78] Choi S (2020). Pt dopant: controlling the Ir oxidation states toward efficient and durable oxygen evolution reaction in acidic media. Adv. Funct. Mater..

[79] Zhu H (2021). High-entropy alloy stabilized active Ir for highly efficient acidic oxygen evolution. Chem. Eng. J..

[80] Liu X (2021). Restructuring highly electron-deficient metal-metal oxides for boosting stability in acidic oxygen evolution reaction. Nat. Commun..

[81] Zhang F-F (2021). Iridium oxide modified with silver single atom for boosting oxygen evolution reaction in acidic media. ACS Energy Lett..

[82] Shan J (2021). Short-range ordered iridium single atoms integrated into cobalt oxide spinel structure for highly efficient electrocatalytic water oxidation. J. Am. Chem. Soc..

[83] He J (2021). Regulating electron redistribution of intermetallic iridium oxide by incorporating Ru for efficient acidic water oxidation. Adv. Energy Mater..

[84] Wu D (2021). Efficient overall water splitting in acid with anisotropic metal nanosheets. Nat. Commun..

[85] Su H (2021). In-situ spectroscopic observation of dynamic-coupling oxygen on atomically dispersed iridium electrocatalyst for acidic water oxidation. Nat. Commun..

[86] Wang Y (2020). Ce-doped IrO_2_ electrocatalysts with enhanced performance for water oxidation in acidic media. ACS Appl. Mater. Interfaces.

[87] Dang Q (2021). Iridium metallene oxide for acidic oxygen evolution catalysis. Nat. Commun..

[88] Yin J (2020). Iridium single atoms coupling with oxygen vacancies boosts oxygen evolution reaction in acid media. J. Am. Chem. Soc..

[89] Li R (2021). IrW nanochannel support enabling ultrastable electrocatalytic oxygen evolution at 2 A cm^-2^ in acidic media. Nat. Commun..

[90] Fan Z (2021). Extraordinary acidic oxygen evolution on new phase 3R-iridium oxide. Joule.

[91] Kötz R (1984). In-situ identification of RuO_4_ as the corrosion product during oxygen evolution on ruthenium in acid media. J. Electroanal. Chem..

[92] Fang W-C (2006). Effect of temperature annealing on capacitive and structural properties of hydrous ruthenium oxides. J. Power Sources.

[93] Iliev, M. N. et al. Raman spectroscopy of Ca_3_Ru_2_O_7_: phonon line assignment and electron scattering. *Phys. Rev. B* **71**, 214305 (2005).

[94] Kumar V (2021). Origin of intense blue-green emission in SrTiO_3_ thin films with implanted nitrogen ions: an investigation by synchrotron-based experimental techniques. Phys. Rev. B.

[95] Ye Y (2017). X-ray spectroscopies studies of the 3d transition metal oxides and applications of photocatalysis. MRS Commun..

[96] Pivovar, B. *H2NEW: Hydrogen (H2) from Next-generation Electrolyzers of Water Overview*. (US Department of Energy, 2021)

[97] Kim H (2021). Dendritic gold-supported iridium/iridium oxide ultra-low loading electrodes for high-performance proton exchange membrane water electrolyzer. Appl. Catal. B.

[98] Hegge F (2020). Efficient and stable low iridium loaded anodes for PEM water electrolysis made possible by nanofiber interlayers. ACS Appl. Energy Mater..

[99] Feng Q (2020). Oxygen vacancy engineering of yttrium ruthenate pyrochlores as an efficient oxygen catalyst for both proton exchange membrane water electrolyzers and rechargeable zinc-air batteries. Appl. Catal. B.

[100] Choe S (2018). Electrodeposited IrO_2_/Ti electrodes as durable and cost-effective anodes in high-temperature polymer-membrane-electrolyte water electrolyzers. Appl. Catal. B.

[101] Böhm D (2021). Highly conductive titania supported iridium oxide nanoparticles with low overall iridium density as OER catalyst for large-scale PEM electrolysis. Appl. Mater. Today.

[102] Pham CV (2020). IrO_2_ coated TiO_2_ core-shell microparticles advance performance of low loading proton exchange membrane water electrolyzers. Appl. Catal. B.

[103] Oh JH (2021). Activity and stability of Ir-based gas diffusion electrode for proton exchange membrane water electrolyzer. Chem. Eng. J..

[104] Islam J (2021). Enhancing the activity and durability of iridium electrocatalyst supported on boron carbide by tuning the chemical state of iridium for oxygen evolution reaction. J. Power Sources.

[105] Li G (2016). Iridium-Tin oxide solid-solution nanocatalysts with enhanced activity and stability for oxygen evolution. J. Power Sources.

[106] Jiang G (2019). An effective oxygen electrode based on Ir0.6Sn0.4O2 for PEM water electrolyzers. J. Energy Chem..

[107] Wang L (2017). Highly active anode electrocatalysts derived from electrochemical leaching of Ru from metallic Ir0.7Ru0.3 for proton exchange membrane electrolyzers. Nano Energy.

[108] Knoppel J (2021). On the limitations in assessing stability of oxygen evolution catalysts using aqueous model electrochemical cells. Nat. Commun..

[109] Kresse G, Furthmüller J (1996). Efficiency of ab-initio total energy calculations for metals and semiconductors using a plane-wave basis set. Comput. Mater. Sci..

[110] Neurock M (2009). A first principles comparison of the mechanism and site requirements for the electrocatalytic oxidation of methanol and formic acid over Pt. Faraday Discuss.

[111] Jiao M (2016). First-principles study on nitrobenzene-doped graphene as a metal-free electrocatalyst for oxygen reduction reaction. J. Phys. Chem. C..

[112] Blöchl PE (1994). Projector augmented-wave method. Phys. Rev. B.

[113] Kresse G, Furthmüller J (1996). Efficient iterative schemes for ab initio total-energy calculations using a plane-wave basis set. Phys. Rev. B.

[114] Kresse G, Hafner J (1993). Ab initio molecular dynamics for liquid metals. Phys. Rev. B.

[115] Kresse G, Hafner J (1994). Ab initio molecular-dynamics simulation of the liquid-metal–amorphous-semiconductor transition in germanium. Phys. Rev. B.

[116] Monkhorst HJ, Pack JD (1976). Special points for Brillouin-zone integrations. Phys. Rev. B.

