# Supplementary material for: Customized reaction route for ruthenium oxide towards stabilized water oxidation in high-performance PEM electrolyzers
Source: Nat Commun. 2023 Feb 15;14:843. doi: 10.1038/s41467-023-36380-9 (PMC9932065; doi:10.1038/s41467-023-36380-9)
Supplement: Supplementary file 1 — Supplementary Information [file 41467_2023_36380_MOESM1_ESM.docx]

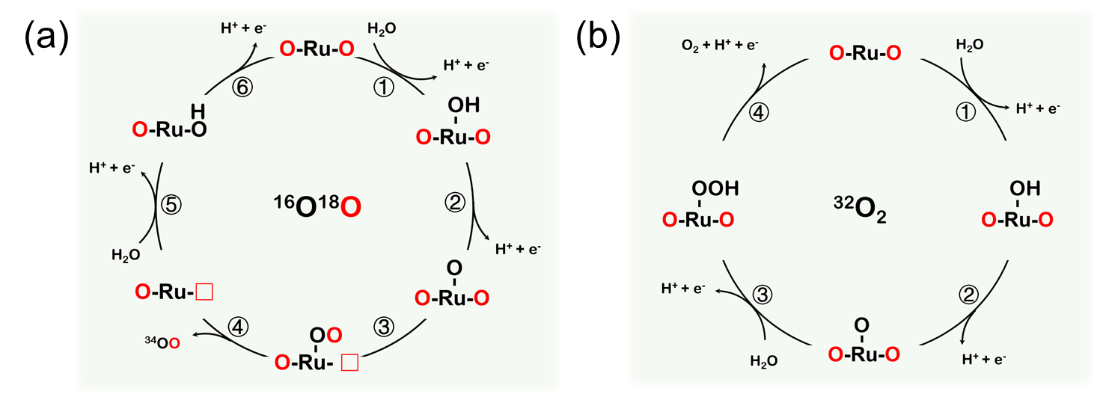


**Supplementary Fig. 1 | Catalytic reaction paths.** (a) Lattice oxygen mediated mechanism (LOM), (b) Absorbate evolution mechanism (AEM).

**Supplementary Note 1 | Structure model of M_0.5_Ru_0.5_O_2_.**

The construction of structural model plays a vital role in theoretical calculation. Homogeneous structure model (half Ru and half M at each layer, Supplementary Fig. 2), layered structure (one layer RuO_2_ and one layer MO_2_, Supplementary Fig. 3) and segregated structure (Supplementary Fig. 4) are constructed for stability comparison. It is worth noting that the initial structure of M_0.5_R_0.5_O_2_ was expanded by 2 × 2 × 2 based on the unit cell of RuO_2_ (two layer RuO_2_), which set a limitation that the maximum extent of phase segregation in M_0.5_R_0.5_O_2_ is two layers of RuO_2_ and two layers of MO_2_ (Supplementary Fig. 4). After structure optimization and energy calculation, we find for both Ce_0.5_Ru_0.5_O_2_ and Cr_0.5_Ru_0.5_O_2_, the energy of homogeneous structure model with half Ru and half M at each layer is lower than the layered and segregated structure (Supplementary Fig. 5a, c), implying the homogeneous model is more stable and reasonable for conducting theoretical computation.

For Sn_0.5_Ru_0.5_O_2_, the layered structure gives the highest energy of -321.07 eV, which is 2.91eV and 2.95 eV higher than that of homogeneous model (-323.98 eV) and segregated structure (-324.02 eV) (Supplementary Fig. 5b). Considering the minimal energy difference (0.04 eV) between homogeneous model and segregated structure, we compared the theoretical activity of these two structures under AEM and LOM pathways. As shown in Supplementary Fig. 6, both the PDS (OOH* and Ov formation for AEM and LOM, respectively) and Δ*G*_PDS_ (1.84 eV *vs.* 1.83 eV for AEM, 2.07 eV, 1.97 eV for LOM) on the two structures are similar, indicating these two structures are identical in revealing the theoretical catalytic activity. Thus, to keep the consistency of computation on M_0.5_R_0.5_O_2_, the homogeneous structure of Sn_0.5_Ru_0.5_O_2_ is selected for discussion in the manuscript.


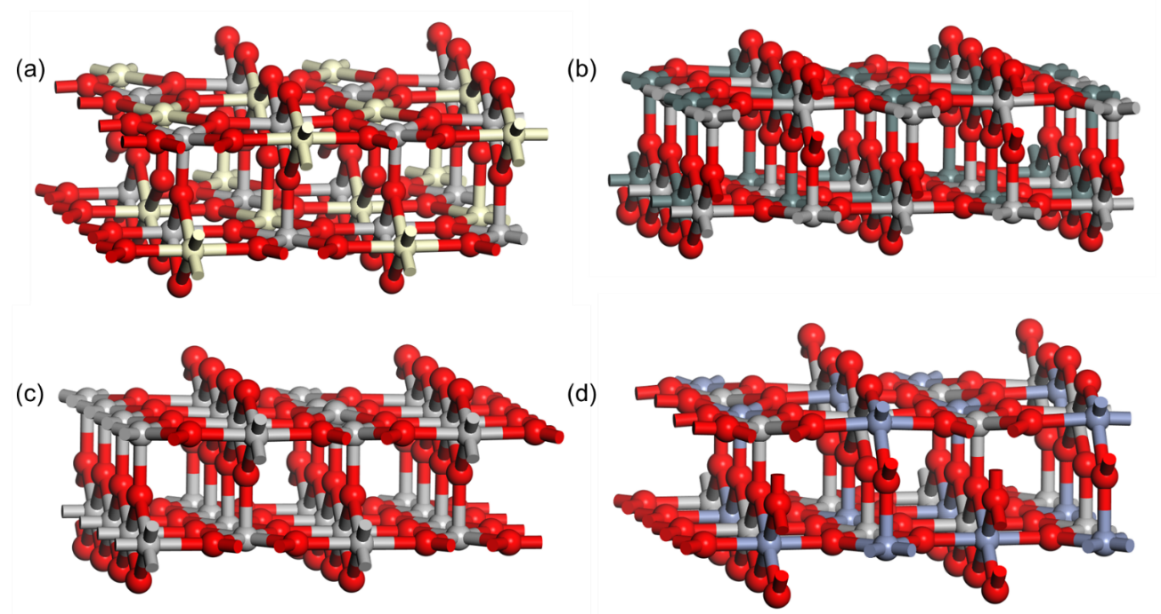


**Supplementary Fig. 2 | Structure models for DFT calculation.** (a) Ce_0.5_Ru_0.5_O_2_, (b) Sn_0.5_Ru_0.5_O_2_, (c) RuO_2_, (d) Cr_0.5_Ru_0.5_O_2_. The M and Ru are homogeneously distributed in each layer.


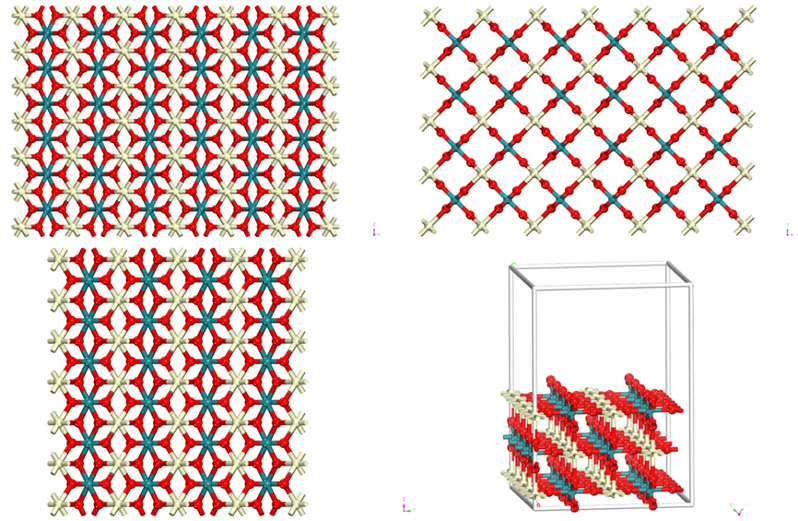


**Supplementary Fig. 3 |** Structure model of layered M_0.5_R_0.5_O_2_ (take Ce_0.5_Ru_0.5_O_2_ as a sample), where the MO_2_ and RuO_2_ are stacked in single layer.


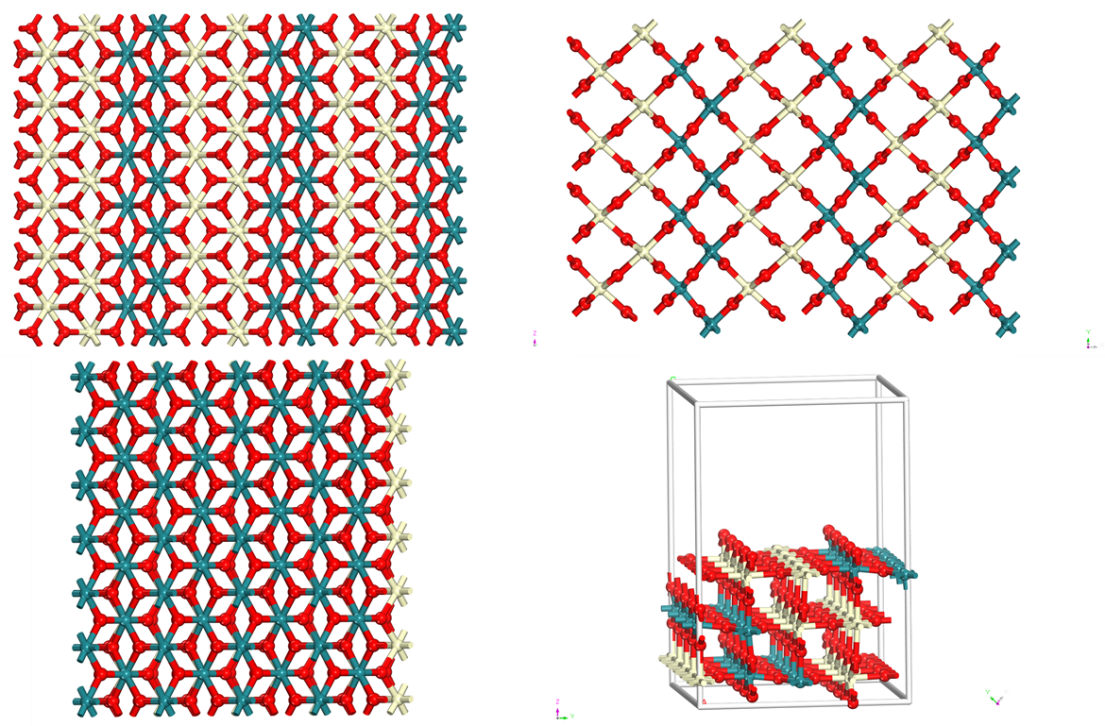


**Supplementary Fig. 4 |** Structure model of segregated M_0.5_R_0.5_O_2_ (take Ce_0.5_Ru_0.5_O_2_ as a sample), where the MO_2_ and RuO_2_ are stacked in two layers.


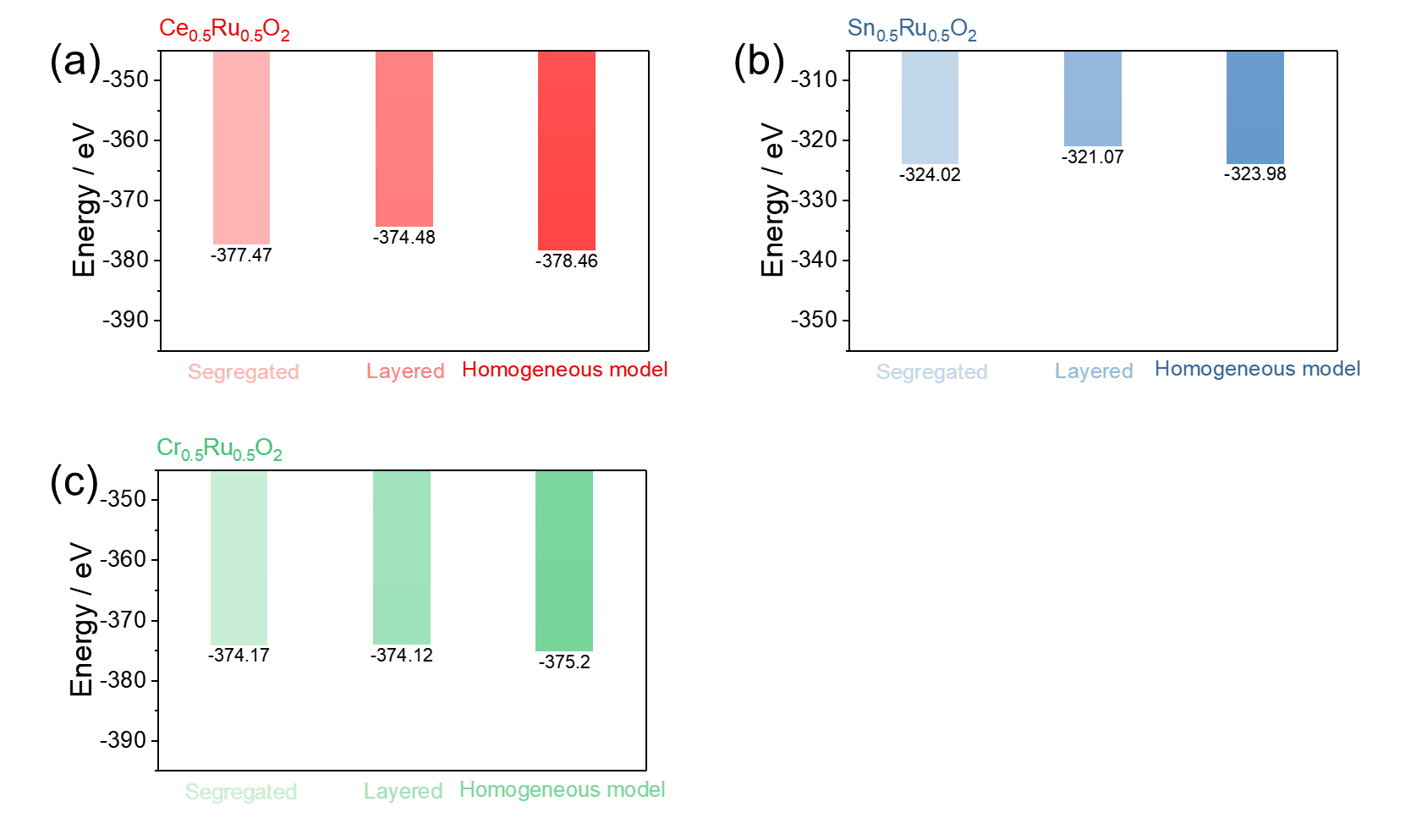


**Supplementary Fig. 5 |** Energy of the segregated, layered and homogeneous structure model of (a) Ce_0.5_Ru_0.5_O_2_, (b) Sn_0.5_Ru_0.5_O_2_ and (c) Cr_0.5_Ru_0.5_O_2_ after optimization.


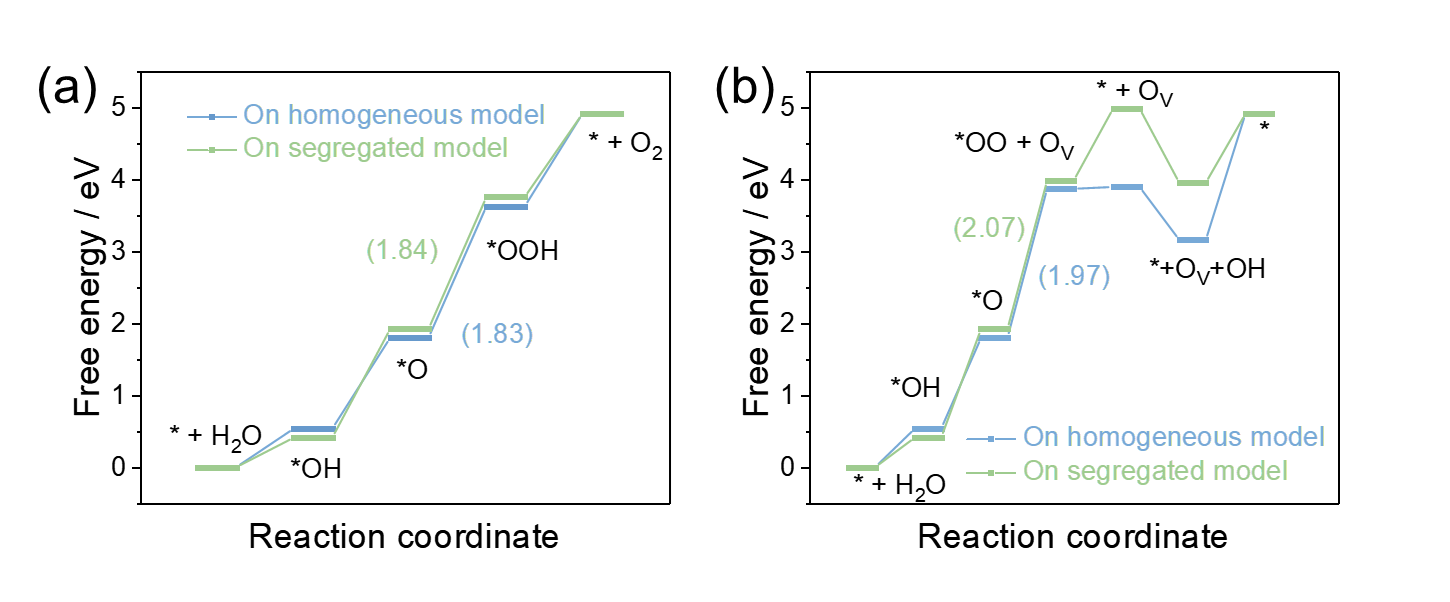


**Supplementary Fig. 6 |** Free energy diagram of (a) AEM path and (b) LOM path on segregated and homogeneous model. Data derived from Supplementary Fig. 14 and 28.

**Supplementary Note 2 | Comparison on the theoretical activity on M and Ru sites.**

The energetic pathway of AEM and LOM on the guest cations sites (Ce, Sn and Cr) are calculated and compared with that on Ru sites (Supplementary Fig. 7-13). On the reaction path of AEM, the PDS is the step of OOH* formation from O* for all samples with guest M cations as active sites, giving Δ*G*_PDS_ of 2.65 eV, 2.32 eV and 2.42 eV for Ce, Sn and Cr, respectively, which is originated from the too strong oxygen species binding energy, as suggested by the low value of Δ*G*_O*_-Δ*G*_OH*_ on CeO_2_, SnO_2_ and CrO_2_^1^. On LOM, the PDS is the step of O-O bonding with the formation of lattice oxygen vacancy (Supplementary Fig. 10, 11) for Ce_0.5_Ru_0.5_O_2_ and Sn_0.5_Ru_0.5_O_2_, giving theoretical overpotential of 1.6 V and 1.05 V, which is in line with the high value of Δ*G*_VO_ and the free energy diagram with Ru as active sites (Supplementary Fig. 23 and 28-29). However, for Cr_0.5_Ru_0.5_O_2_, originating from the easy Ov formation (Supplementary Fig. 12), the PDS shifted from the step of lattice oxygen formation to the step of OO* desorption on Cr (Δ*G*_PDS_ = 1.98 eV), implying the strong binding between Cr and oxygen species, which is in line with the previous reports^1^. As the theoretical activity on the guest cations sites is extremely poor compared to that on the host Ru sites for both AEM and LOM (Supplementary Fig. 13), it is safe to claim that the activity of the MRuOx derives from the Ru sites.


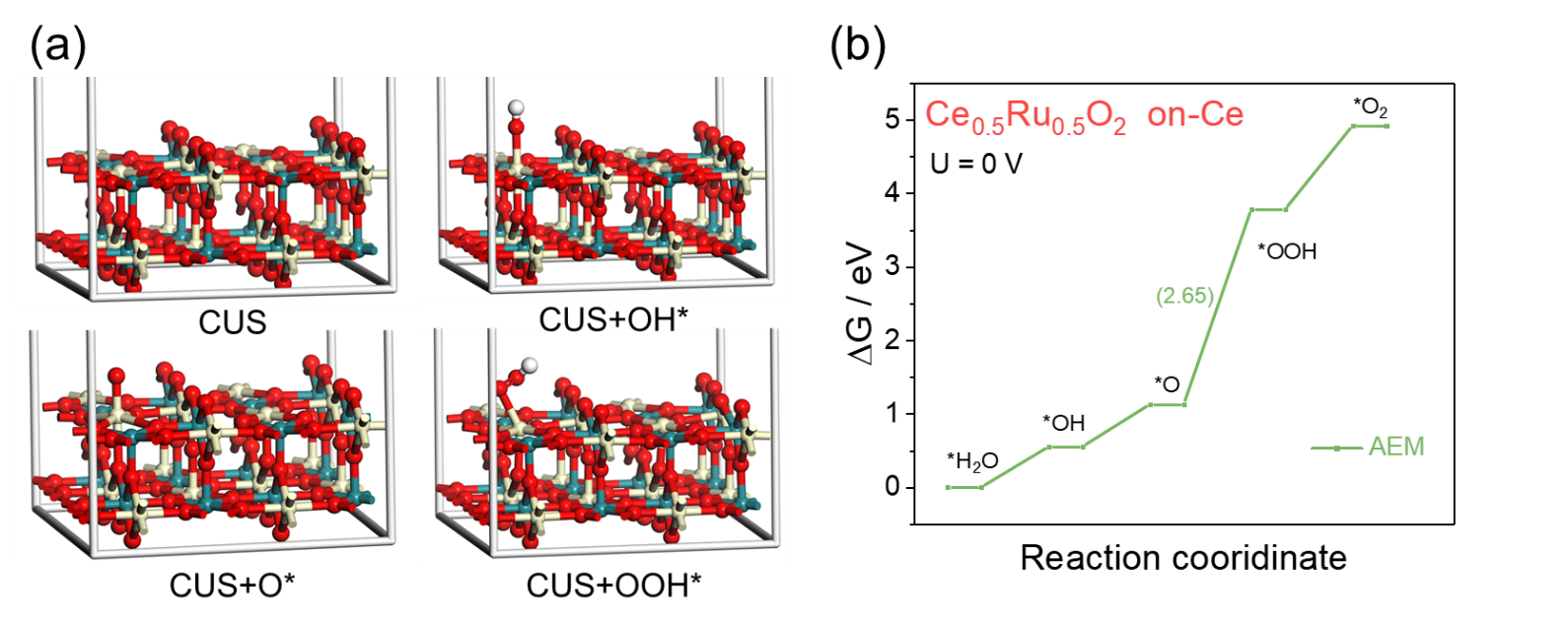


**Supplementary Fig. 7 | The free energy diagram of the AEM path on Ce site for Ce_0.5_Ru_0.5_O_2_.** (a) Structures of the key intermediates on AEM path, (b) Gibbs free energy diagram at 0 V based on AEM.


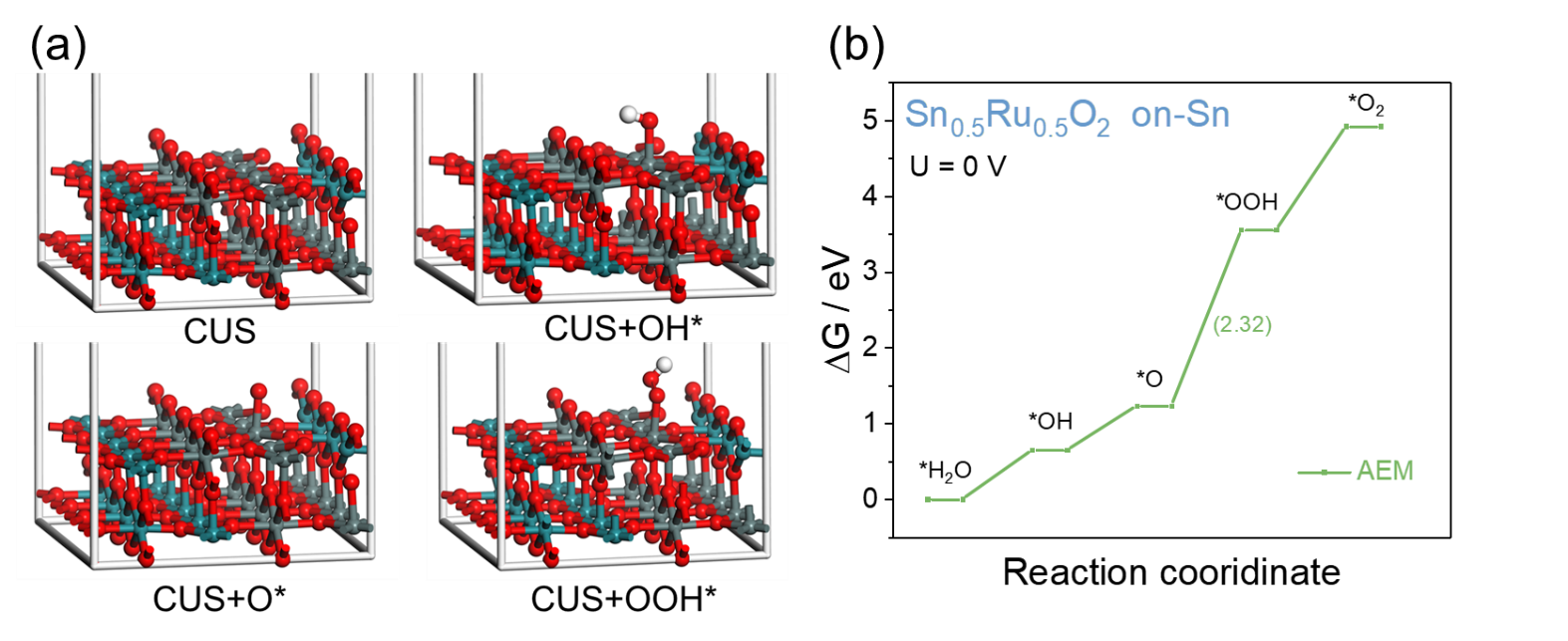


**Supplementary Fig. 8 | The free energy diagram of the AEM path on Sn site for Sn_0.5_Ru_0.5_O_2_.** (a) Structures of the key intermediates on AEM path, (b) Gibbs free energy diagram at 0 V based on AEM.


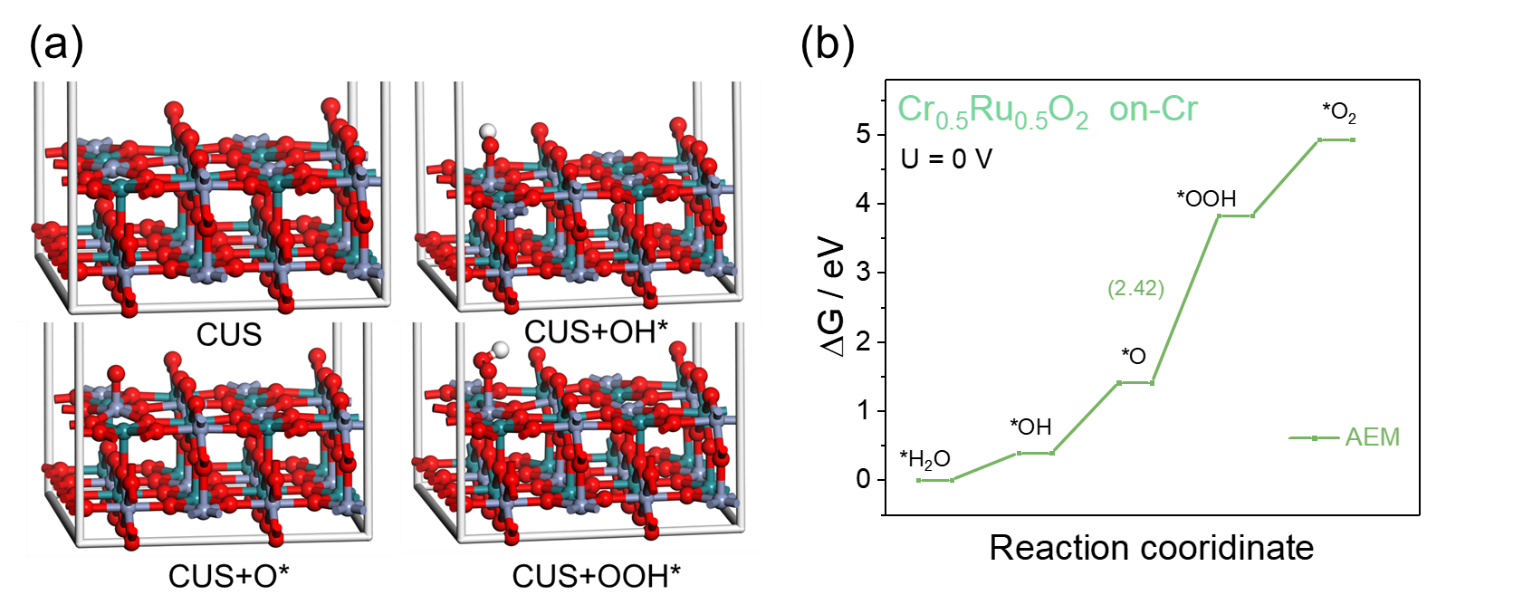


**Supplementary Fig. 9 | The free energy diagram of the AEM path on Cr site for Cr_0.5_Ru_0.5_O_2_.** (a) Structures of the key intermediates on AEM path, (b) Gibbs free energy diagram at 0 V based on AEM.


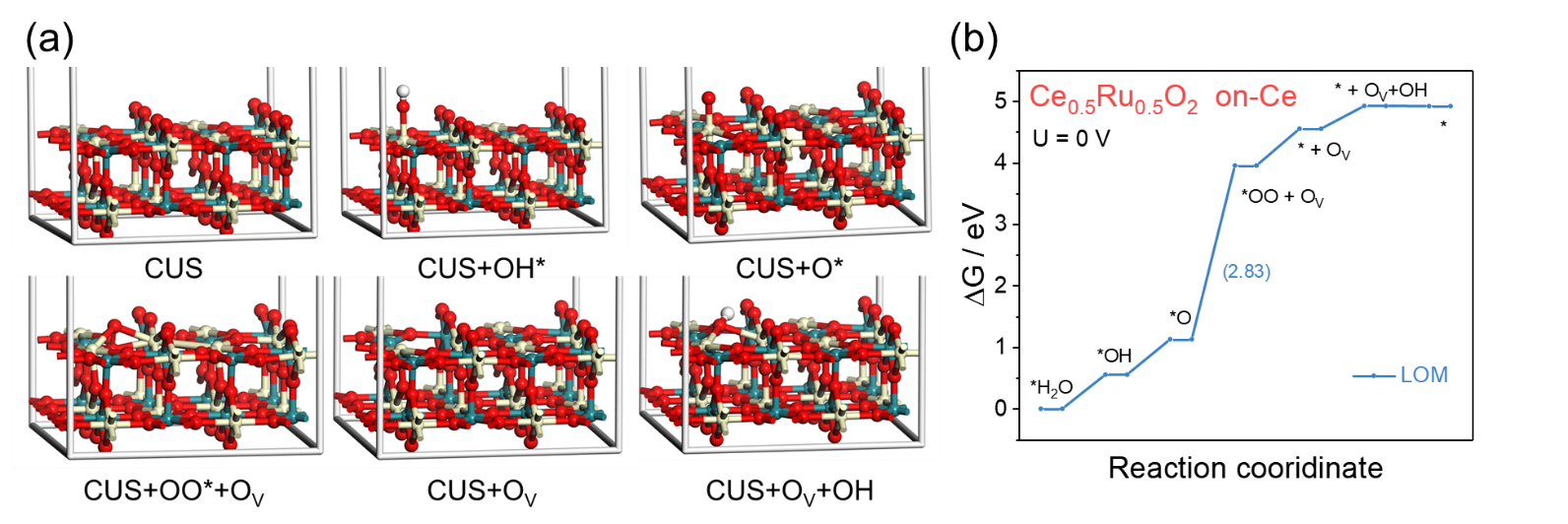


**Supplementary Fig. 10 | The free energy diagram of the LOM path on Ce site for Ce_0.5_Ru_0.5_O_2_.** (a) Structures of the key intermediates on LOM path, (b) Gibbs free energy diagram at 0 V based on LOM.


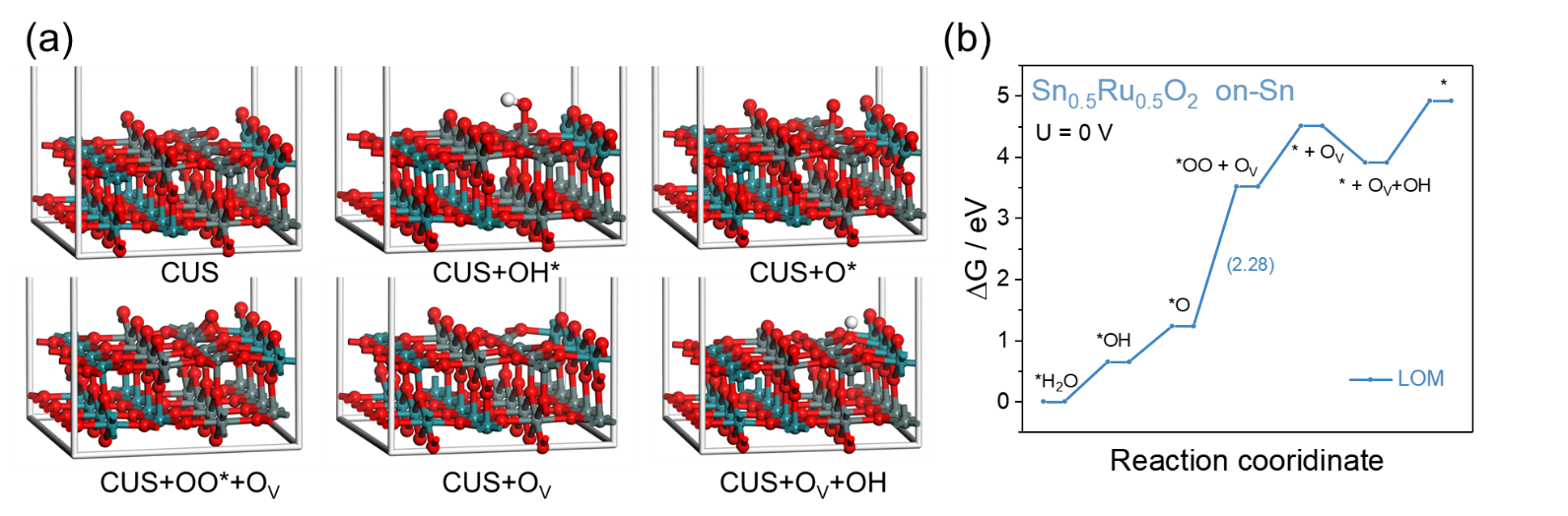


**Supplementary Fig. 11 | The free energy diagram of the LOM path on Sn site for Sn_0.5_Ru_0.5_O_2_.** (a) Structures of the key intermediates on LOM path, (b) Gibbs free energy diagram at 0 V based on LOM.


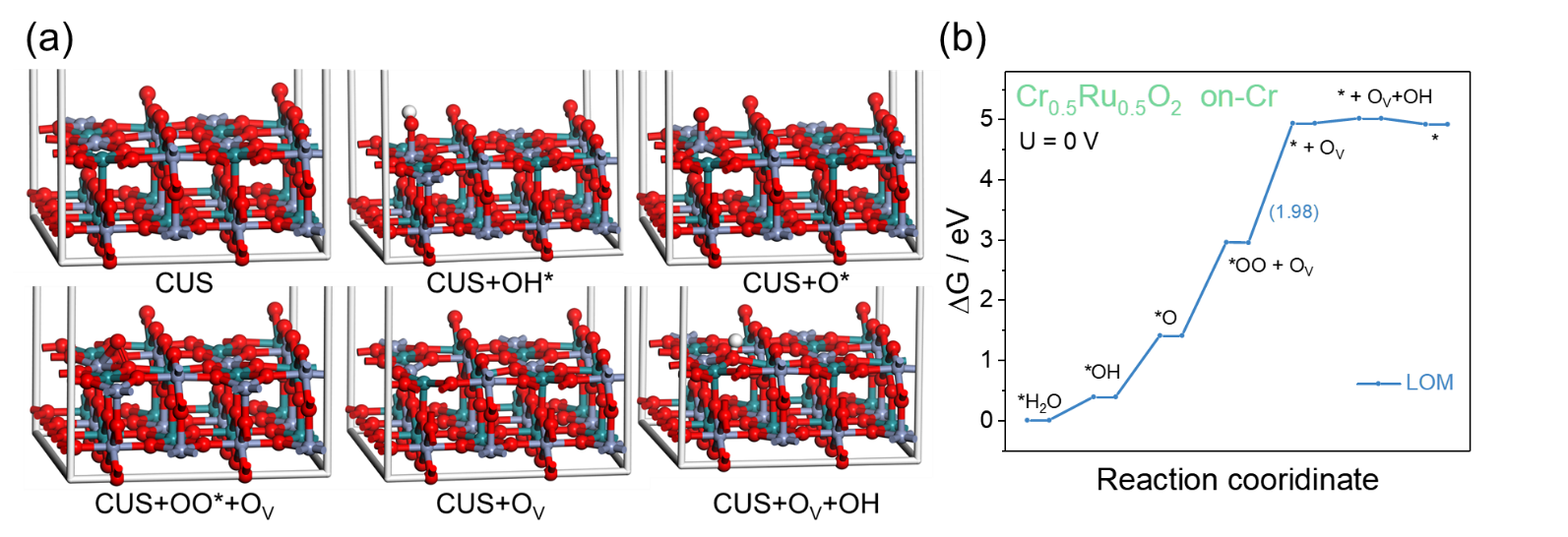


**Supplementary Fig. 12 | The free energy diagram of the LOM path on Cr site for Cr_0.5_Ru_0.5_O_2_.** (a) Structures of the key intermediates on LOM path, (b) Gibbs free energy diagram at 0 V based on LOM.


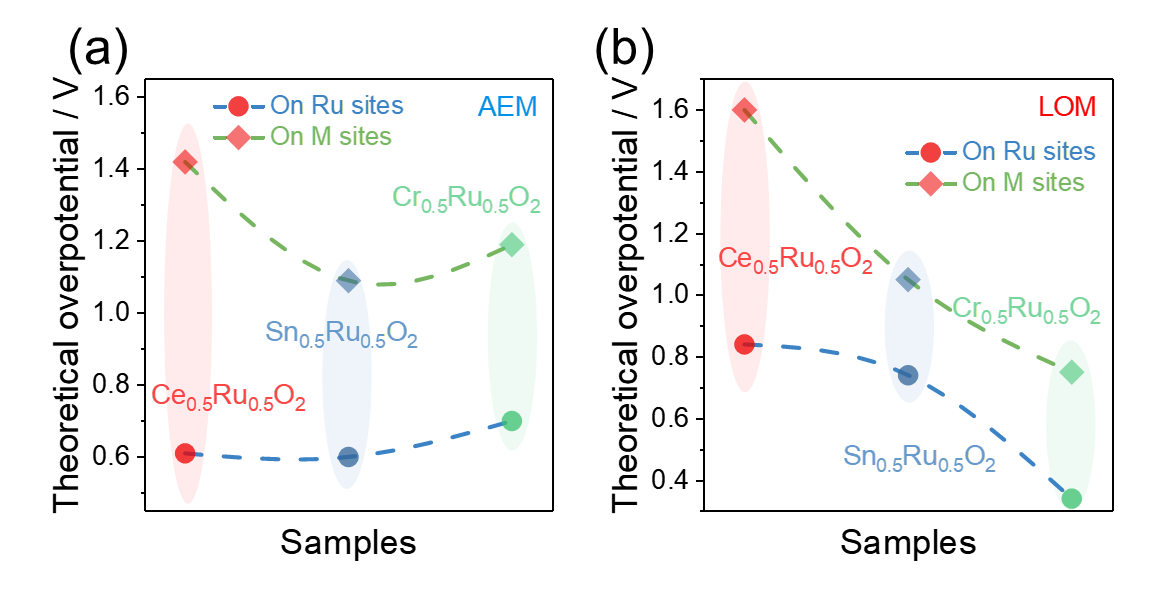


**Supplementary Fig. 13 |** Theoretical overpotential of (a) AEM and (b) LOM on the surface of M_0.5_Ru_0.5_O_2_, with M and Ru considered as the active sites. The data is derived from Supplementary Fig. 14, 15, 17, 28, 29, 31.


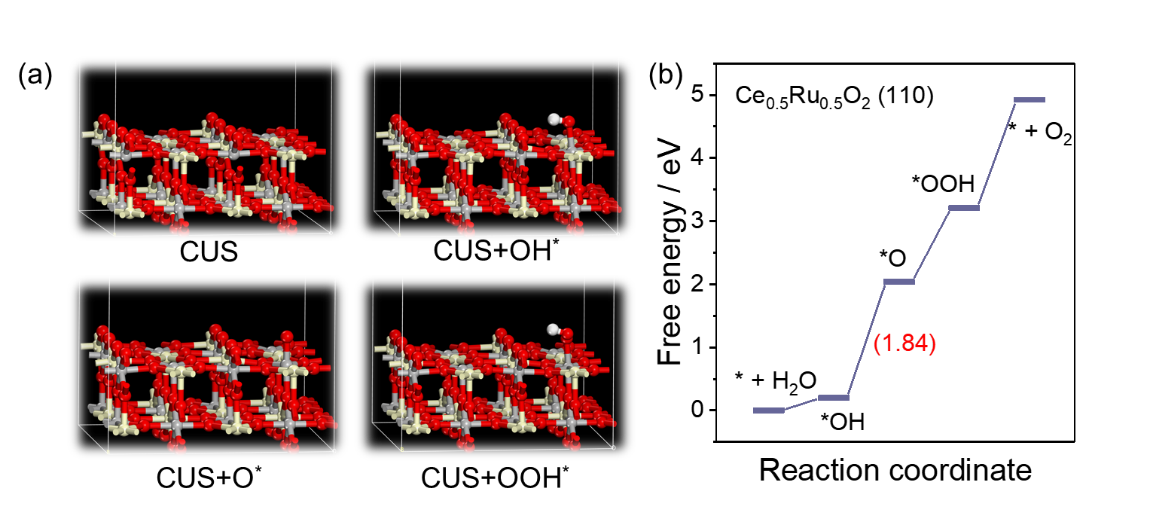


**Supplementary Fig. 14 |** **The free energy diagram of the AEM path on Ru site for Ce_0.5_Ru_0.5_O_2_.** (a) Structures of the key intermediates on AEM path, (b) Gibbs free energy diagram at 0 V based on AEM.


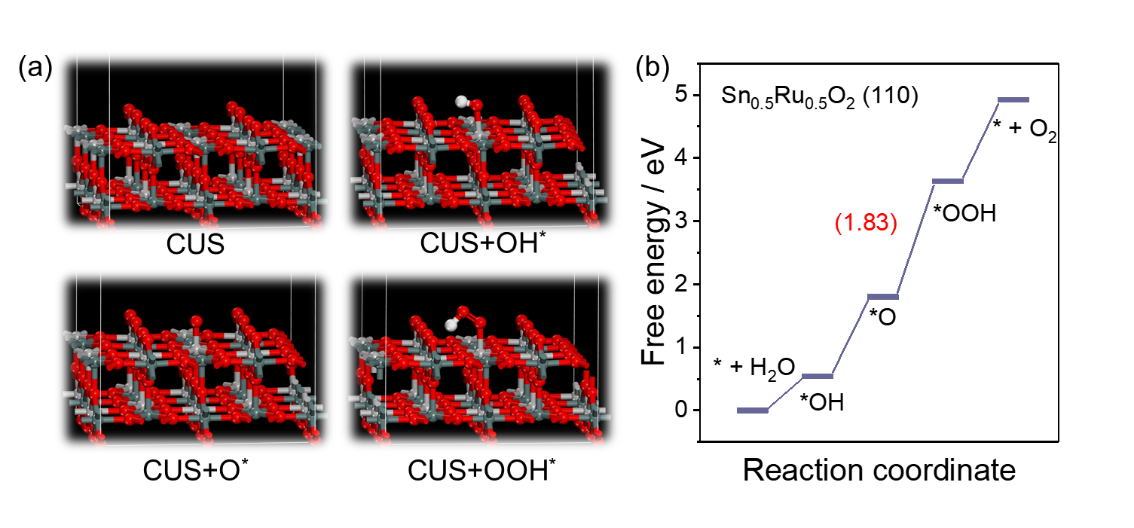


**Supplementary Fig. 15 | The free energy diagram of the AEM path on Ru site for Sn_0.5_Ru_0.5_O_2_.** (a) Structures of the key intermediates on AEM path, (b) Gibbs free energy diagram at 0 V based on AEM.


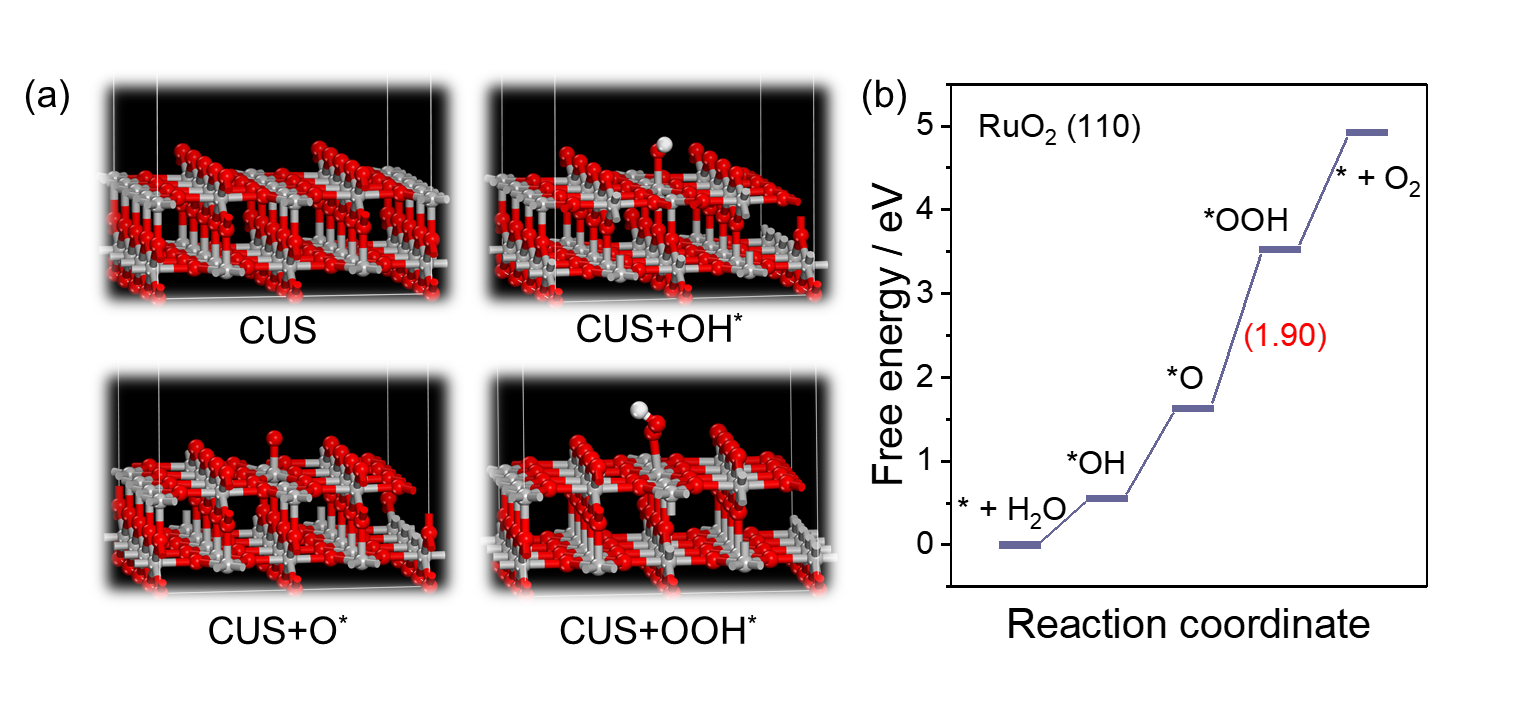


**Supplementary Fig. 16 | The free energy diagram of the AEM path on Ru site for RuO_2_.** (a) Structures of the key intermediates on AEM path, (b) Gibbs free energy diagram at 0 V based on AEM.


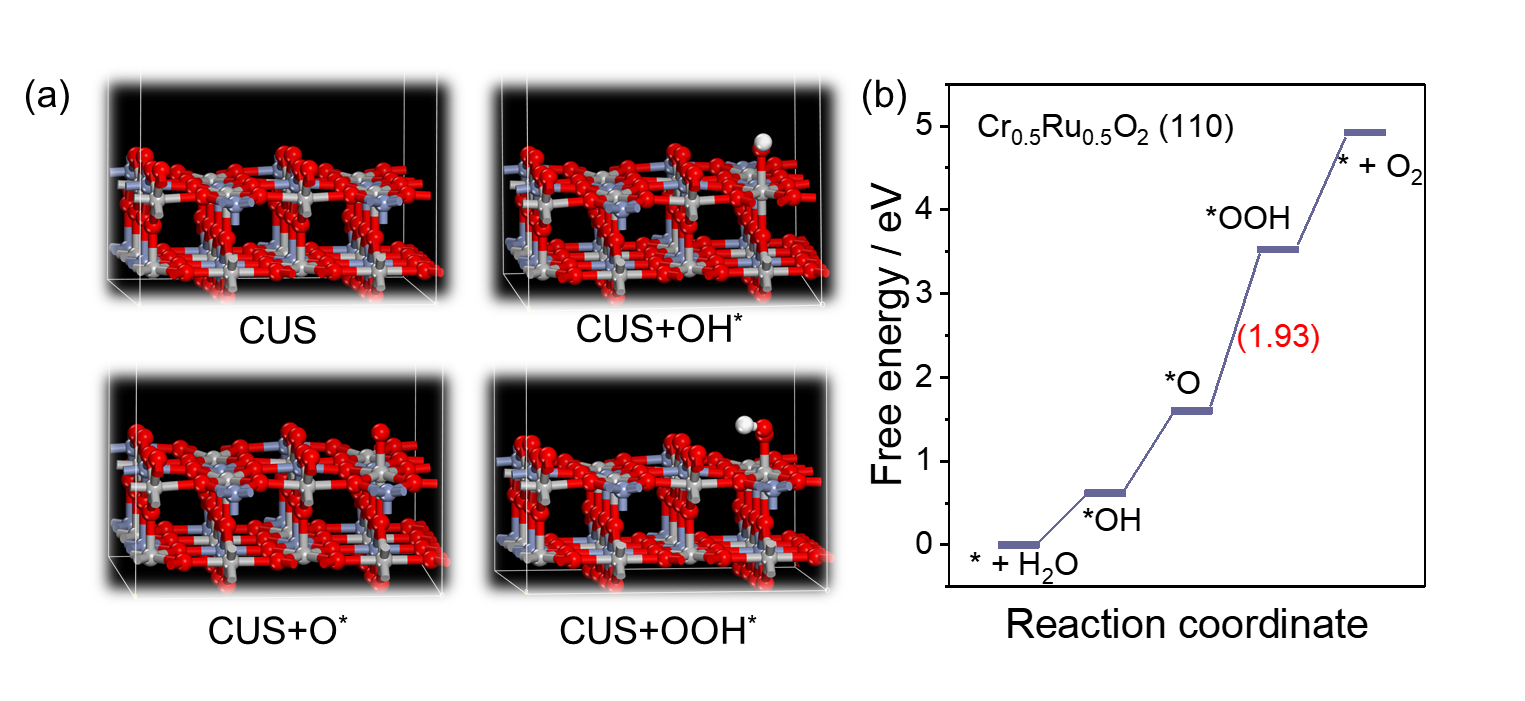


**Supplementary Fig. 17 | The free energy diagram of the AEM path on Ru site for Cr_0.5_Ru_0.5_O_2_.** (a) Structures of the key intermediates on AEM path, (b) Gibbs free energy diagram at 0 V based on AEM.


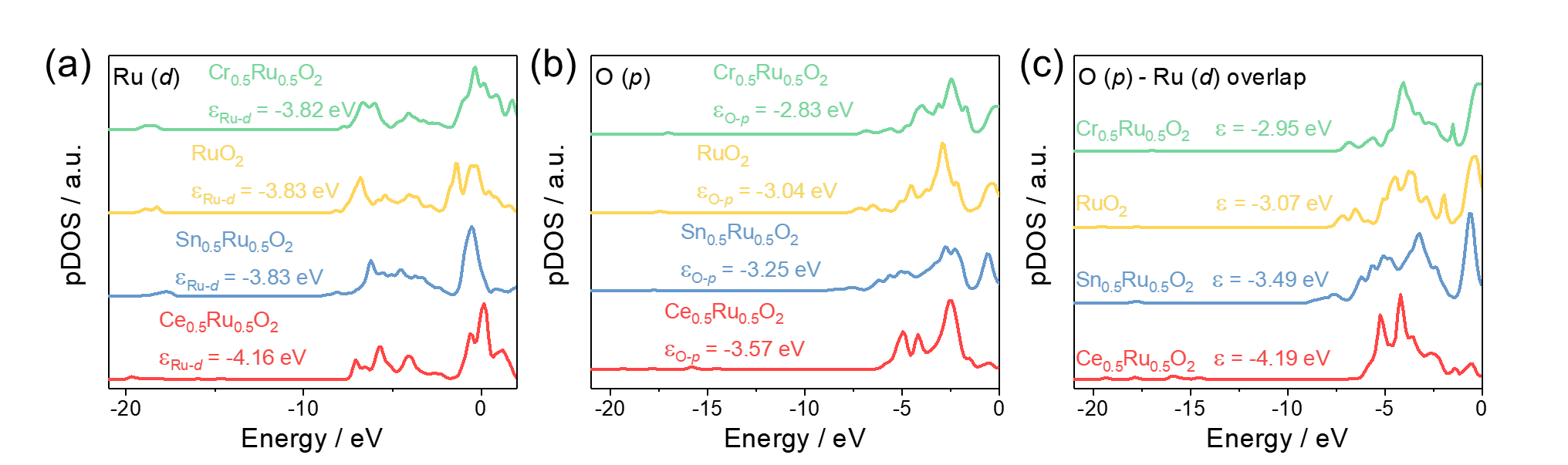


**Supplementary Fig. 18 | pDOS of M_0.5_Ru_0.5_O_2_.** (a) Ru *d* band, (b) O *p* band and (c) O (*p*) – Ru (*d*) overlap of M_0.5_Ru_0.5_O_2_ and the corresponding band center (ε).


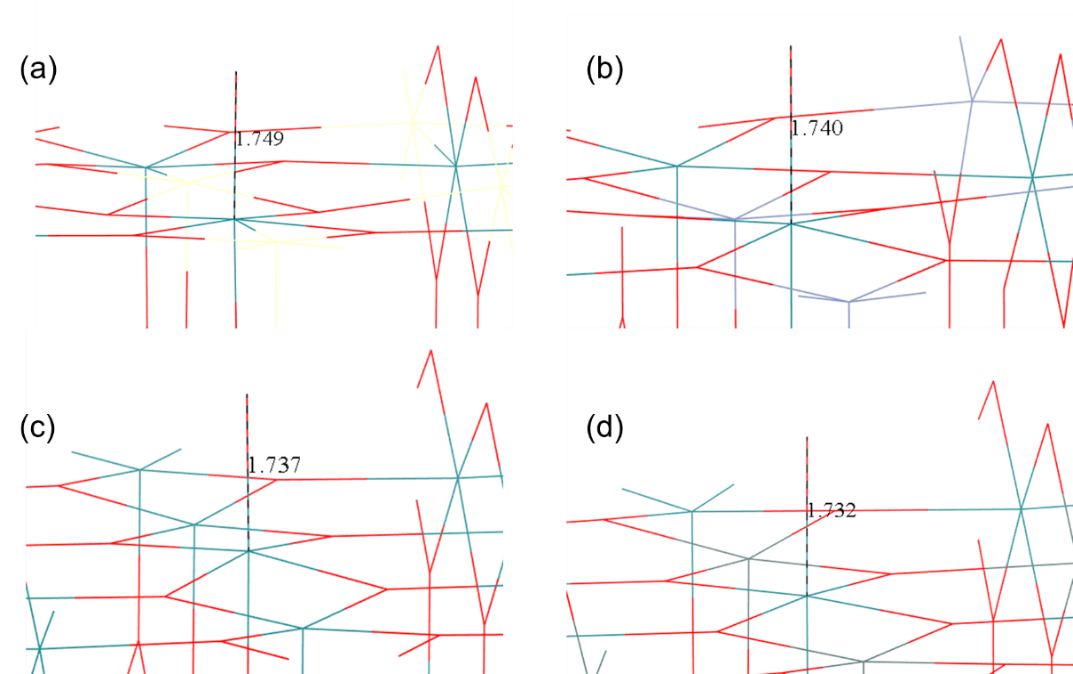


**Supplementary Fig. 19 | Bond length of Ru-O* on M_0.5_Ru_0.5_O_2_.** (a) Ce_0.5_Ru_0.5_O_2_, (b) Sn_0.5_Ru_0.5_O_2_, (c) RuO_2_, (d) Cr_0.5_Ru_0.5_O_2_.


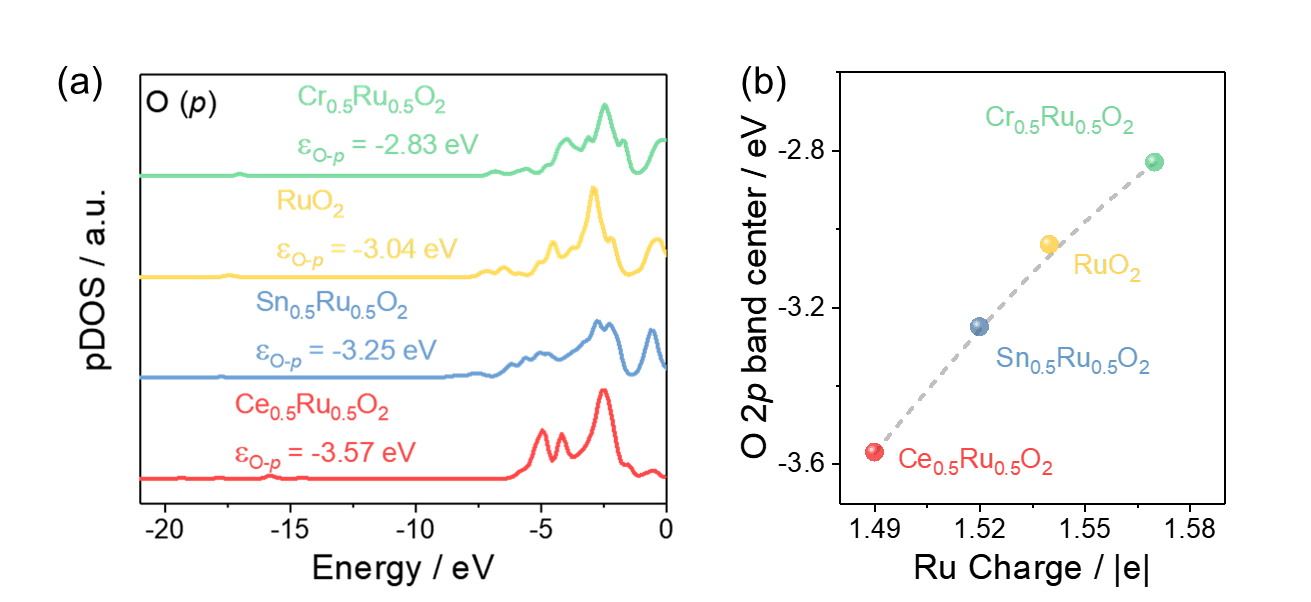


**Supplementary Fig. 20 | pDOS of M_0.5_Ru_0.5_O_2_.** (a) O (*p*) pDOS of M_0.5_Ru_0.5_O_2_ and (b) the corresponding *p* band center (ε_O-_*_p_*).


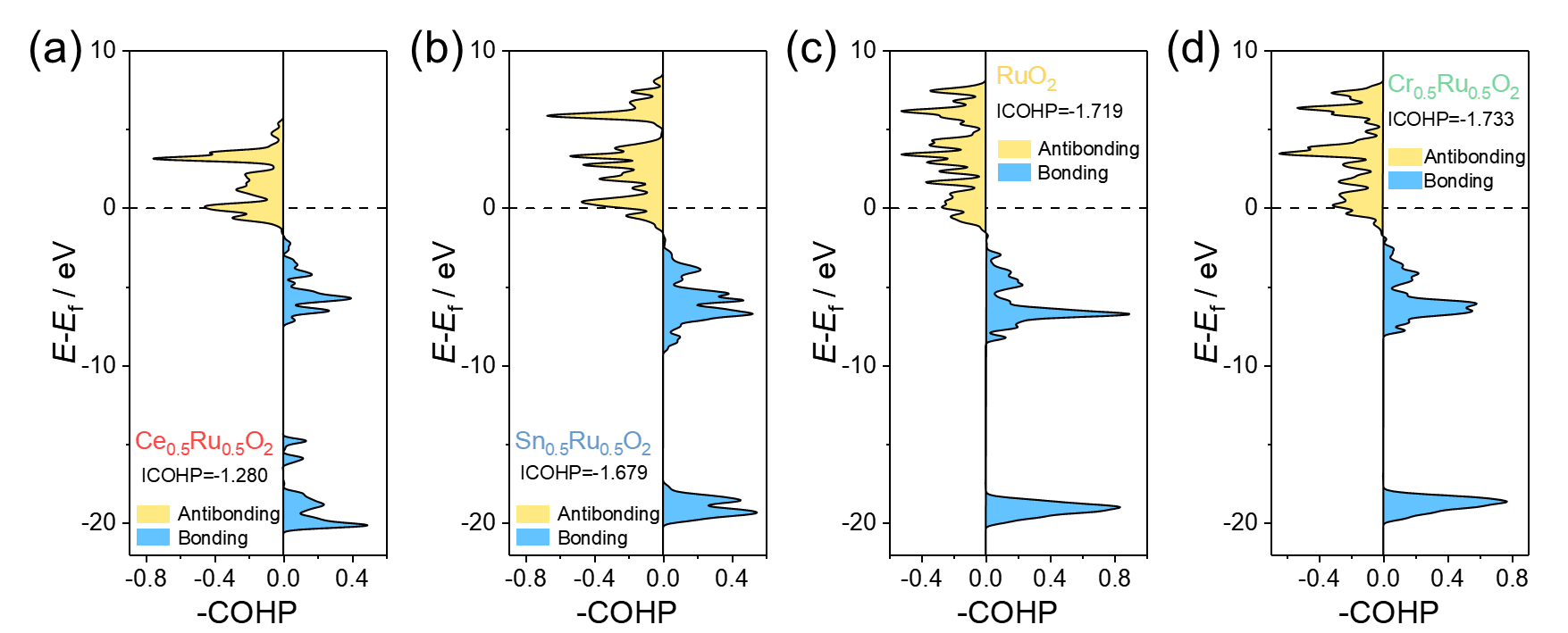


**Supplementary Fig. 21 | Calculated COHP of M_0.5_Ru_0.5_O_2_.** (a) Ce_0.5_Ru_0.5_O_2_, (b) Sn_0.5_Ru_0.5_O_2_, (c) RuO_2_, (d) Cr_0.5_Ru_0.5_O_2_.


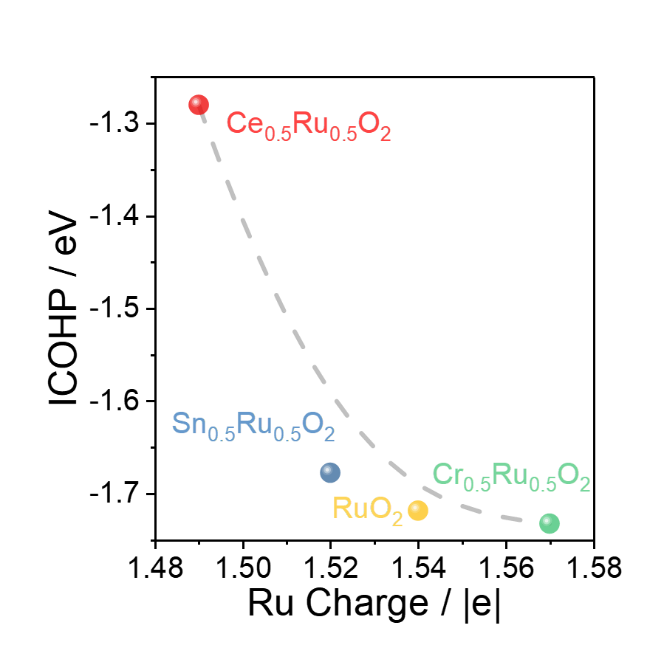


**Supplementary Fig. 22 | ICOHP of M_0.5_Ru_0.5_O_2_ on the scale of Ru charge.**

**Supplementary Fig. 23 | The formation energy of lattice oxygen vacancy on M_0.5_Ru_0.5_O_2_.**

**Supplementary Note 3 | The relation between the structure relaxation and Ru charge.**

Statics on the Ru-O bond length of M_0.5_Ru_0.5_O_2_ indicates that with the decrease of dopant radius from 1.01 (Ce^4+^) to 0.69 (Cr^4+^), the Ru-O bond length ranges from +2 % (tensile strain) to -1 % (compressive strain). According to the previous reports, the Ru-O bonding properties such as vacancy formation energy is also affected by the structure relaxation^2^, we thus performed DFT calculations on the strained RuO_2_ to clarify its contribution and relationship with Ru charge. First of all, strain along X and Y is applied on RuO_2_ (110) to ensure the variation range of +2 % to -1 % is included (Supplementary Fig. 24). Then, COHP analysis is performed to access the Ru-O bonding nature in the strained structure (Supplementary Fig. 25). Interestingly, the ICOHP between Ru and O gradually shifted from -1.656 to -1.869 with decreased Ru-O bond length (from +1.97 % to -4.58 %, Supplementary Fig. 26a), implying the enhanced Ru-O bond covalency, which is in good accordance with the increased formation energy of Ru vacancies (Δ*G*_VRu_, Supplementary Fig. 26b). By calculating Δ*G*_VO_, we found the easier formation of Ov with both elongated and compressed Ru-O bonds (Supplementary Fig. 26b). For shortened Ru-O bond length, the lowered Δ*G*_VO_ value arises from the promoted lattice oxygen redox as indicated by the up-shifted O 2*p* band center (Supplementary Fig. 26c, d). While for Ru-O bond with tensile strain, the lowered Δ*G*_VO_ value derives from the distortion of the crystal structure according to the previous reports, which may be the origin of the lowered Δ*G*_VO_ value and higher O_V_ content of Ce_0.5_Ru_0.5_O_2_ (Supplementary Fig. 23 and 54).

To clarify the relation between structural relaxation and Ru charge, Bader charge analysis is performed on the strained RuO_2_ (110). As the Ru-O bond length decreases from +1.97 % to -4.58 %, the Ru charge increase from 1.5 to 1.61 (Supplementary Fig. 27a), giving the ICOHP, Δ*G*_VRu_, Δ*G*_VO_ and O 2*p* band center also scale with the Ru charge in the strained structure (Supplementary Fig. 27b-d). As these variation trends are in high accordance with that obtained on M_0.5_Ru_0.5_O_2_ (Supplementary Fig. 21-23 and Fig. 2g), we believe the structural relaxation also regulates the Ru-O bonding nature by affecting the Ru charge. Therefore, though the variation trend in Ru charge is designed to tailored by the difference in electronegativity of M^4+^, the Ru charge obtained on M_0.5_Ru_0.5_O_2_ indeed derive from a joint effect of electronic and structural factor.


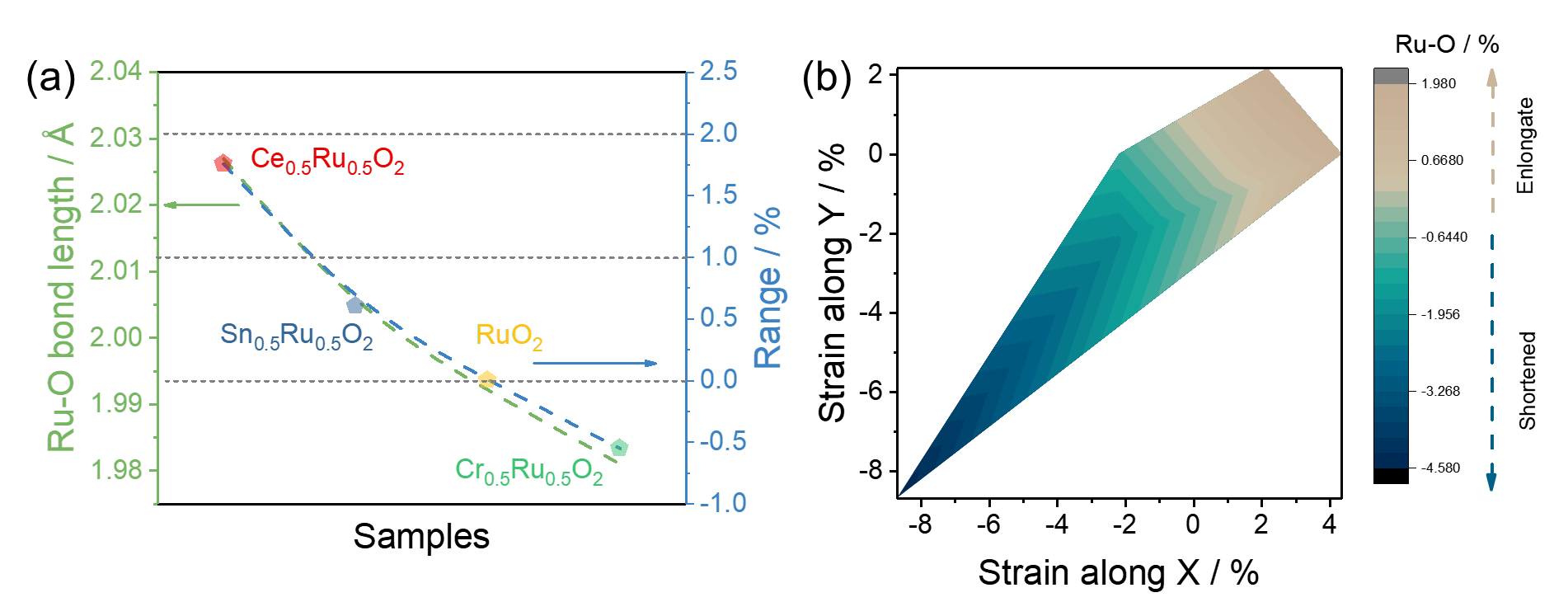


**Supplementary Fig. 24 | Strained RuO_2_ (110) for DFT calculation.** (a) Ru-O bond length in M_0.5_Ru_0.5_O_2_, with the tensile or compressive range (calculated based on that of RuO_2_) presented on the ringt Y axis; (b) the Ru-O bond length of tensiled or compressed RuO_2_ (110) on the scale of strain (along X or Y) applied.


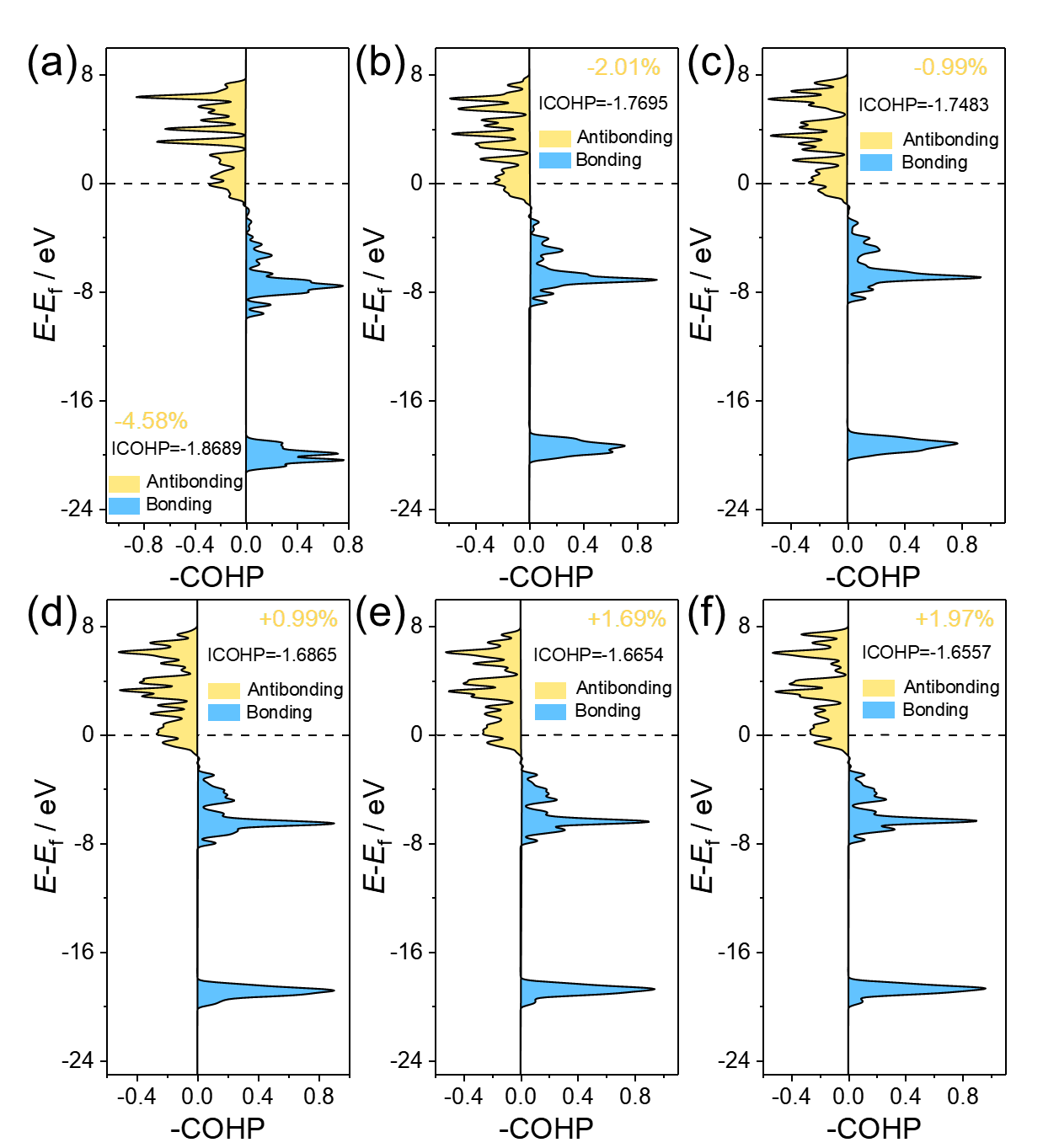


**Supplementary Fig. 25 | Calculated COHP of strained RuO_2_ (110).** (a) RuO_2_ (110) subjected to 4.58% compression strain, (b) RuO_2_ (110) subjected to 2.01% compression strain, (c) RuO_2_ (110) subjected to 0.99% compression strain, (d) RuO_2_ (110) subjected to 0.99% tensile strain, (d) RuO_2_ (110) subjected to 1.69% tensile strain, (e) RuO_2_ (110) subjected to 1.97% tensile strain.


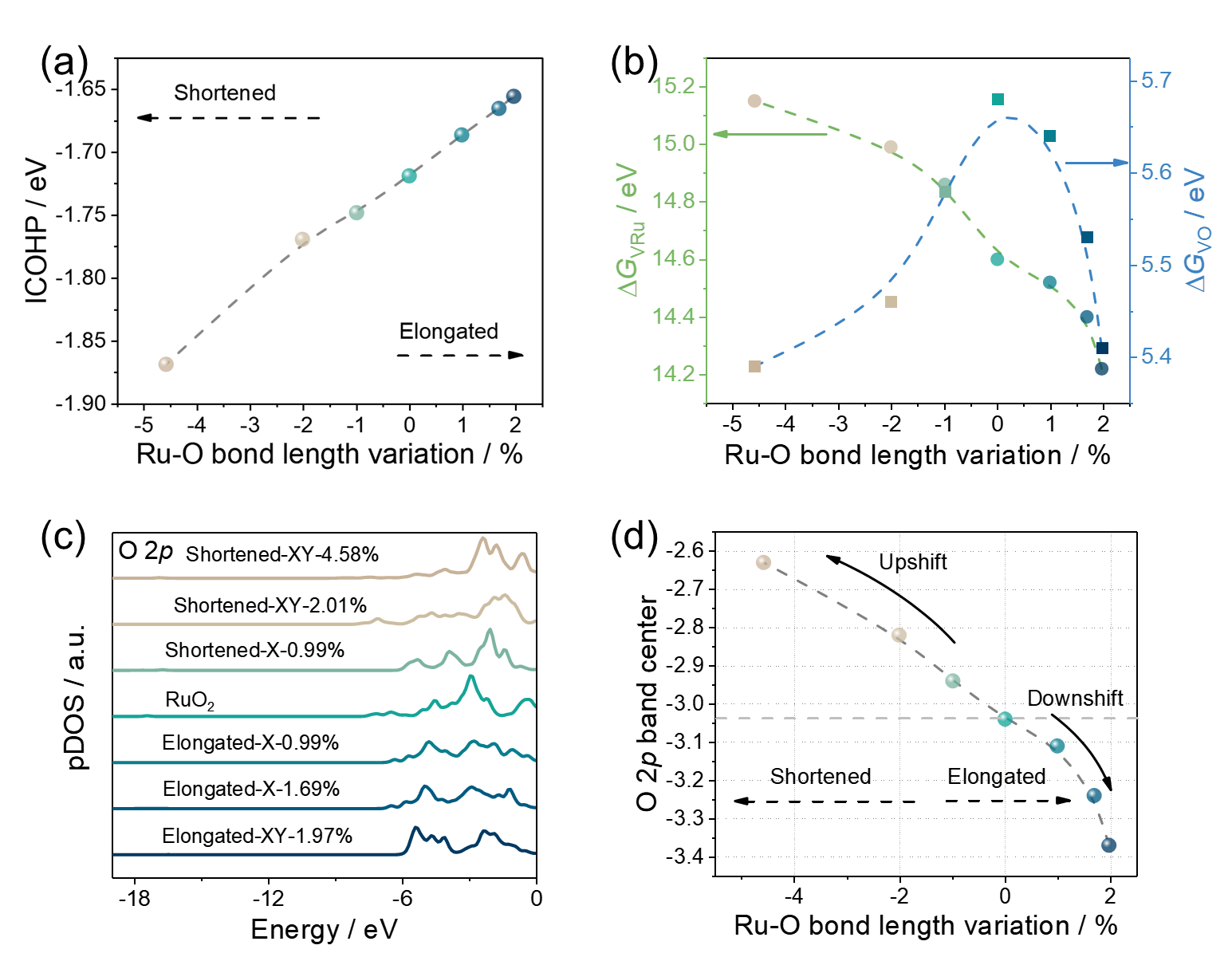


**Supplementary Fig. 26 | Ru-O bonding nature of strained RuO_2_ (110) on the scale of Ru-O bond length variation.** (a) ICOHP, (b) formation energy of oxygen vacancy and Ru vacancy (c, d) O 2*p* band center of strained RuO_2_ (110) on the scale of Ru-O bond length variation.


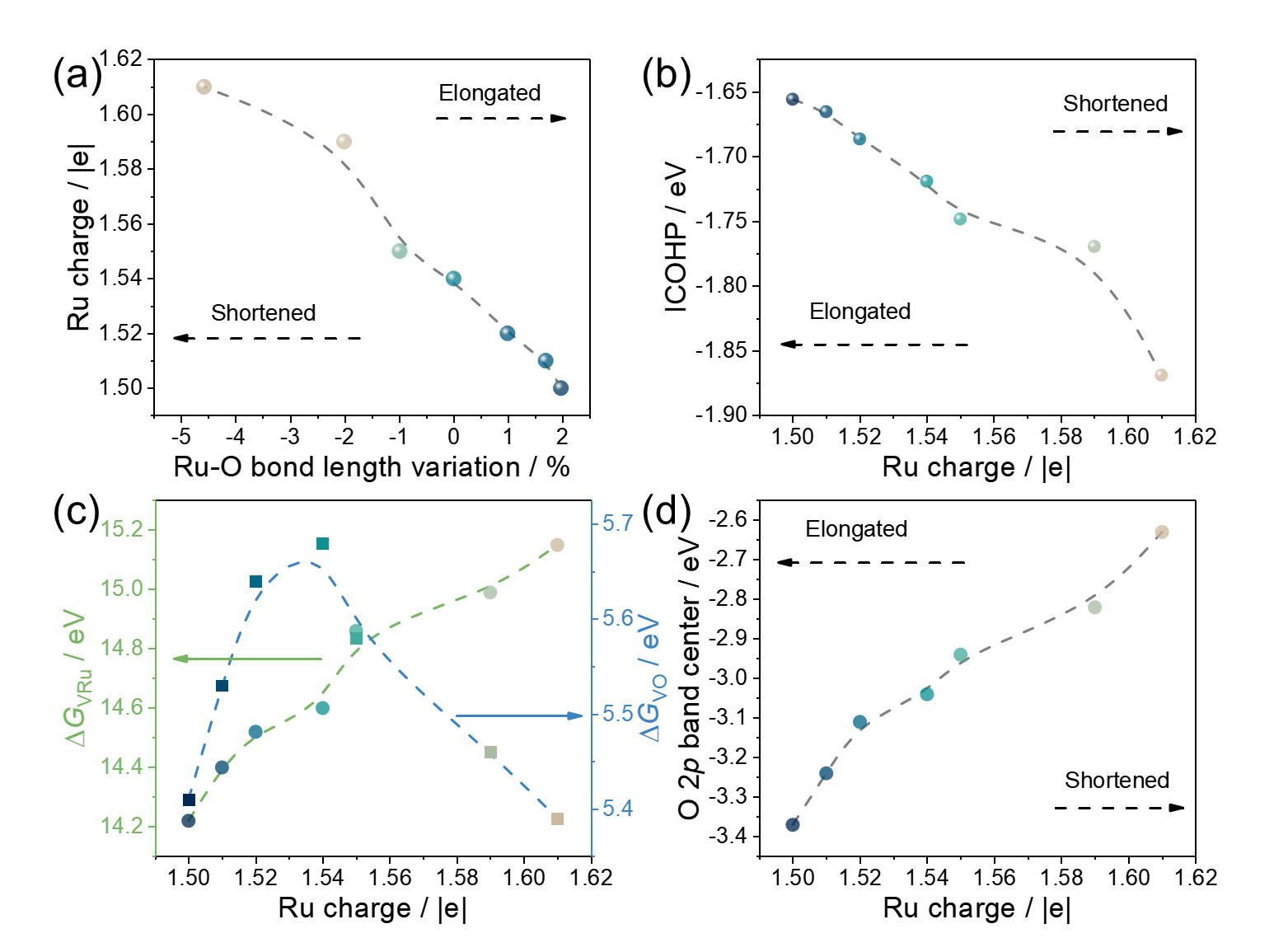


**Supplementary Fig. 27 | Ru-O bonding nature of strained RuO_2_ (110) on the scale of Ru charge.** (a) Ru charge of strained RuO_2_ (110) on the scale of Ru-O bond length variation; (b) ICOHP, (c) formation energy of oxygen vacancy and Ru vacancy (d) O 2*p* band center of strained RuO_2_ (110) on the scale of Ru charge.


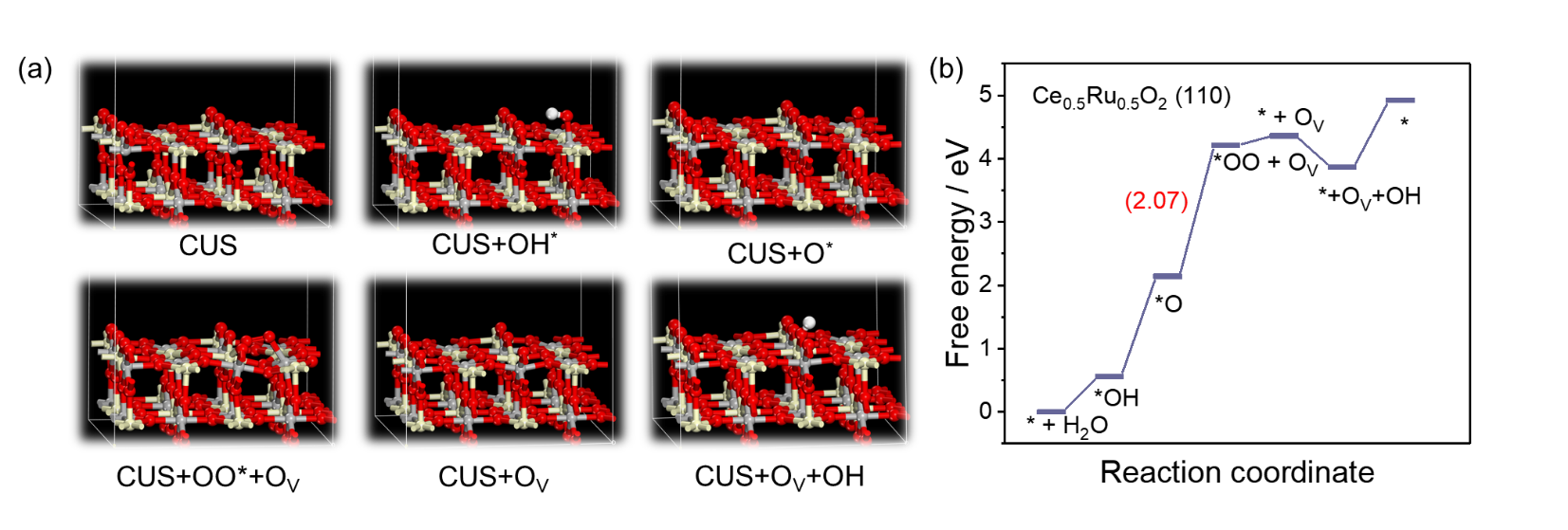


**Supplementary Fig. 28 | The free energy diagram of the LOM path on Ru site for Ce_0.5_Ru_0.5_O_2_.** (a) Structures of the key intermediates on LOM path, (b) Gibbs free energy diagram at 0 V based on LOM.


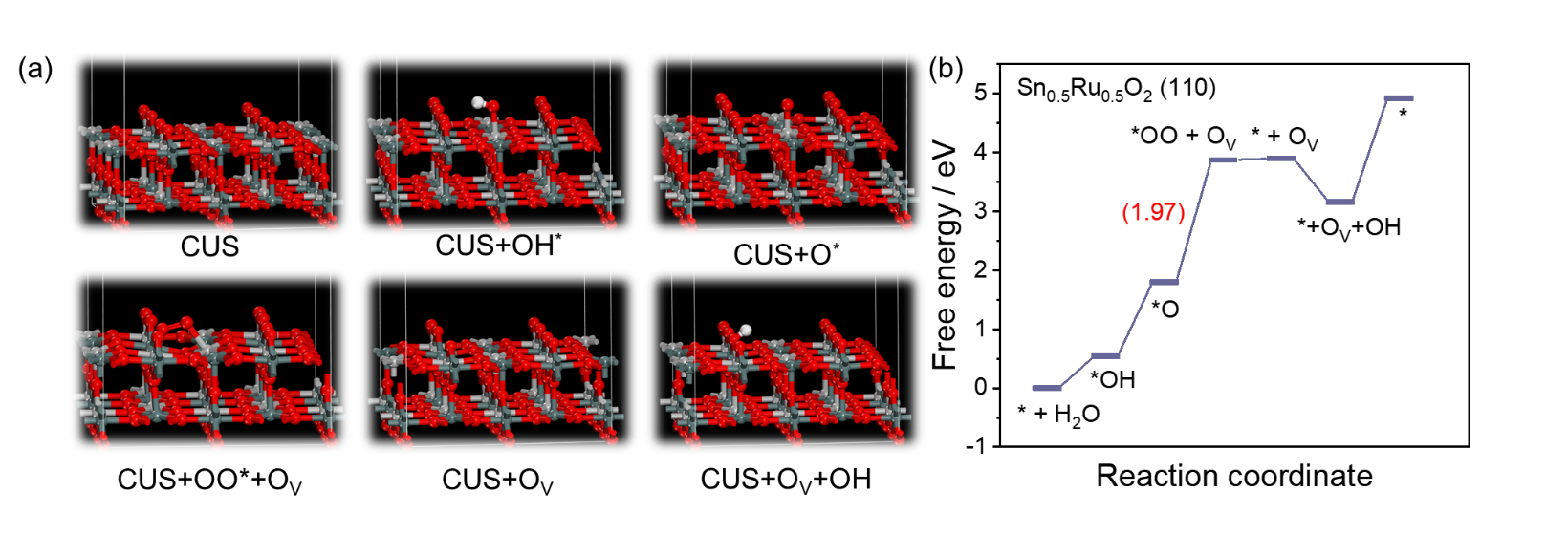


**Supplementary Fig. 29 | The free energy diagram of the LOM path on Ru site for Sn_0.5_Ru_0.5_O_2_.** (a) Structures of the key intermediates on LOM path, (b) Gibbs free energy diagram at 0 V based on LOM.


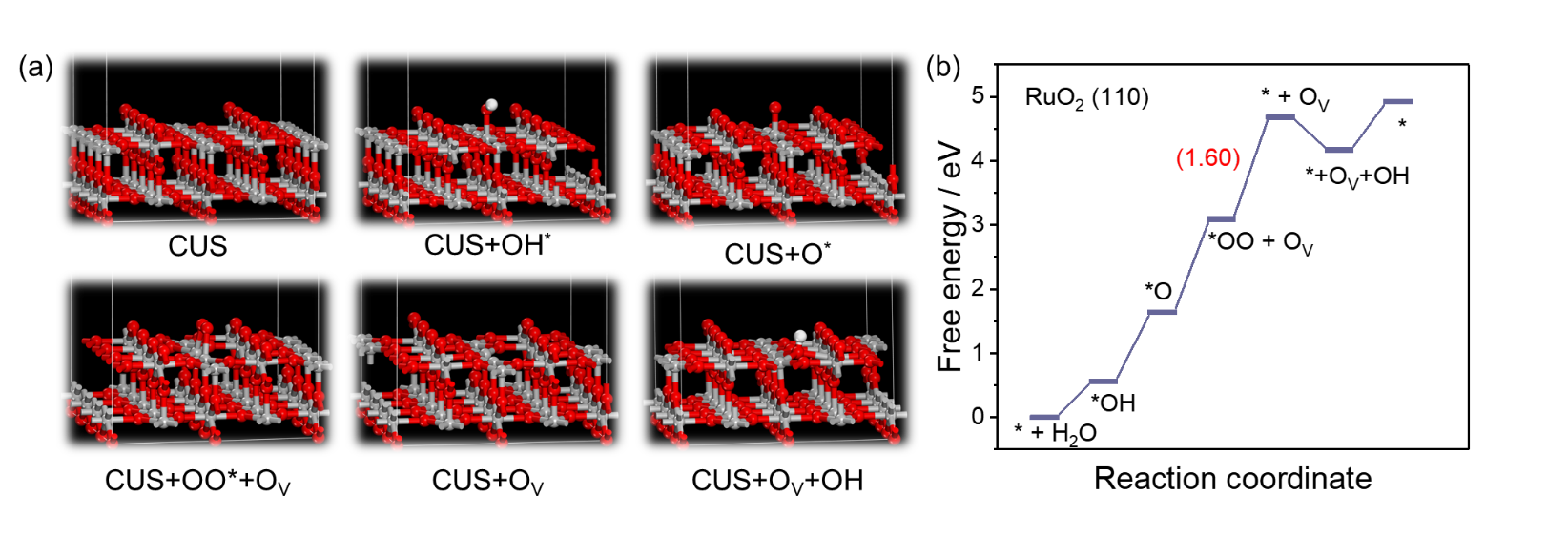


**Supplementary Fig. 30 | The free energy diagram of the LOM path on Ru site for RuO_2_.** (a) Structures of the key intermediates on LOM path, (b) Gibbs free energy diagram at 0 V based on LOM.


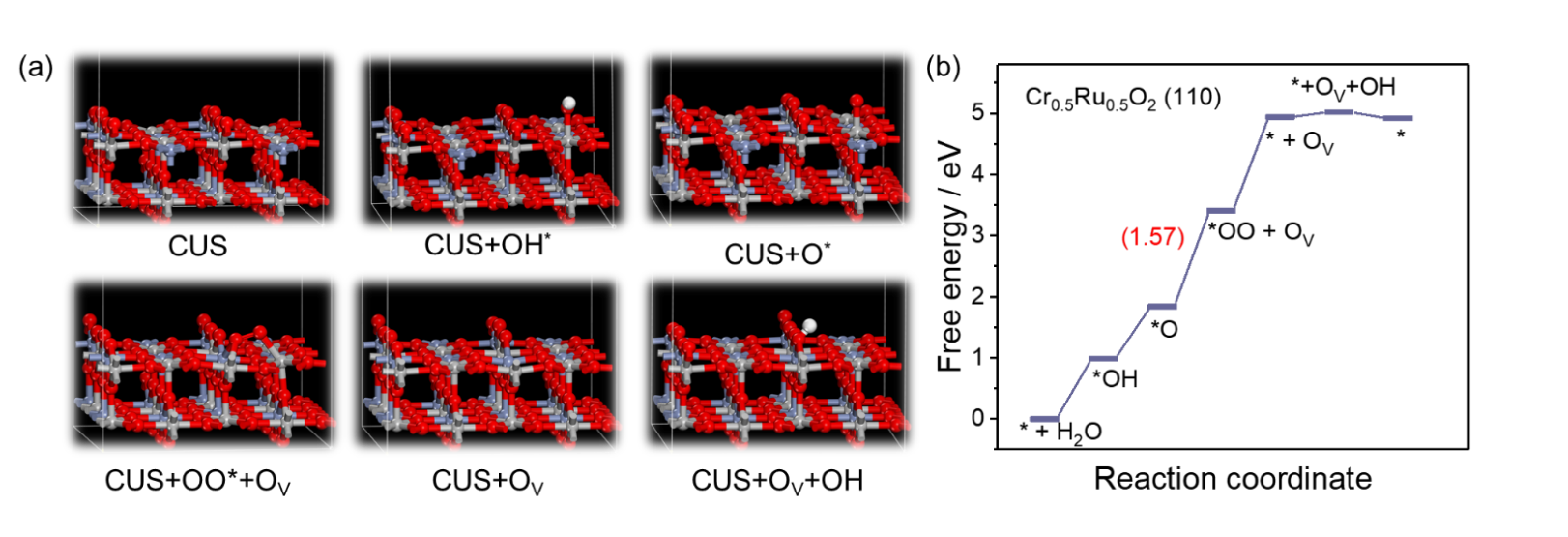


**Supplementary Fig. 31 | The free energy diagram of the LOM path on Ru site for Cr_0.5_Ru_0.5_O_2_.** (a) Structures of the key intermediates on LOM path, (b) Gibbs free energy diagram at 0 V based on LOM.


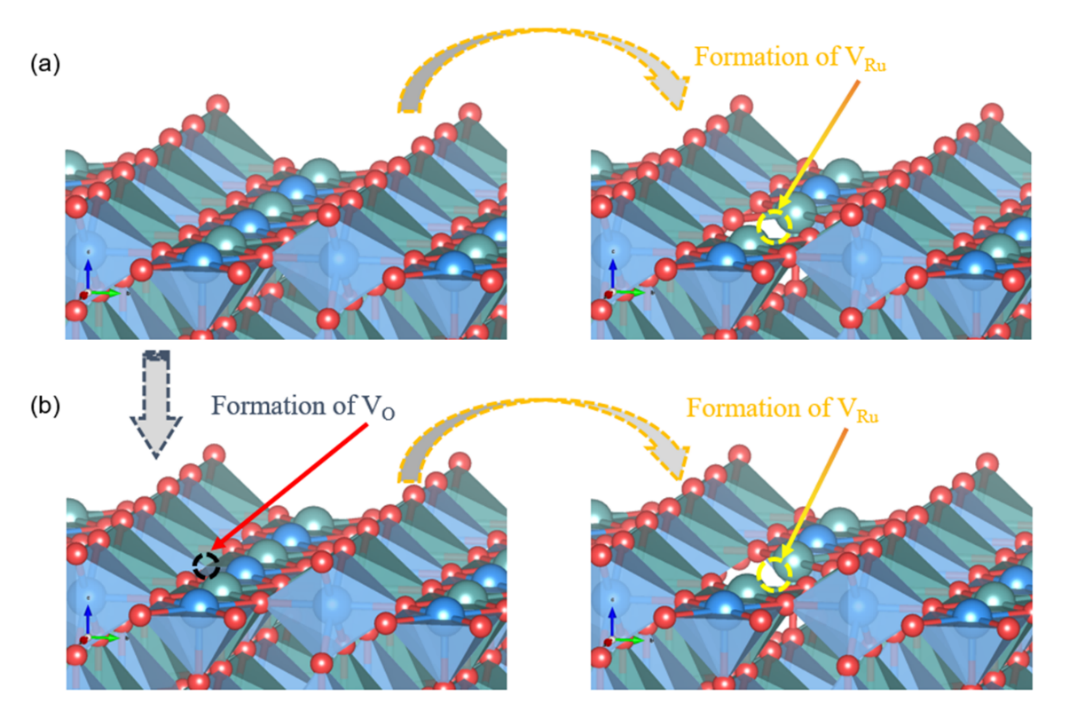


**Supplementary Fig. 32 | Formation of Ru vacancy for M_0.5_Ru_0.5_O_2_.** (a) Direct Ru vacancy formation on the surface of Ce_0.5_Ru_0.5_O_2_ and Sn_0.5_Ru_0.5_O_2_. (b) The process of Ru vacancy formation for RuO_2_ and Cr_0.5_Ru_0.5_O_2_ with oxygen vacancy generated considering the easy participation of lattice oxygen in OER.

**Supplementary Note 4 | DFT calculation on the defect structures of M_0.5_Ru_0.5_O_2_.**

Since the Ru and M vacancies formed during acidic OER may lead to deviation from the theoretical computational results on non-defective model, DFT calculations were thus conducted on the defective structure with the Ru and M vacancies considered (denoted as M_0.5_Ru_0.5_O_2_-Ru def and M_0.5_Ru_0.5_O_2_-M def hereafter). It is worth noting that for M_0.5_Ru_0.5_O_2_ (M = Ce, Sn and Cr) with M and Ru vacancies, the nearest Ru sites (Supplementary Fig. 33a, b) are selected for calculation since the influence of vacancies can be maximized. When considering the Ru vacancy in RuO_2_, the nearest Ru sites (Supplementary Fig. 33c) resemble that in M_0.5_Ru_0.5_O_2_-M def (M = Ce, Sn and Cr). Thus, the calculational results of RuO_2_-Ru def were compared with those of M_0.5_Ru_0.5_O_2_-M def instead of M_0.5_Ru_0.5_O_2_-Ru def, due to the high similarity in structure of the two. The results are as follows.

The charge of Ru in M_0.5_Ru_0.5_O_2_-M def and M_0.5_Ru_0.5_O_2_-Ru def was first calculated. Although the vacancy formation (especially M defect) leads to variation in the value of Ru charge, the intrinsic trend that increased electronegativity of M^4+^ from 1.608 (Ce^4+^) to 1.861 (Cr^4+^) lead to increased Ru charge still remains (Supplementary Fig. 34). Specifically, the charge of the nearest Ru sites increases significantly after the formation of M vacancy, which may derive from the fact that Ru-O bonding interaction is affected by the fracture of M-O bond (detailed discussion below). For those with Ru vacancy, since neither Ru nor the Ru coordinated O atoms directly interact with the vacancies (Supplementary Fig. 33b), minimal changes in Ru charge are observed.

We then move on to access the Ru-O bonding feature in the defective structure via calculating the COHP and projected DOS. As shown in Supplementary Fig. 35, with the inclusion of M defects, the ICOHP between Ru and O show a drastic negative shift (i.e. -1.280 eV to -1.519 eV for Ce_0.5_Ru_0.5_O_2_, -1.733 eV to -1.927 eV for Cr_0.5_Ru_0.5_O_2_, Supplementary Fig. 36), implying the increased Ru-O bond covalency, which is consistent with the higher Ru charge (Supplementary Fig. 34). However, similar to that of the Ru charge, the ICOHP of M_0.5_Ru_0.5_O_2_-Ru def has only a small deviation compared to the perfect structure (Supplementary Fig. 36), implying the maintenance of the Ru-O bonding feature.

We further turned to investigate the local fine electronic structure of Ru and O around Fermi level to figure out the Ru-O orbital interaction (Supplementary Fig. 37). Both the M and Ru defects lead to an upshift in Ru (*d*) and O (*p*) band center (M defects more so than Ru defects, Supplementary Fig. 38a, b), which is consistent with the increased Ru charge and higher Ru-O bond covalency. Thus, it is foreseeable that the formation of vacancies will lead to enhanced adsorption of oxygen species and easier formation of oxygen vacancies. Besides, we find the M defect significantly increases the O (*p*) band contribution around Fermi level (i.e. 2.2 % for Ce_0.5_Ru_0.5_O_2_ to 17.71 % for Ce_0.5_Ru_0.5_O_2_-Ce def, 25.17 for Cr_0.5_Ru_0.5_O_2_ to 31.74 % for Cr_0.5_Ru_0.5_O_2_-Cr def, Supplementary Fig. 38c), suggesting the ease in oxygen vacancy formation, which is in line with the down-shifted ICOHP and up-shifted O *p* band center. For M_0.5_Ru_0.5_O_2_-Ru def, however, no significant change are observed compared to that of perfect structure.

To verify whether Ru charge can be utilized as the intuitive descriptor, the ICOHP (Supplementary Fig. 39a), Ru *d* band center (Supplementary Fig. 39b), O *p* band center (Supplementary Fig. 39c), the O (*p*) contribution around Fermi level (Supplementary Fig. 39d) and Δ*G*_VO_ (Supplementary Fig. 39e) are plotted as a function of Ru charge. As the results presented, we find these electronic structure parameters take the same trending with the change in Ru charge. Specifically, with Ru charge increases from 1.49 |e| to 1.59 |e|, the ICOHP down-shifted from -1.280 eV to -1.927 eV, the Ru *d* band center up-shifted from -4.16 eV to -3.1 eV, the O *p* band center up-shifted from -3.57 eV to -2.72 eV, and the O *p* band contribution around the Fermi level increased from 2.2 % to 31.74 %, accompanied with decrease in Δ*G*_VO_ from 7.2 eV to 4.78 eV. These results in combine imply that with M and Ru vacancy considered, the Ru charge can still be utilized as the intuitive descriptor to reflect the variation in Ru-O bonding nature.

As verification, the theoretical activity of OER under AEM and LOM pathway on the defect structure is calculated (Supplementary Fig. 40), with the results summarized in Supplementary Fig. 41. Obviously, both the theoretical overpotential obtained under AEM and LOM share the same trend as that of the perfect structure (Fig. 2c and f). Specifically, the AEM pathway activities of all the defective and non-defective structures are revealed to follow a volcanic-like trend as a function of Ru charge (Supplementary Fig. 41a), giving a minimum value of Ru charge at~1.51 |e|, indicating that Ru sites charge can be successfully used to indicate the AEM reaction activity. With the increased Ru charge (1.49 |e| to 1.59 |e|), the theoretical overpotential of OER under LOM path gradually decreases from 1.026 eV to 0.250 eV (Supplementary Fig. 41b), which is attributable to the up-shifted O *p* band center and increased contribution of oxygen states around Fermi level. To here, whether or not vacancies are introduced, we confirm the Ru charge is able to serve as an intuitive descriptor to index the variation of OER catalytic activity under AEM and LOM.

A step further, the Δ*G*_VRu_ was calculated to investigate the stability. Although the Ru-O bond strength increases with increase in Ru charge, which should lead to the gradual increase in Δ*G*_VRu_, the formation of lattice oxygen vacancies for those under LOM (Supplementary Fig. 42) results in drastic decrease in the Δ*G*_VRu_. Thus, the calculated Δ*G*_VRu_ presented a volcanic-like trend as a function of Ru charge (Supplementary Fig. 43a), which also resembles that obtained on non-defective M_0.5_Ru_0.5_O_2_ (Supplementary Fig. 43b). Clearly, with the above-presented results, it is safe to claim that the Ru charge can be used to describe not only the major reaction path and OER activity, but also the structural stability, due to its successful reflection in both electronic features of Ru and overall Ru-O bonding interactions.


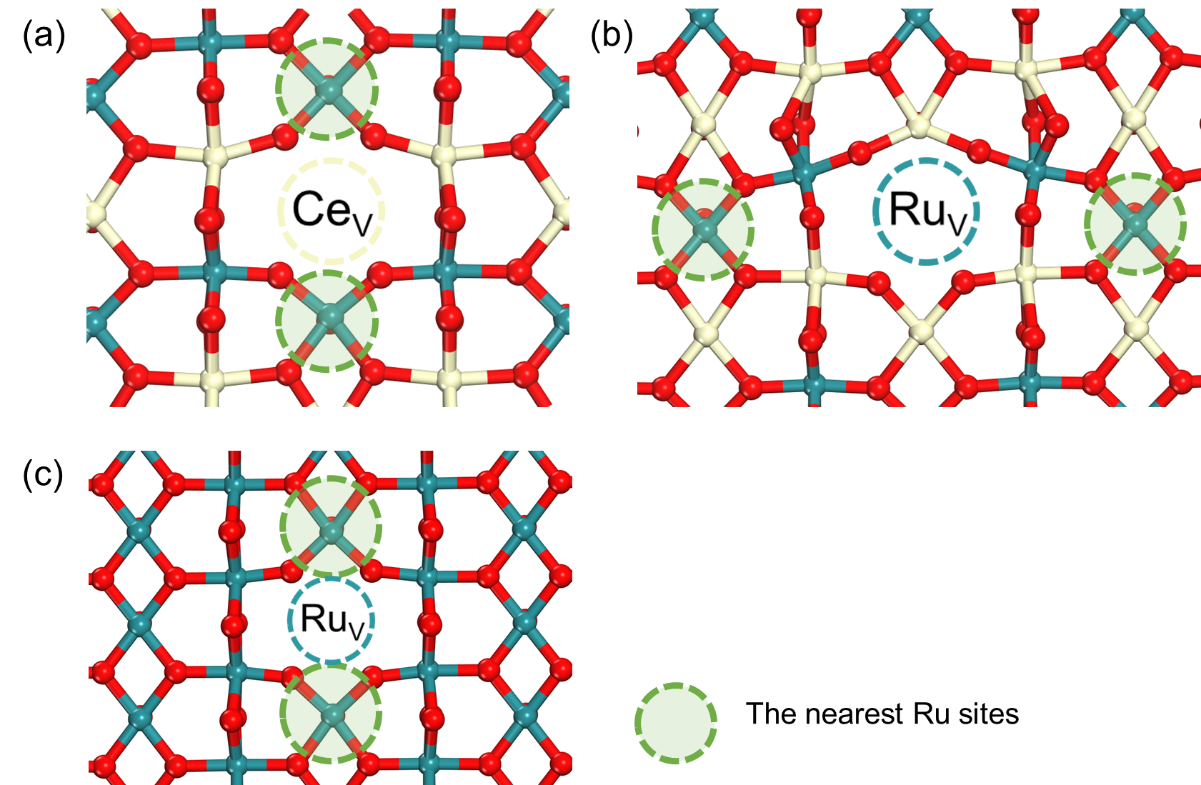


**Supplementary Fig. 33 | The nearest Ru sites on the defective structure.** (a) Nearest Ru sites with the formation of M defects (take Ce vacancy as an example here); (b) Nearest Ru sites with the formation of Ru defects (take Ru vacancy in Ce_0.5_Ru_0.5_O_2_ as an example here); (c) Nearest Ru sites with the formation of Ru defects in RuO_2_.


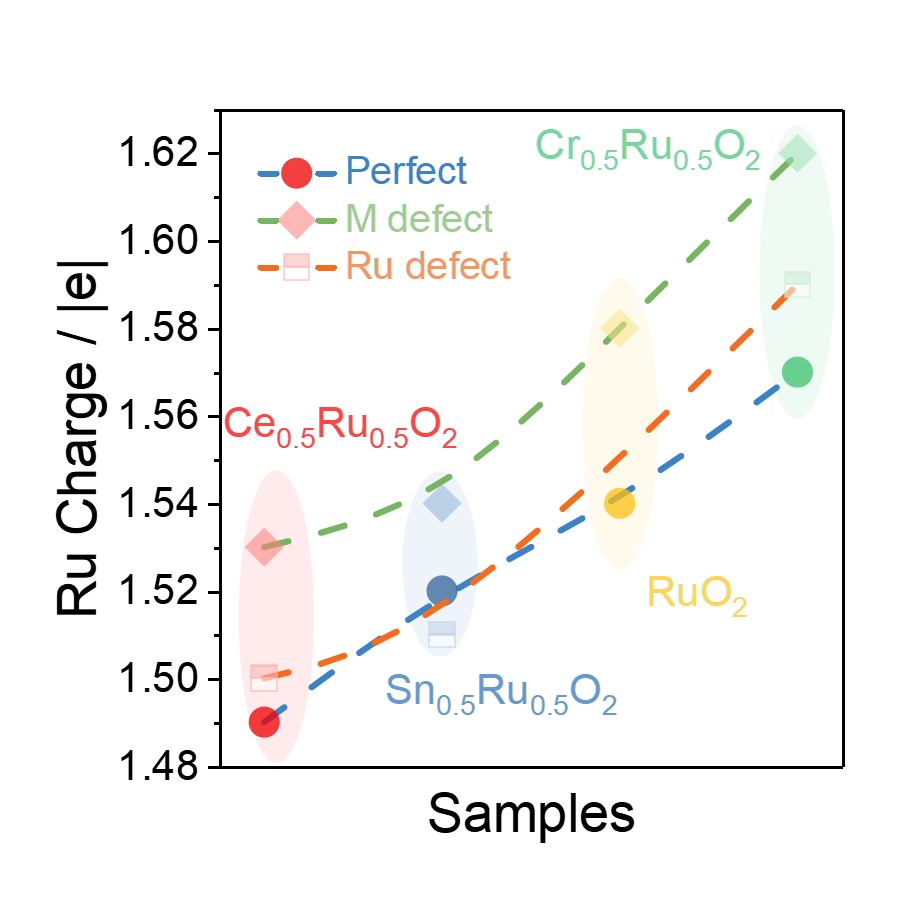


**Supplementary Fig. 34 | Ru charge of the defective and perfect M_0.5_Ru_0.5_O_2_.**


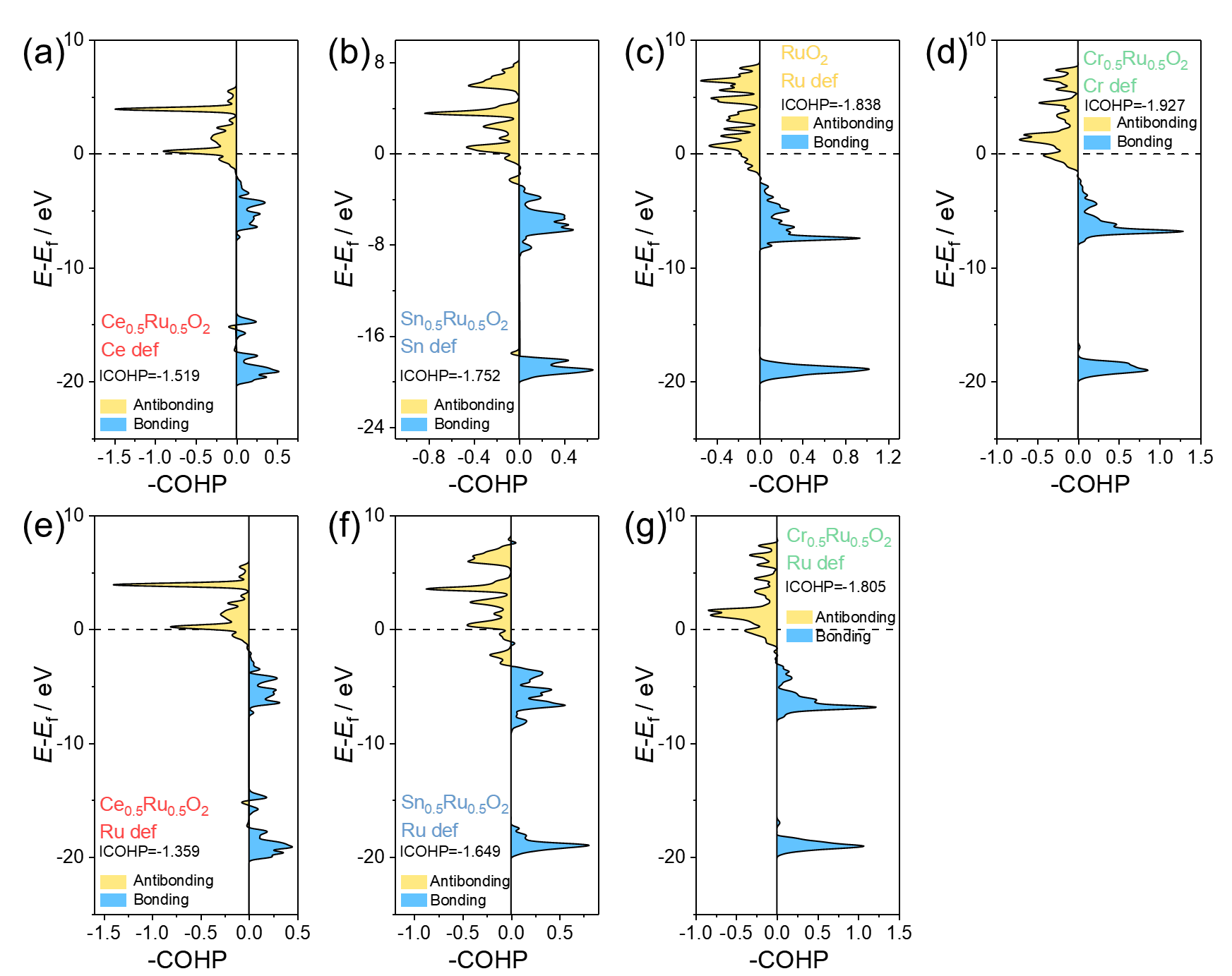


**Supplementary Fig. 35 | COHP of the defective M_0.5_Ru_0.5_O_2_.** (a) Ce_0.5_Ru_0.5_O_2_ with Ce defect, (b) Sn_0.5_Ru_0.5_O_2_ with Sn defect, (c) RuO_2_ with Ru defect, (d) Cr_0.5_Ru_0.5_O_2_ with Cr defect, (e) Ce_0.5_Ru_0.5_O_2_ with Ru defect, (f) Sn_0.5_Ru_0.5_O_2_ with Ru defect, (g) Cr_0.5_Ru_0.5_O_2_ with Ru defect.


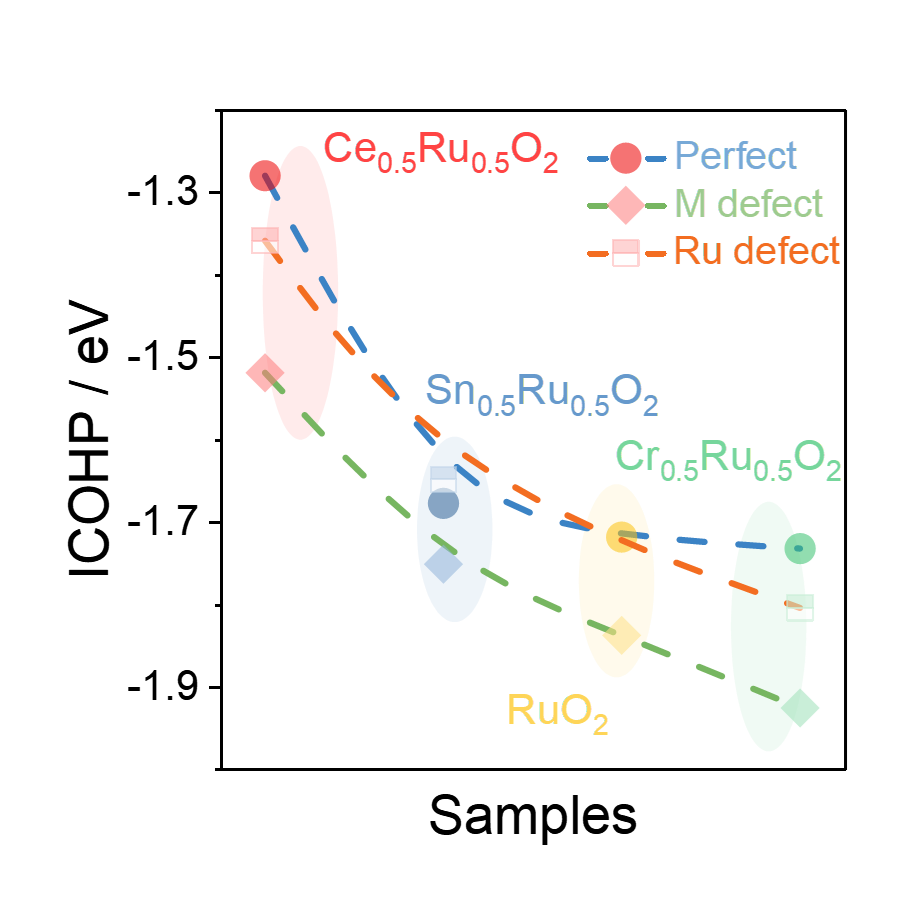


**Supplementary Fig. 36 | ICOHP of the defective and perfect M_0.5_Ru_0.5_O_2_.**


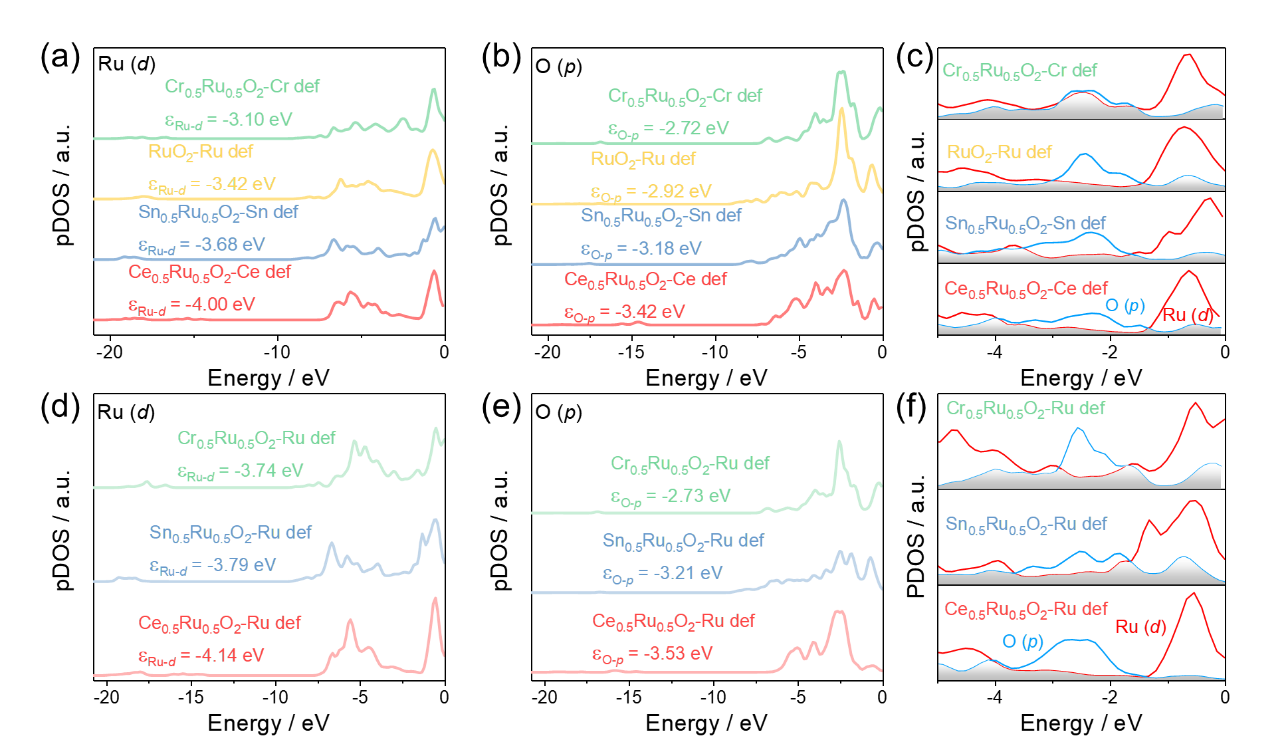


**Supplementary Fig. 37 | pDOS of defective and non-defective M_0.5_Ru_0.5_O_2_.** Ru *d* band (a, d), O *p* band (b, e) and Ru (*d*) – O (*p*) overlap structure of M_0.5_Ru_0.5_O_2_ with (a-c) M defects and (d-f) Ru defects.


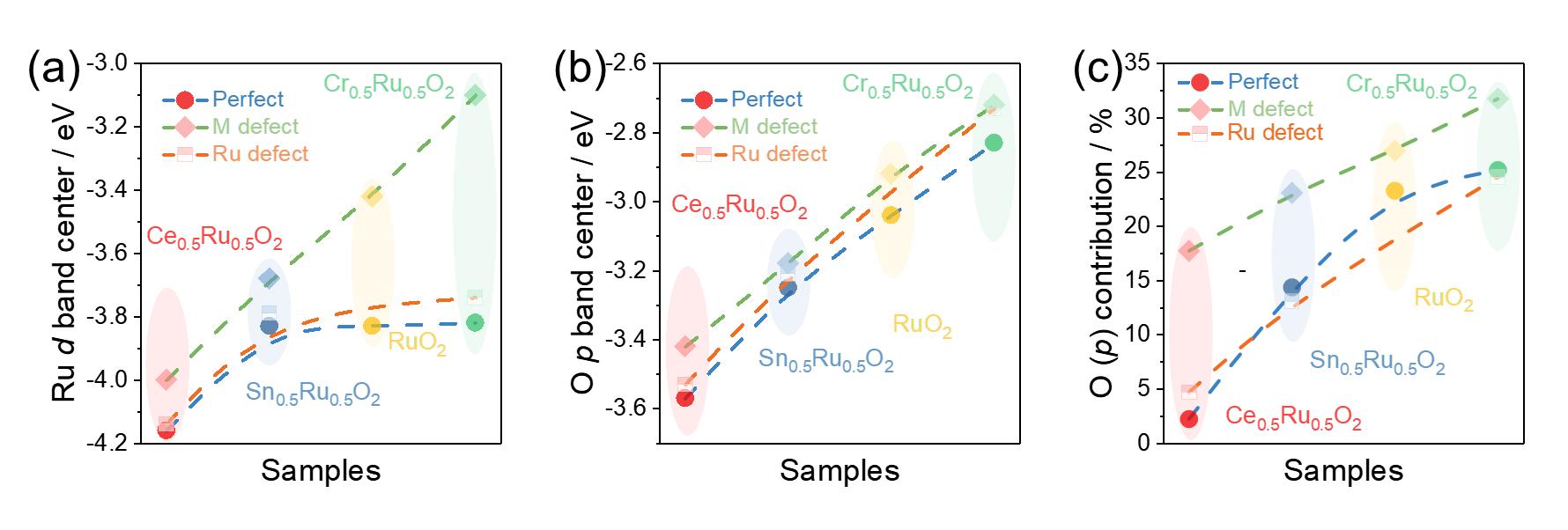


**Supplementary Fig. 38 | The local fine band structure of Ru *d* and O *p* for defective and perfect M_0.5_Ru_0.5_O_2_.** (a) Ru *d* band center, (b) O *p* band center, (c) O (*p*) contribution around the Fermi level.


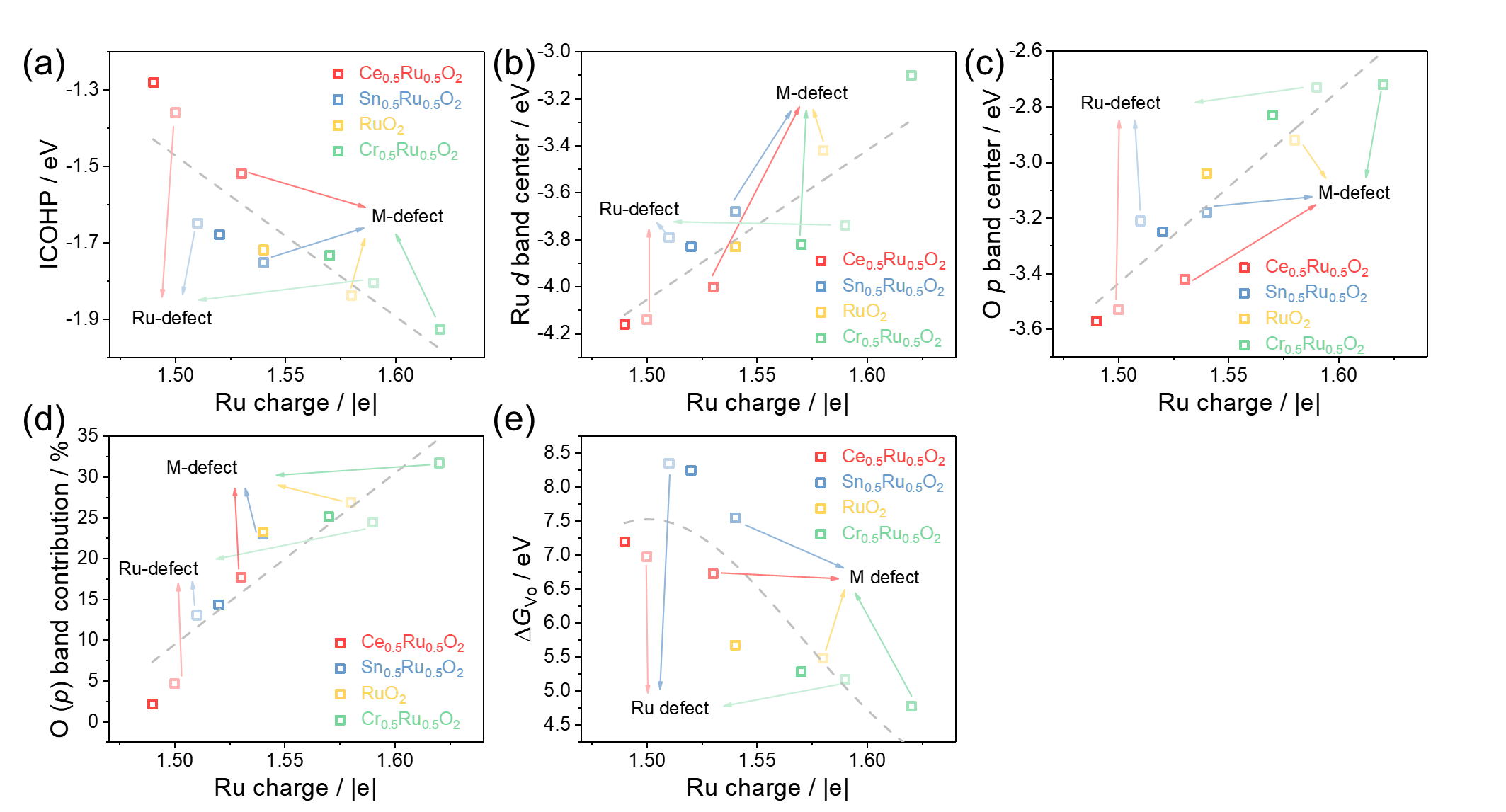


**Supplementary Fig. 39 | Ru-O bonding nature of defective and perfect M_0.5_Ru_0.5_O_2_ on the scale of Ru charge.** (a) ICOHP, (b) Ru *d* band center, (c) O *p* band center, (d) O (*p*) band contribution and (e) Δ*G*_VO_ of defective and perfect M_0.5_Ru_0.5_O_2_ on the scale of Ru charge. The fitted dash line is present to guide the eye.


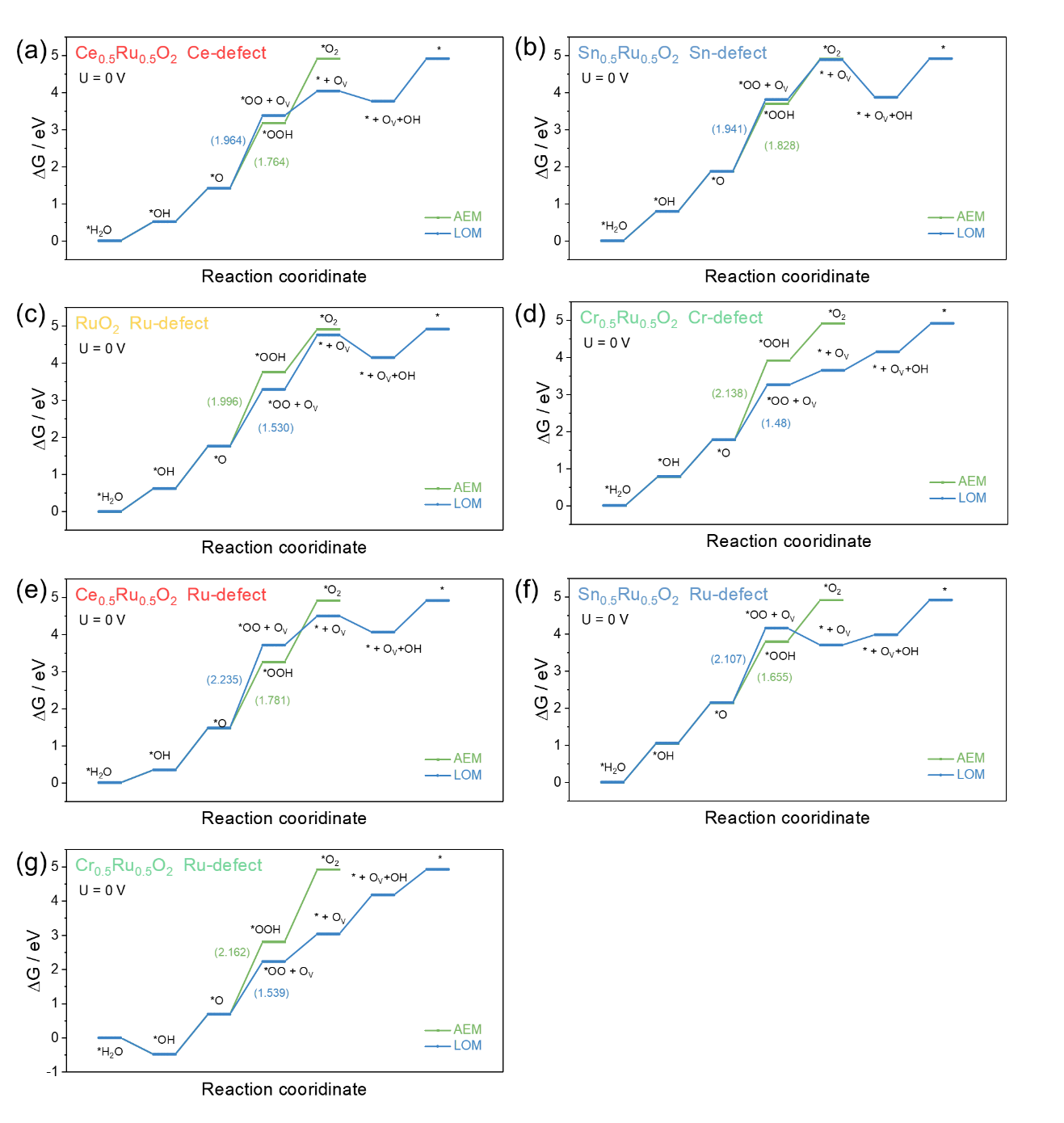


**Supplementary Fig. 40 |** The free energy diagram of AEM and LOM path on M_0.5_Ru_0.5_O_2_ with (a-d) M defects and (e-g) Ru defects.


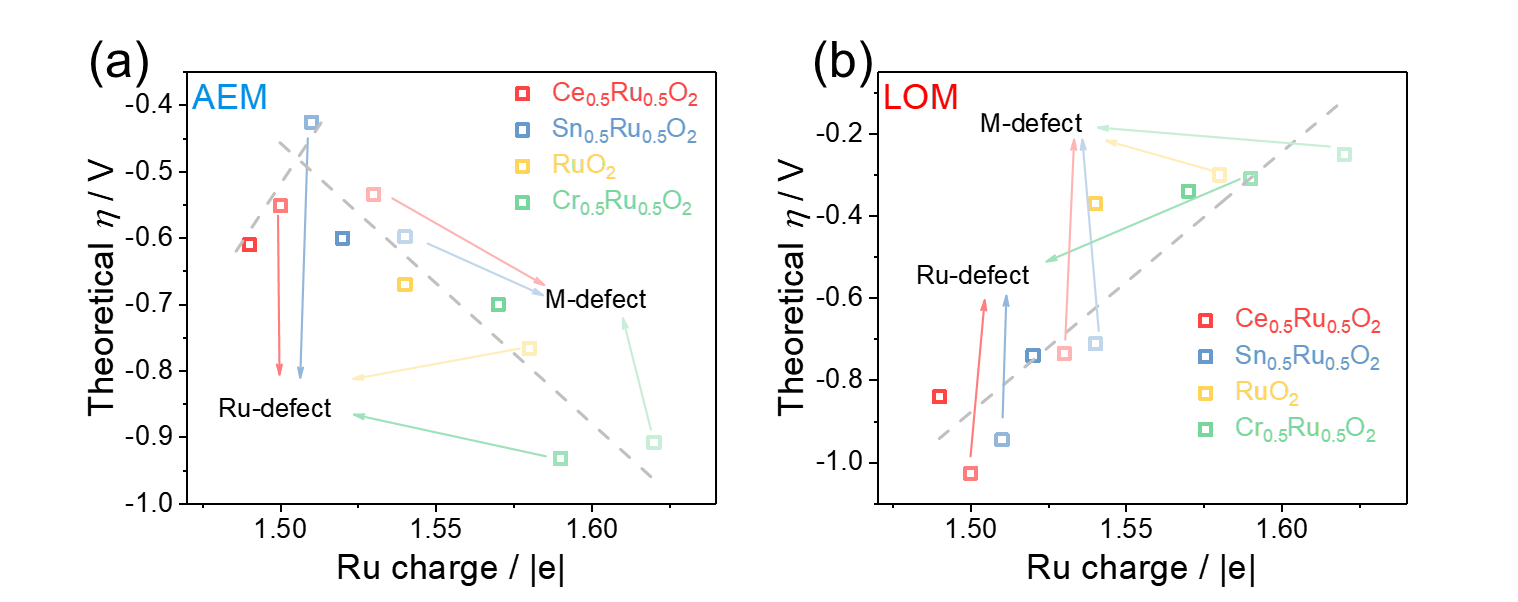


**Supplementary Fig. 41 |** (a) Theoretical overpotential volcano plot under AEM pathway for defective and perfect M_0.5_Ru_0.5_O_2_ as a function of the Ru charge; (b) Theoretical overpotential plot under LOM pathway for defective and perfect M_0.5_Ru_0.5_O_2_ as a function of the Ru charge.


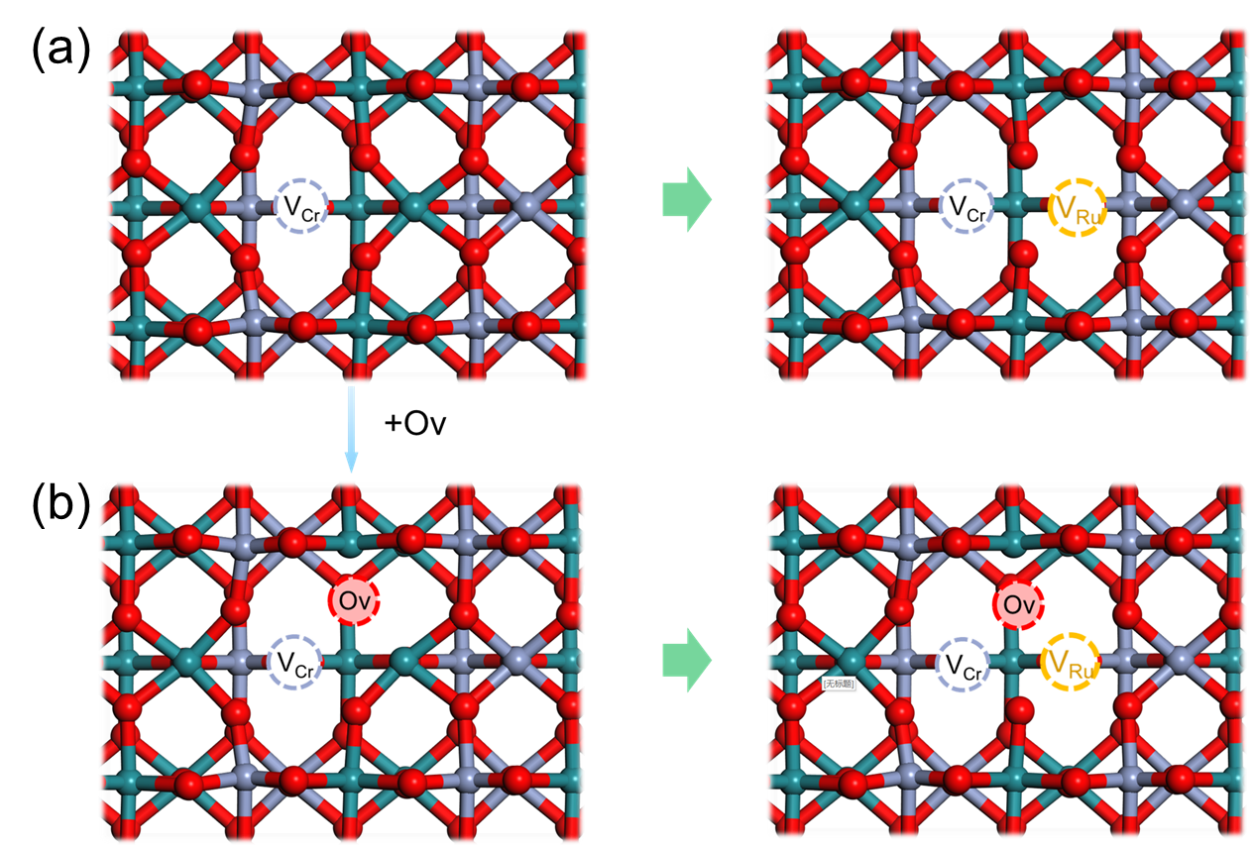


**Supplementary Fig. 42 | Formation of Ru vacancy for defective M_0.5_Ru_0.5_O_2_.** (a) Direct Ru vacancy formation on the surface of defective Ce_0.5_Ru_0.5_O_2_ and Sn_0.5_Ru_0.5_O_2_. (b) The process of Ru vacancy formation for defective RuO_2_ and Cr_0.5_Ru_0.5_O_2_ with oxygen vacancy generated considering the easy participation of lattice oxygen in OER


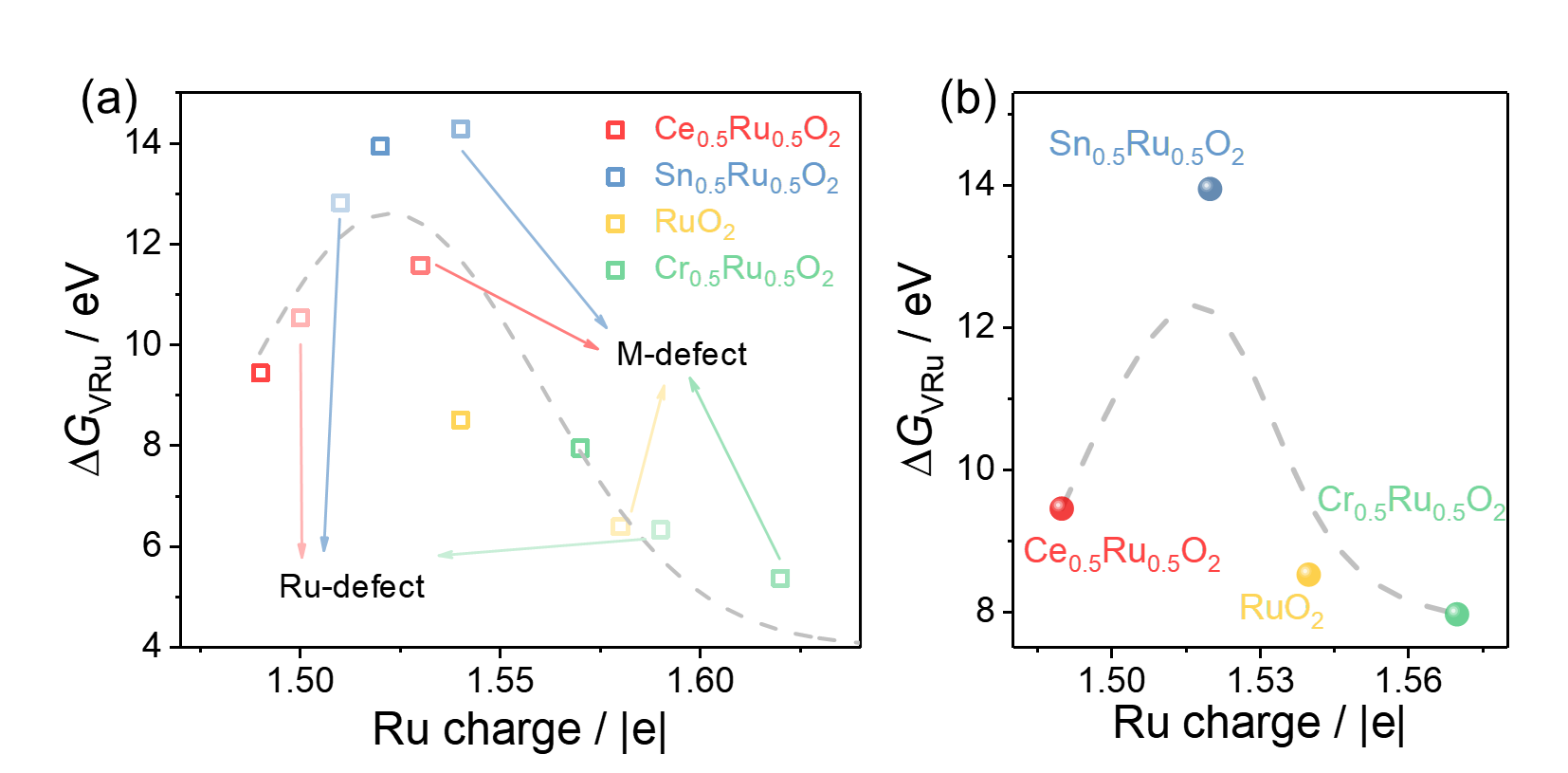


**Supplementary Fig. 43 | The formation energy of Ru vacancy for (a) defective and (b) non-defective M_0.5_Ru_0.5_O_2_ on the scale of Ru charge.**

**Supplementary Note 5 | Crossover between AEM and LOM pathways with the inclusion of M defects.**

With M vacancy considered, the LOM theoretical activity is improved for all samples (Supplementary Fig. 44a), i.e., 0.106 V decrease in overpotential for Ce_0.5_Ru_0.5_O_2_, 0.09 V decrease for Cr_0.5_Ru_0.5_O_2_. The boosted activity under LOM can be rationalized as the introduction of M vacancy leads to increased Ru-O bond covalency, higher O *p* band center, as well as higher oxygen states contribution near Fermi level, which thereby leads to decreased Δ*G*_VO_ and thus higher activity under LOM (Supplementary Fig. 45a-d). For RuO_2_-Ru def and Cr_0.5_Ru_0.5_O_2_-Cr def, the AEM reaction activity decreased drastically (0.096 V for RuO_2_ and 0.208 V for Cr_0.5_Ru_0.5_O_2_, Supplementary Fig. 44b), which may originate from the up-shifted Ru *d* band center (Supplementary Fig. 45e) and further strengthened oxygen binding energy. For Ce_0.5_Ru_0.5_O_2_-Ce def, improved AEM reaction activity is observed, which may also derive from the up-shifted Ru *d* band center and hence optimized binding energy compared to the too-weak adsorption on perfect Ce_0.5_Ru_0.5_O_2_. For Sn_0.5_Ru_0.5_O_2_, the AEM reaction activity is almost identical before and after considering Sn defect (0.600 V *vs.* 0.598 V).

While the crossover between AEM and LOM after inclusion of metal center vacancies is indeed consistent with the previous work from Alexandrov et al.^3^, we believe the crossover is originated from the variation in Ru charge. As shown in Supplementary Fig. 41, with M vacancy considered, the increased Ru charge will lead to lower overpotential on LOM. Under AEM, samples with initially low Ru charge (i.e. Ce_0.5_Ru_0.5_O_2_ and Sn_0.5_Ru_0.5_O_2_) show firstly activity increase and then decrease with increased Ru charge, while samples with initial high Ru charge (i.e. RuO_2_ and Cr_0.5_Ru_0.5_O_2_) show a direct decrease in activity with increased Ru charge. Thus, it is further verified that Ru charge is able to serve as a unified scale to describe the performance in both AEM and LOM pathways.


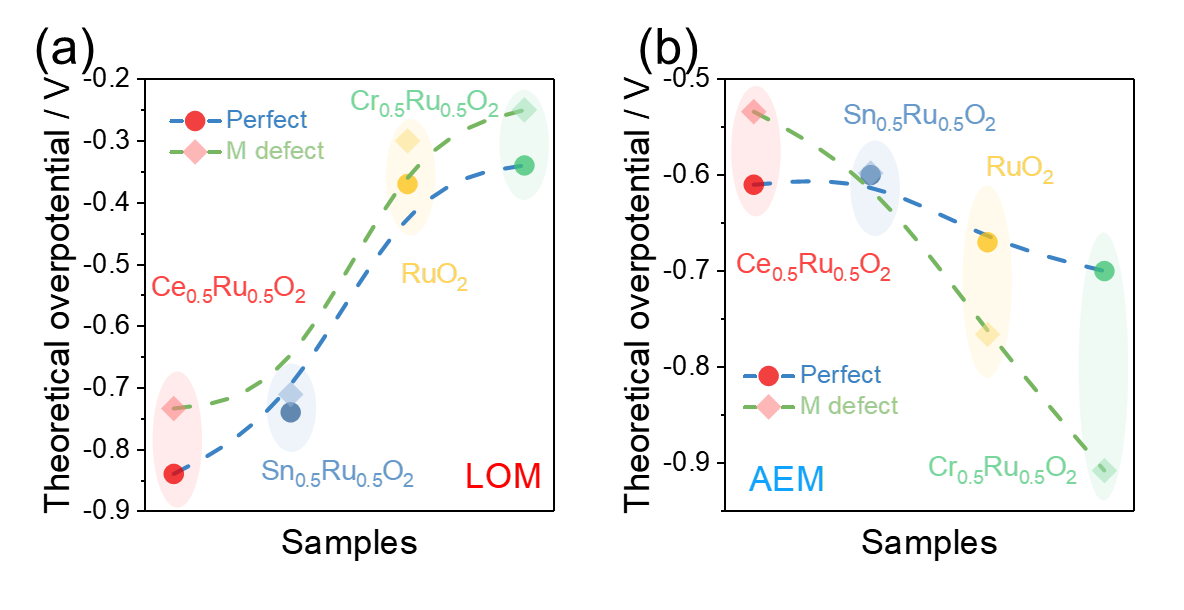


**Supplementary Fig. 44 |** Theoretical overpotential volcano plot under (a) LOM pathway and (b) AEM pathway on M_0.5_Ru_0.5_O_2_ and M_0.5_Ru_0.5_O_2_-M def as a function of the Ru charge.


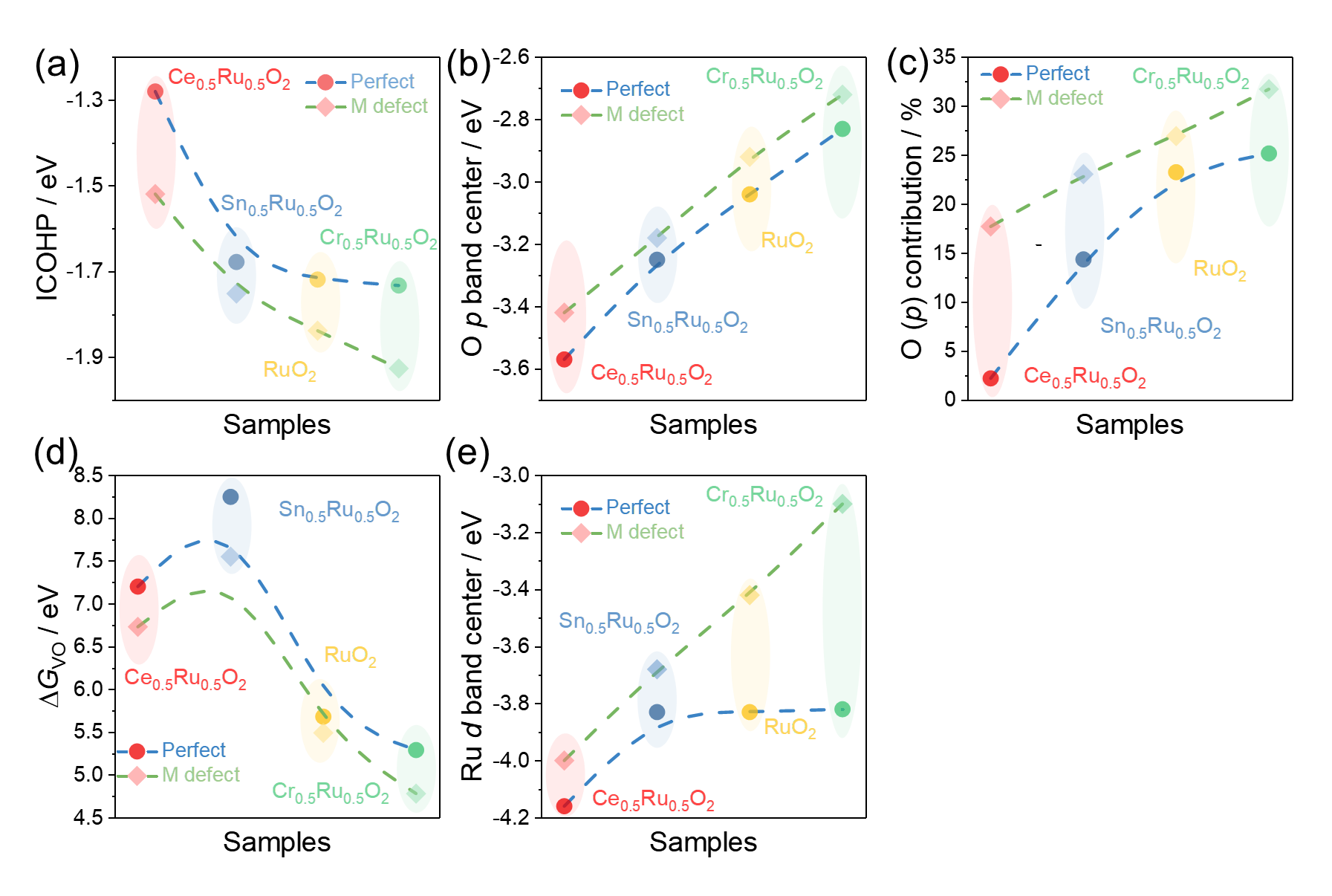


**Supplementary Fig. 45 | Ru-O bonding nature of M_0.5_Ru_0.5_O_2_ and M_0.5_Ru_0.5_O_2_-M def.** (a) ICOHP, (b) O *p* band center, (c) O *p* band contribution, (d) formation energy of oxygen vacancy and (e) Ru *d* band center of M_0.5_Ru_0.5_O_2_ and M_0.5_Ru_0.5_O_2_-M def.


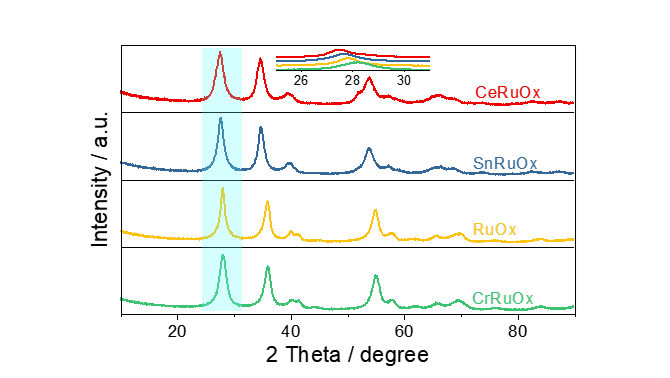


**Supplementary Fig. 46 | XRD patterns of MRuO_x_.** All the diffraction peak of MRuO**_x_** can be assign to a tetragonal phase (P42/MNM) same to the rutile RuO_2_, indicating the formation of solid solution. The inserted show the enlarged pattern at (110), demonstrating the changes in the cell parameter after M incorporation.


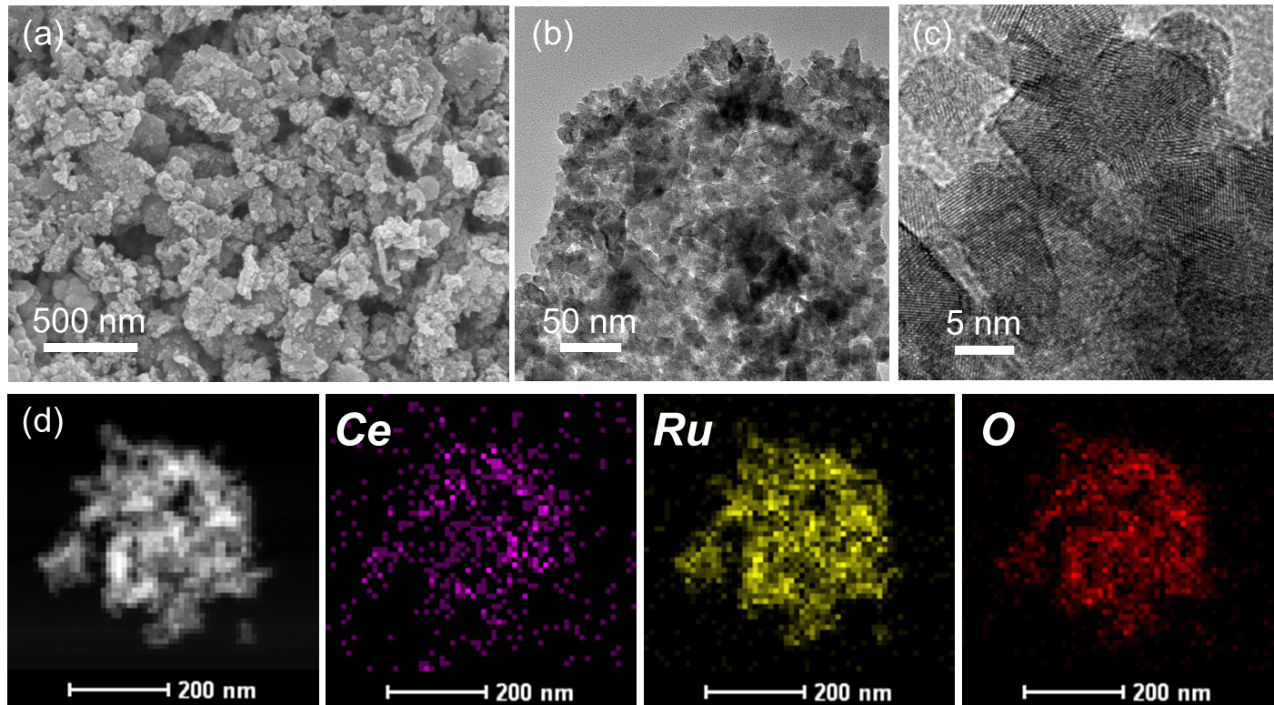


**Supplementary Fig. 47 | Morphology characterization on CeRuO_x_.** SEM image (a) and HRTEM images (b, c) implying that CeRuO**_x_** is composed of crystalline grains (10-20 nm), showing a crushing and cluttered irregular morphology. (d) EDX mapping of CeRuO**_x_** suggesting the uniform distribution of Ce, Ru, O.


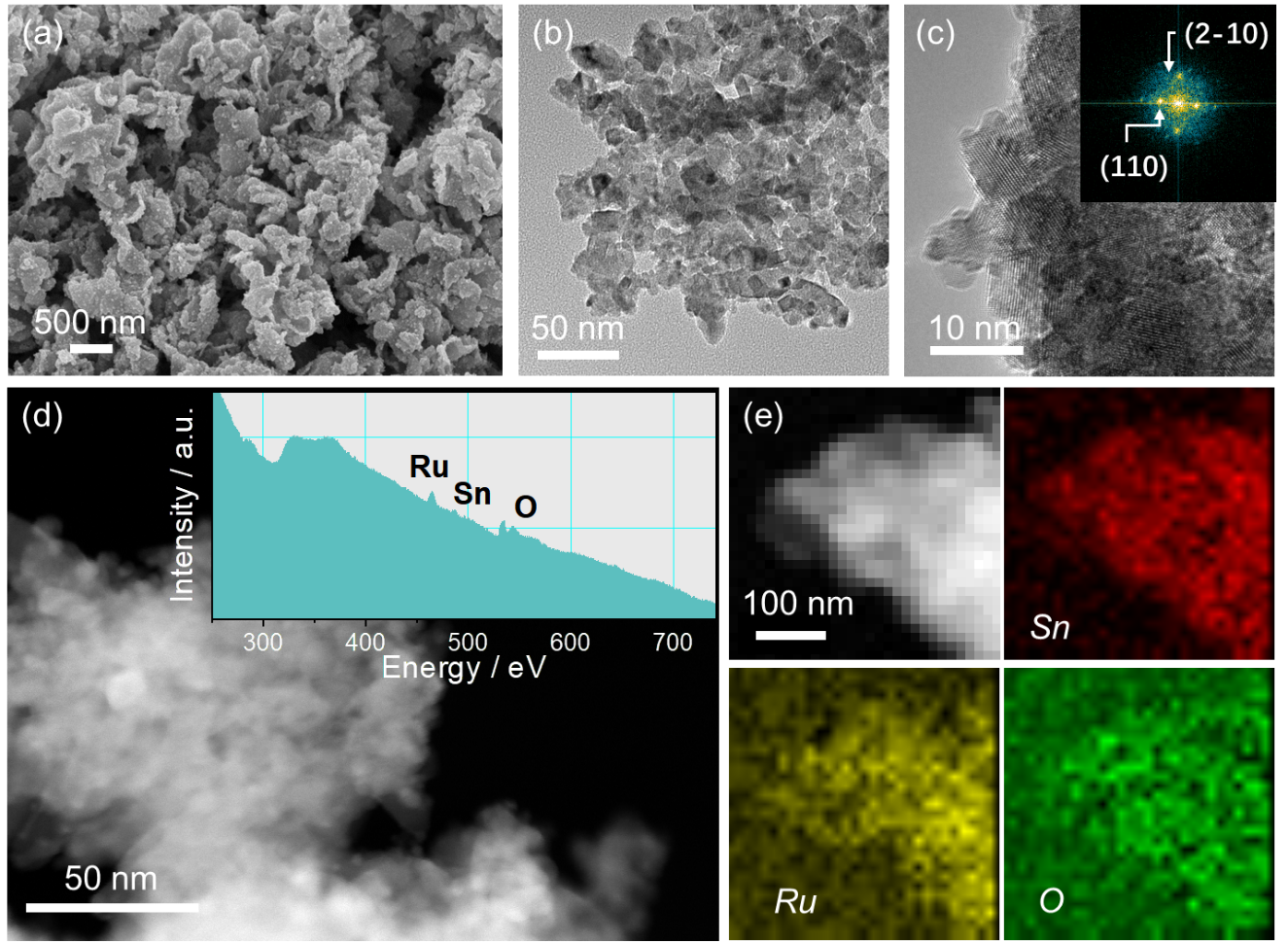


**Supplementary Fig. 48 | Morphology characterization on SnRuO_x_.** (a) SEM image, the curled sheet structure can be observed. (b, c) HRTEM images, the SnRuO**_x_** sheet are composed of highly crystalline grains with a size of about 10-20 nm. (d) HAADF-STEM image and the corresponding EELS spectra of SnRuO**_x_**. (e) EELS mapping of SnRuO**_x_** implying the uniform distribution of Sn, Ru and O in the sample.


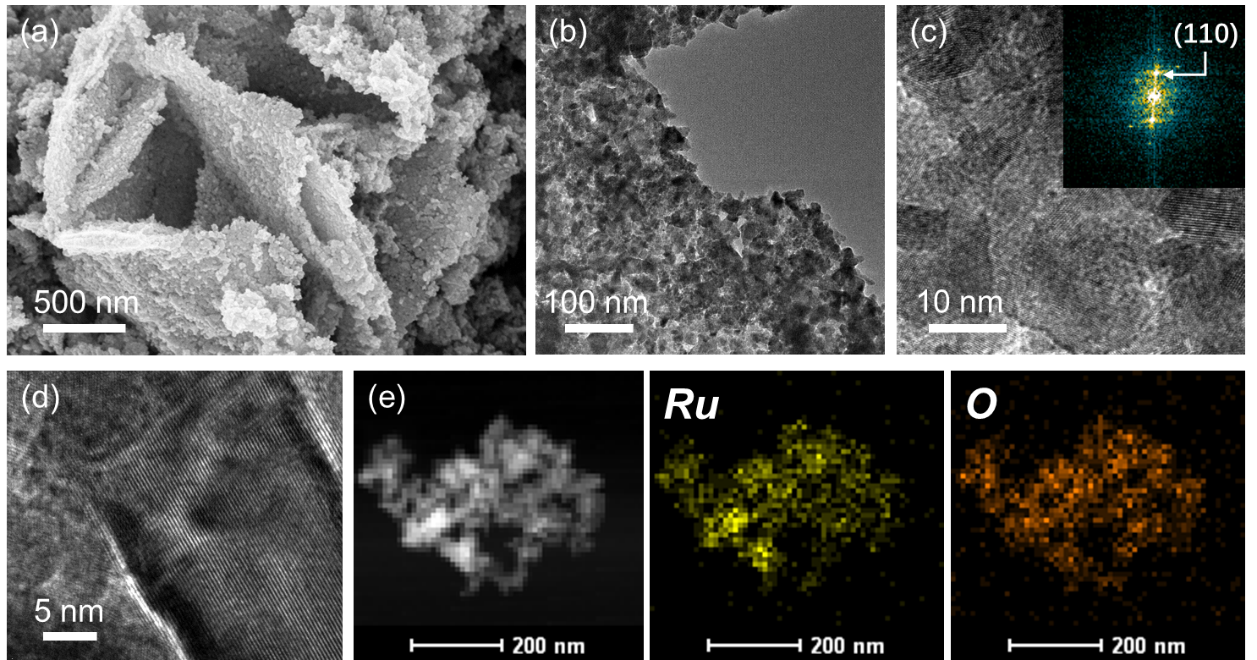


**Supplementary Fig. 49 | Morphology characterization on RuO_x_.** SEM (a) and HRTEM images (b-d) show that RuO**_x_** possess a sheet-like structure composed of highly crystalline grains with a particle size of about 20-40 nm. (e) EDX mapping demonstrating the uniform distribution of Ru and O in RuO**_x_**.


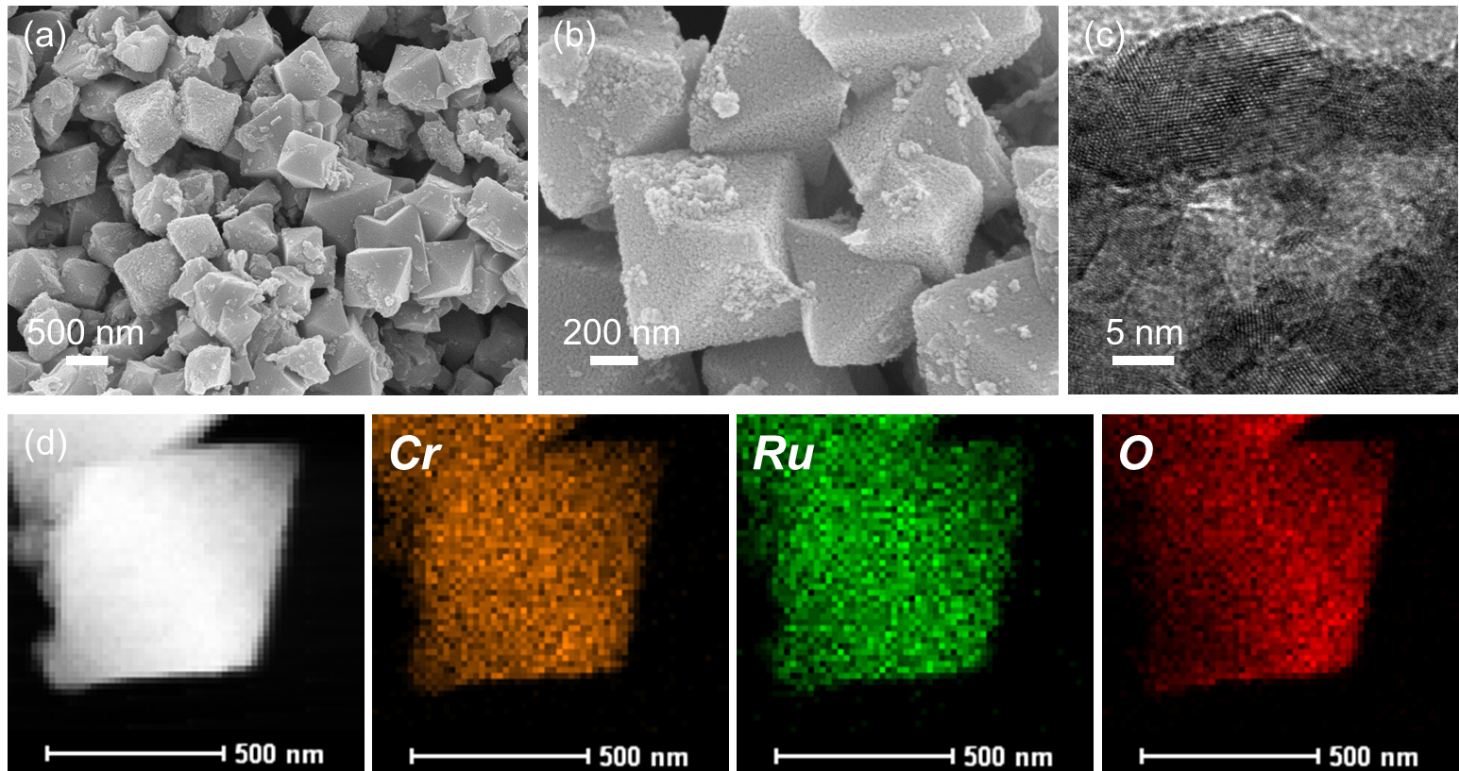


**Supplementary Fig. 50 | Morphology characterization on CrRuO_x_.** (a, b) SEM images and (c) TEM images reveled the CrRuO**_x_** with octahedral morphology is composed of crystalline grains with a small particle size. (d) EDX element mapping shows the uniform distribution of Cr, Ru and O in CrRuO**_x_** solid solution.


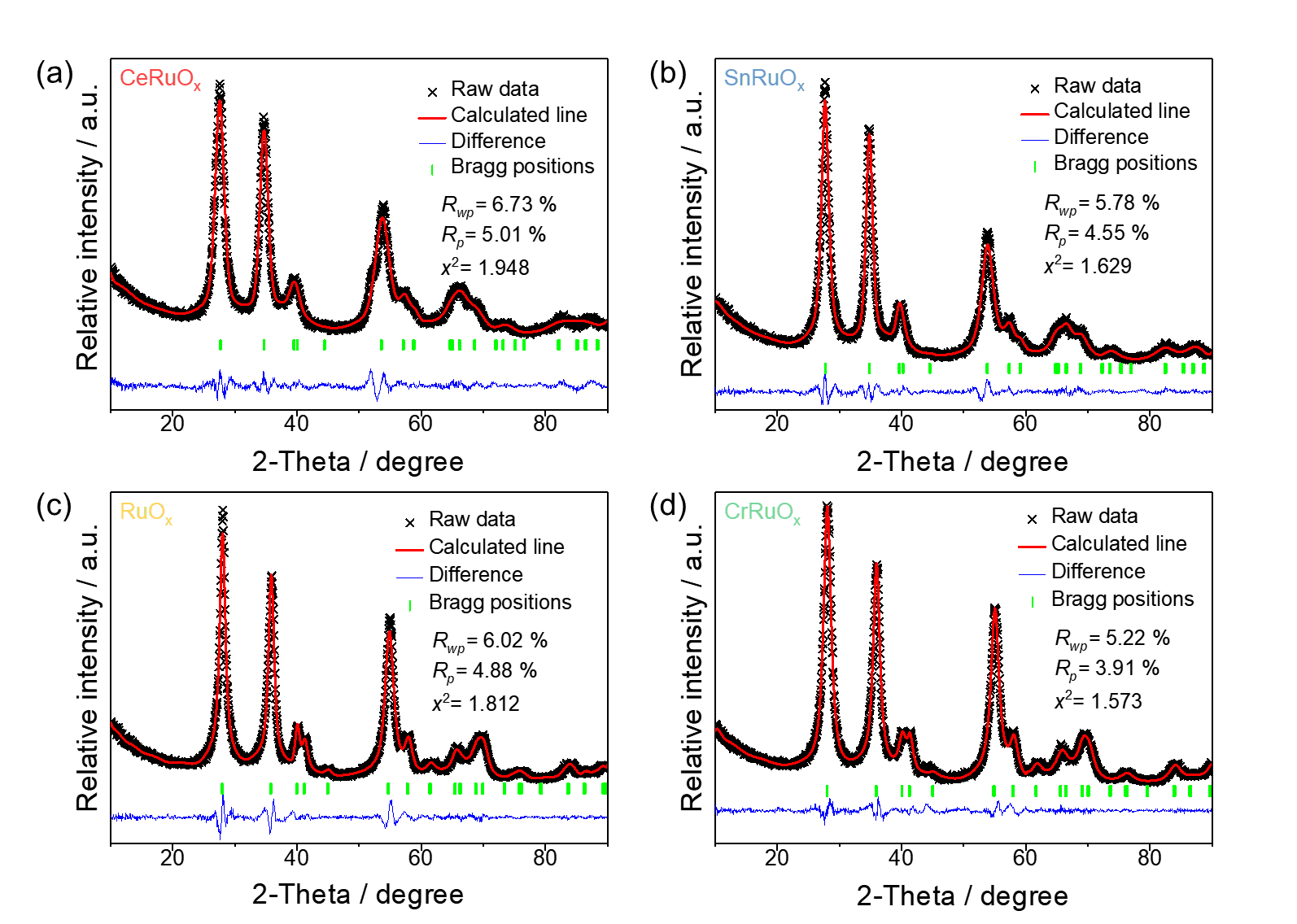


**Supplementary Fig. 51 | Rietveld refinement analysis of XRD patterns.** (a) CeRuO**_x_**, (b) SnRuO**_x_**, (c) RuO**_x_**, (d) CrRuO**_x_**. The refined structure parameters are presented in Supplementary Table 1.


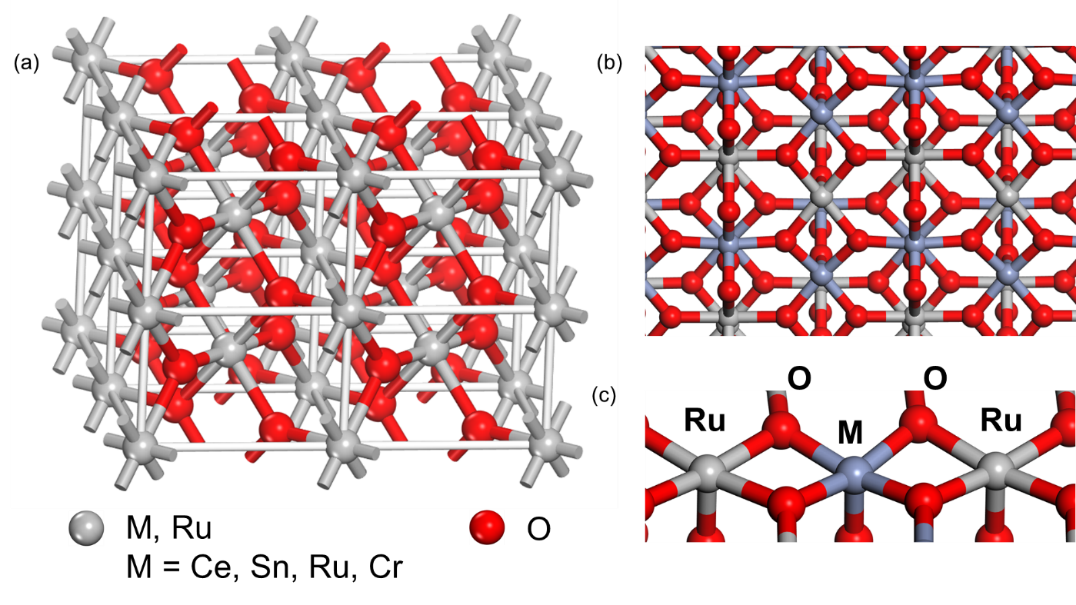


**Supplementary Fig. 52 | Structure model of MRuO_x_.** (a) Structure obtained from Rietveld refinement. The gray ball represent 1/2M and 1/2Ru as the M and Ru atoms are randomly distributed, showing the nature of solid solution. To vividly display the structure motives of MRuO**_x_**, half of the gray atoms were recolored with blue (b, c). The primary structure motif of Ru-O-M can be observed.


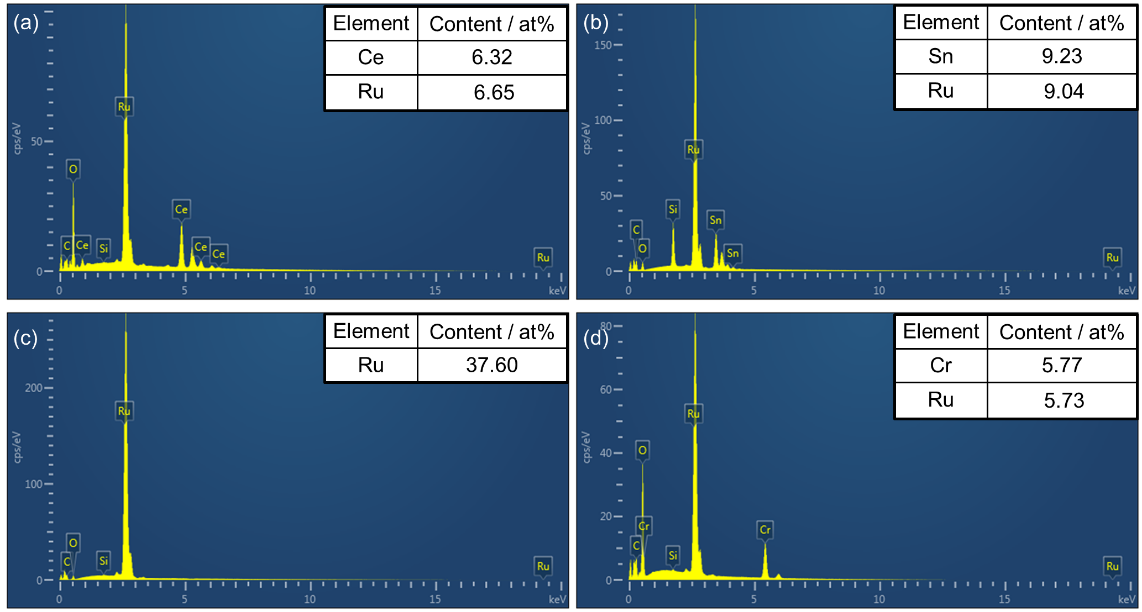


**Supplementary Fig. 53 | EDX spectra of MRuO_x_.** (a) CeRuO**_x_**, (b) SnRuO**_x_**, (c) RuO**_x_**, (d) CrRuO**_x_**.

**Supplementary Fig. 54 | O 1*s* XPS spectra of MRuO_x_.** The fitting results can be found in Supplementary Table 3.

**Supplementary Note 6 | Determine the LOM ratio during OER via in situ DEMS measurements.**

The DEMS measurements are conducted with MRu^16^O_x_ as the catalyst and ^18^O-isotope labelled 0.5 M H_2_SO_4_ or 0.1 M PBS (pH = 6) as electrolyte. We first carried out DEMS measurements on commercial IrO_2_ (recognized to follow AEM path) as a control to calculate the ratio of H_2_^16^O in H_2_^18^O. As shown in Supplementary Fig. 55a-c, the ratio of ^16^O/^18^O shows the minimal difference (ranging in 6.3-6.8 %) during 5 LSV runs, which is in line with the absence of the LOM path. To eliminate the artificial averaged signal caused by lattice oxygen exchange during LSV run, we extrapolated the ratio of ^16^O/^18^O to the onset potential (determined from the onset time of the signal of total oxygen according to the previous reports^4^) of first LSV run. As presented in Supplementary Fig. 55d, e, the ^16^O/^18^O product ratio is estimated to be 6.56 %, implying the ratio of H_2_^16^O in H_2_^18^O is ~6.16 %. With this baseline in mind, we move on to analyze the results of MRuO_x_, where the ^18^O-labeled 0.5 M H_2_SO_4_ (pH = 0) and 0.1 M PBS (pH = 6) aqueous are used as electrolyte (Supplementary Fig. 56-63). For RuO_x_ and CrRuO_x_, the ratio of ^16^O/^18^O keeps decreasing with increased LSV scans (Supplementary Fig. 58, 59, 62, 63), which is derived from the consumption of ^16^O-lattice oxygen during OER, suggesting the participation of LOM for these two samples in pH = 0 and 6. Based on the extrapolated ^16^O/^18^O ratio at the onset potential, the ratio of LOM in RuO_x_ and CrRuO_x_ is calculated to be 12.6 % and 36.7 % respectively (Supplementary Fig. 58-59). For SnRuO_x_, the ^16^O/^18^O product ratio keeps still at ~6.3 % for different LSV runs (Supplementary Fig. 57c, 61c), which is close to that obtained on commercial IrO_2_. After extrapolating to OER onset, the ^16^O/^18^O in the product is estimated to be 6.85 % and 7.06 % in 0.5 M H_2_SO_4_ and 0.1 M PBS (Supplementary Fig. 57e, 61e), in line with that of the commercial IrO_2_, further verified the absence of LOM. For CeRuO_x_, however, more complicated results are presented. For DEMS conducted with ^18^O-labeled 0.1 M PBS (pH = 6) aqueous, a consistent ^16^O/^18^O product ratio of ~6.4 % is observed for 5 consecutive LSV scans (Supplementary Fig. 60c), resembles that of SnRuO_x_ and commercial IrO_2_. Besides, the ^16^O/^18^O in the product is quantified to be 6.87 % at OER onset (Supplementary Fig. 60e), demonstrating the absence of LOM. For DEMS measured in ^18^O-labeled 0.5 M H_2_SO_4_, the ratio of ^16^O/^18^O shows a slight increase from ~6.5 % for the first 3 LSV scans to 7.0 % and 7.1 % for 4^th^ and 5^th^ LSV scan (Supplementary Fig. 56c). Besides, we find the ratio of ^32^O_2_ in the products gradually increase after the 3^rd^ LSV scan (Supplementary Fig. 56b). Combined with the DFT calculation on Ce vacancies, we think this variation may derive from the dissolution of Ce after continuously scanning to 1.5 V *vs.* RHE, which decreases the overpotential of LOM pathway and lead to a crossover between AEM and LOM. Thus, it is understandable that the extrapolated ^16^O/^18^O ratio at OER onset of the first LSV scan is calculated to be 6.88 %, resembles that obtained in ^18^O-labeled 0.1 M PBS, indicating the absence of LOM on CeRuO_x_. The LOM ratio determined in 0.5 M H_2_SO_4_ is then used to calculate the theoretical OER overpotential (with AEM and LOM included) of RuO_x_ and CrRuO_x_.


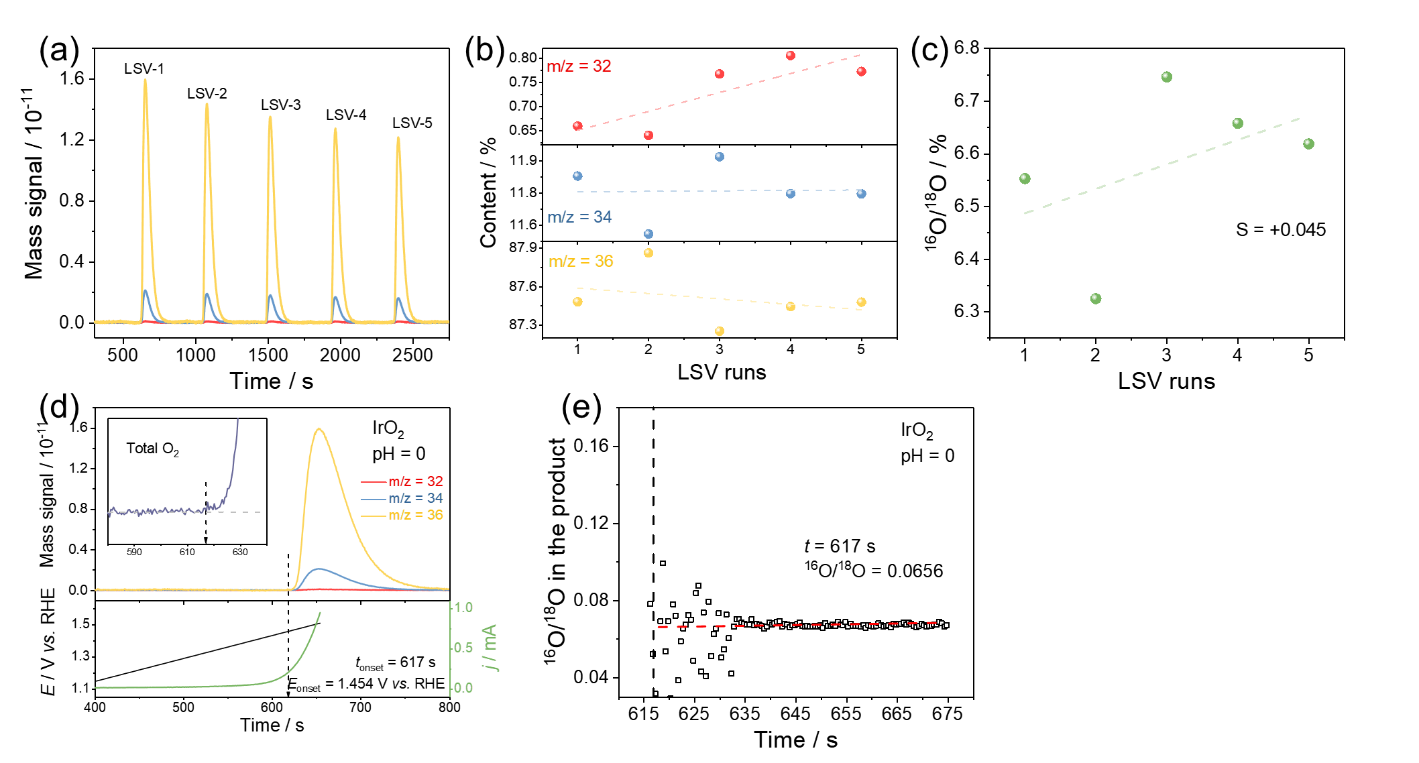


**Supplementary Fig. 55 |** ***In situ* DEMS results of IrO_2_.** (a) DEMS mass spectrum of IrO_2_ for 5 consecutive LSV run; (b) content of ^32^O_2_, ^34^O_2_, ^36^O_2_ in the products of 5 LSV run; (c) the ratio of ^16^O/^18^O in the products of 5 LSV run; (d) the mass signal of O_2_, current and potential as a function of time in the first LSV run, the insert show the total O_2_ evolved, which is used to determine the onset time, thus potential of OER; (e) extrapolation to calculate the proportion of LOM during OER.


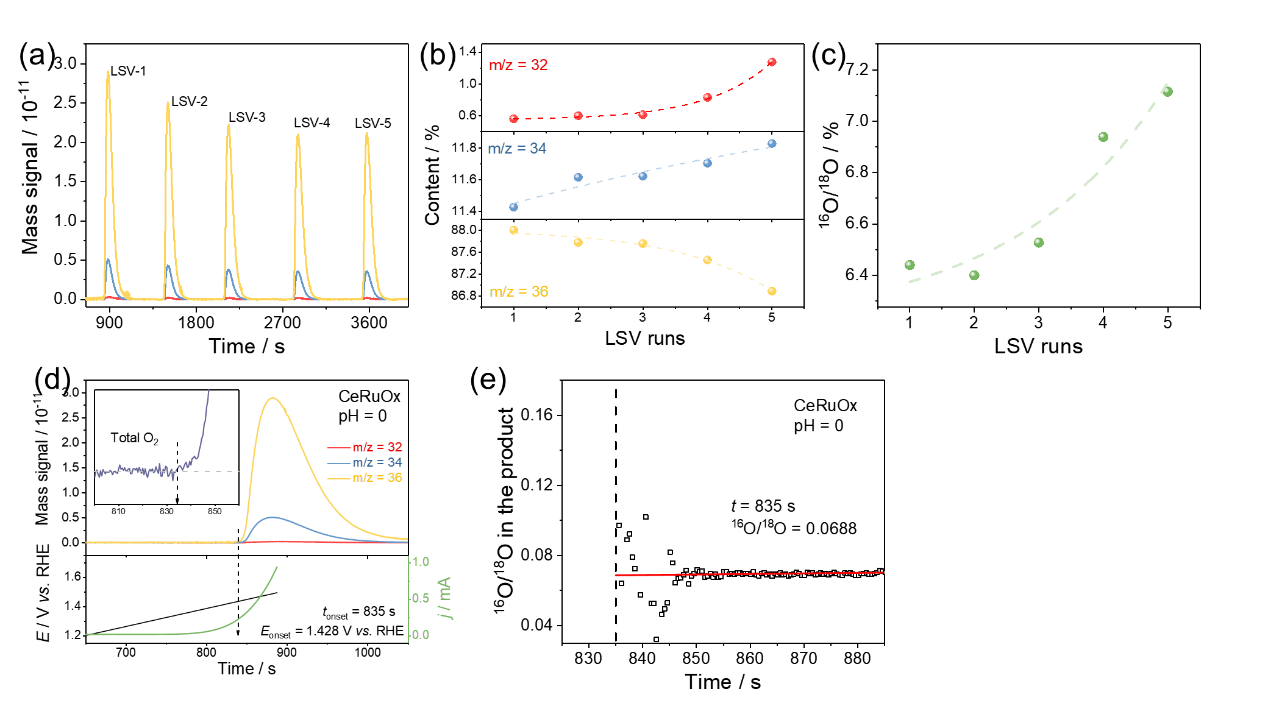


**Supplementary Fig. 56 |** ***In situ* DEMS results of CeRuO_x_** **in 0.5 M H_2_SO_4_.** (a) DEMS mass spectrum of CeRuO_x_ for 5 consecutive LSV run; (b) content of ^32^O_2_, ^34^O_2_, ^36^O_2_ in the products of 5 LSV run; (c) the ratio of ^16^O/^18^O in the products of 5 LSV run; (d) the mass signal of O_2_, current and potential as a function of time in the first LSV run, the insert show the total O_2_ evolved, which is used to determine the onset time, thus potential of OER; (e) extrapolation to calculate the proportion of LOM during OER. The test are performed in ^18^O-labelled 0.5 M H_2_SO_4_.


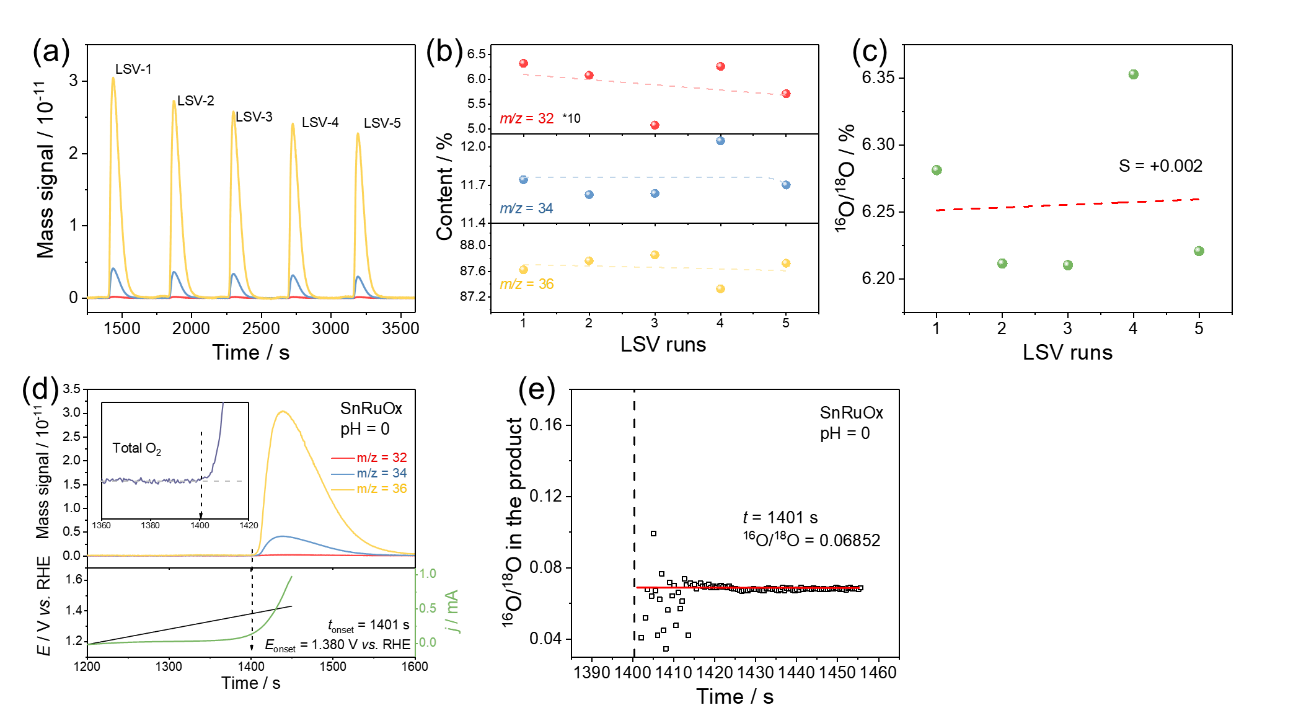


**Supplementary Fig. 57 |** ***In situ* DEMS results of SnRuO_x_** **in 0.5 M H_2_SO_4_.** (a) DEMS mass spectrum of SnRuO_x_ for 5 consecutive LSV run; (b) content of ^32^O_2_, ^34^O_2_, ^36^O_2_ in the products of 5 LSV run; (c) the ratio of ^16^O/^18^O in the products of 5 LSV run; (d) the mass signal of O_2_, current and potential as a function of time in the first LSV run, the insert show the total O_2_ evolved, which is used to determine the onset time, thus potential of OER; (e) extrapolation to calculate the proportion of LOM during OER. The test are performed in ^18^O-labelled 0.5 M H_2_SO_4_.


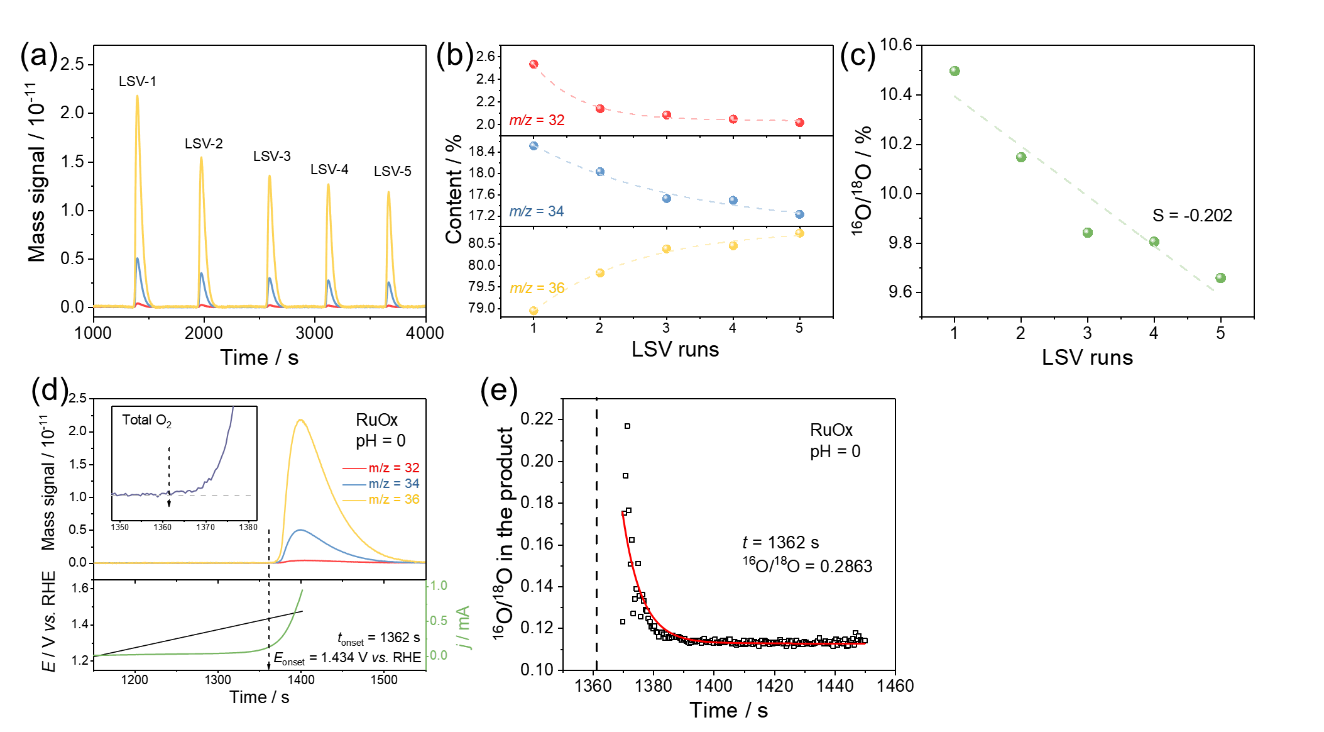


**Supplementary Fig. 58 |** ***In situ* DEMS results of RuO_x_** **in 0.5 M H_2_SO_4_.** (a) DEMS mass spectrum of RuO_x_ for 5 consecutive LSV run; (b) content of ^32^O_2_, ^34^O_2_, ^36^O_2_ in the products of 5 LSV run; (c) the ratio of ^16^O/^18^O in the products of 5 LSV run; (d) the mass signal of O_2_, current and potential as a function of time in the first LSV run, the insert show the total O_2_ evolved, which is used to determine the onset time, thus potential of OER; (e) extrapolation to calculate the proportion of LOM during OER. The test are performed in ^18^O-labelled 0.5 M H_2_SO_4_.


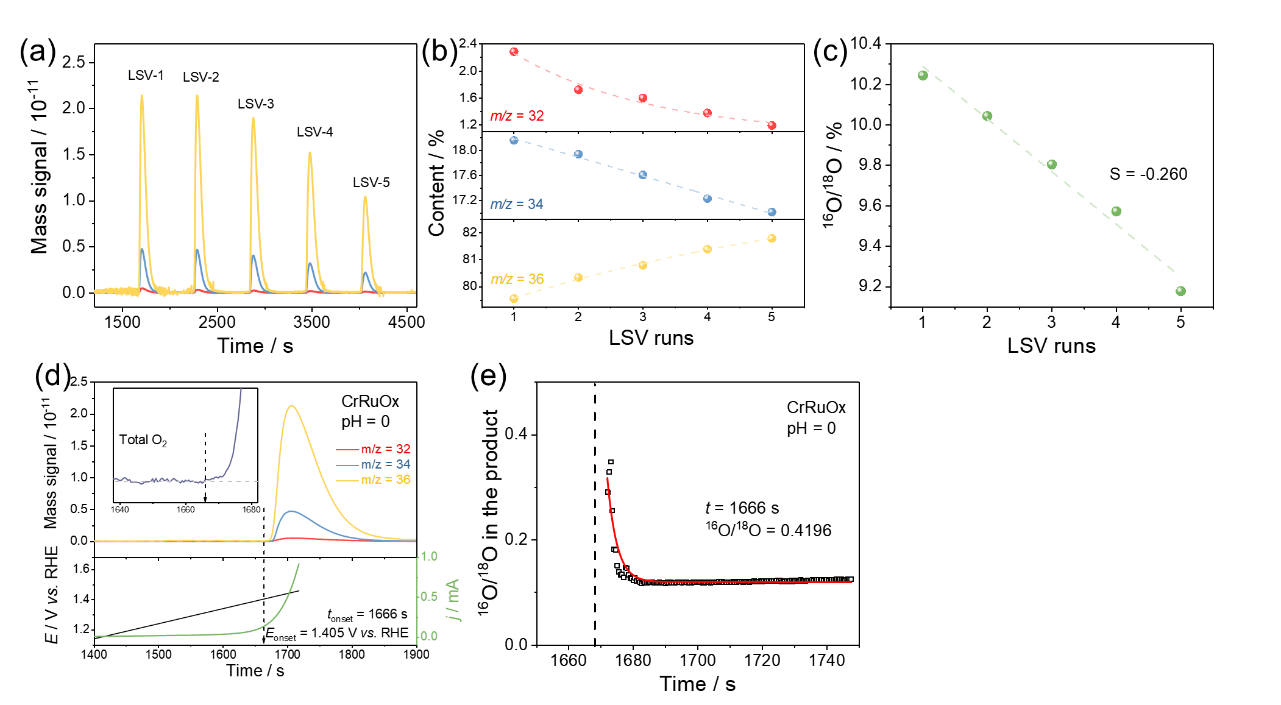


**Supplementary Fig. 59 |** ***In situ* DEMS results of CrRuO_x_** **in 0.5 M H_2_SO_4_.** (a) DEMS mass spectrum of CrRuO_x_ for 5 consecutive LSV run; (b) content of ^32^O_2_, ^34^O_2_, ^36^O_2_ in the products of 5 LSV run; (c) the ratio of ^16^O/^18^O in the products of 5 LSV run; (d) the mass signal of O_2_, current and potential as a function of time in the first LSV run, the insert show the total O_2_ evolved, which is used to determine the onset time, thus potential of OER; (e) extrapolation to calculate the proportion of LOM during OER. The test are performed in ^18^O-labelled 0.5 M H_2_SO_4_.


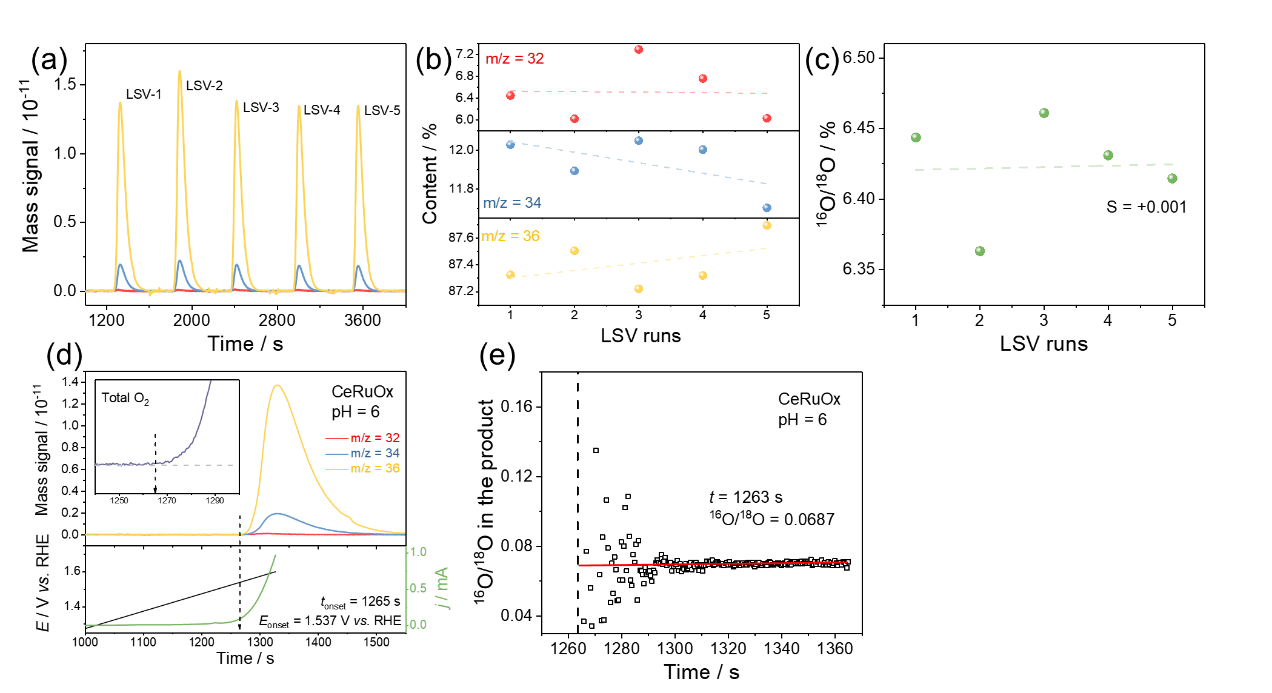


**Supplementary Fig. 60 |** ***In situ* DEMS results of CeRuO_x_** **in 0.1 M PBS.** (a) DEMS mass spectrum of CeRuO_x_ for 5 consecutive LSV run; (b) content of ^32^O_2_, ^34^O_2_, ^36^O_2_ in the products of 5 LSV run; (c) the ratio of ^16^O/^18^O in the products of 5 LSV run; (d) the mass signal of O_2_, current and potential as a function of time in the first LSV run, the insert show the total O_2_ evolved, which is used to determine the onset time, thus potential of OER; (e) extrapolation to calculate the proportion of LOM during OER. The test are performed in ^18^O-labelled 0.1 M PBS.


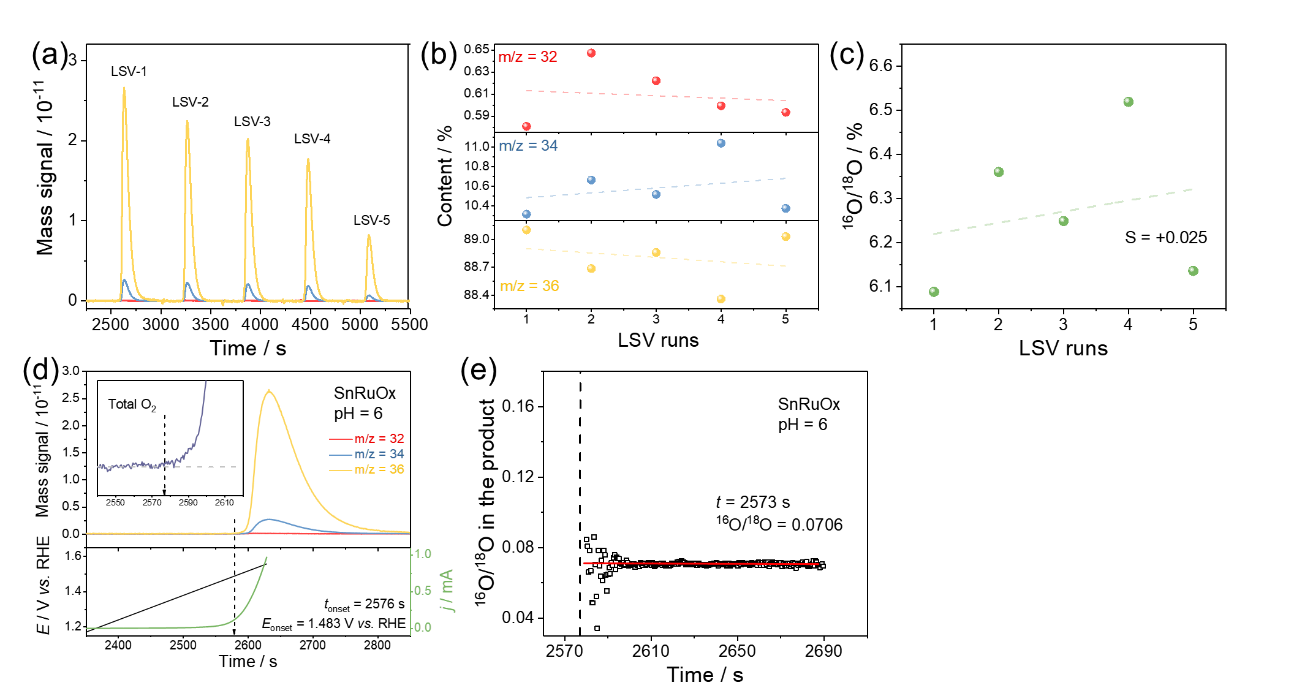


**Supplementary Fig. 61 |** ***In situ* DEMS results of SnRuO_x_** **in 0.1 M PBS.** (a) DEMS mass spectrum of SnRuO_x_ for 5 consecutive LSV run; (b) content of ^32^O_2_, ^34^O_2_, ^36^O_2_ in the products of 5 LSV run; (c) the ratio of ^16^O/^18^O in the products of 5 LSV run; (d) the mass signal of O_2_, current and potential as a function of time in the first LSV run, the insert show the total O_2_ evolved, which is used to determine the onset time, thus potential of OER; (e) extrapolation to calculate the proportion of LOM during OER. The test are performed in ^18^O-labelled 0.1 M PBS.


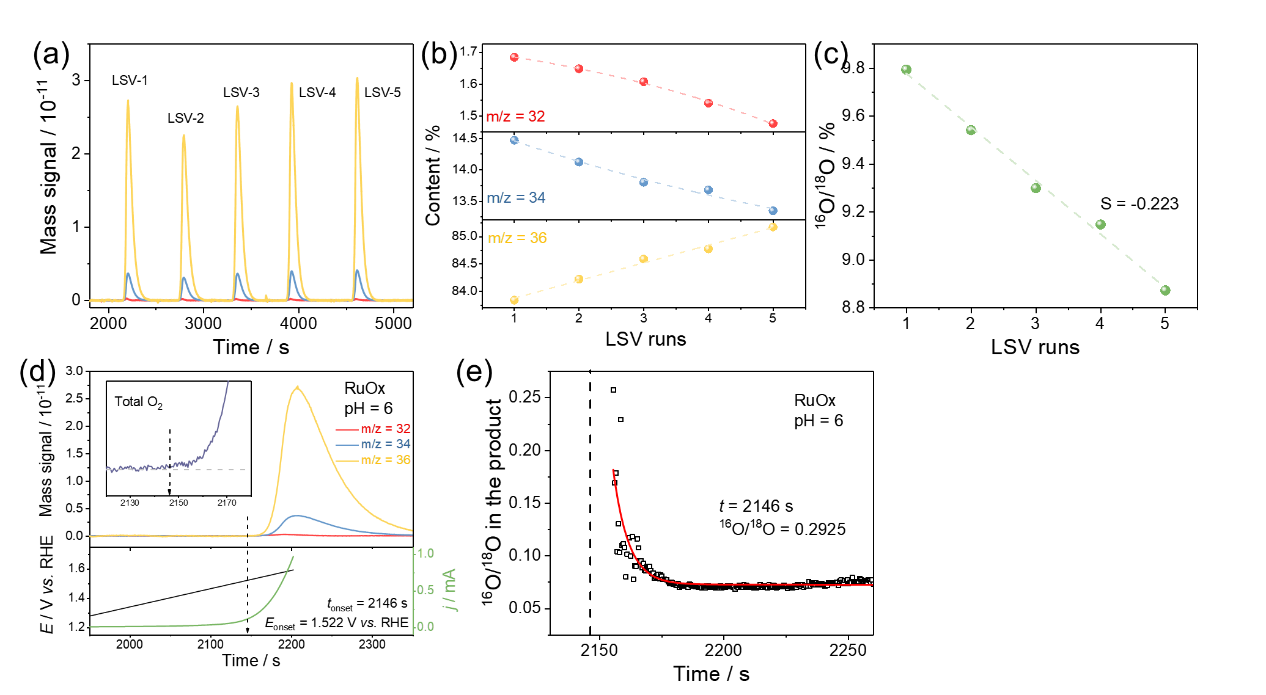


**Supplementary Fig. 62 |** ***In situ* DEMS results of RuO_x_** **in 0.1 M PBS.** (a) DEMS mass spectrum of RuO_x_ for 5 consecutive LSV run; (b) content of ^32^O_2_, ^34^O_2_, ^36^O_2_ in the products of 5 LSV run; (c) the ratio of ^16^O/^18^O in the products of 5 LSV run; (d) the mass signal of O_2_, current and potential as a function of time in the first LSV run, the insert show the total O_2_ evolved, which is used to determine the onset time, thus potential of OER; (e) extrapolation to calculate the proportion of LOM during OER. The test are performed in ^18^O-labelled 0.1 M PBS.


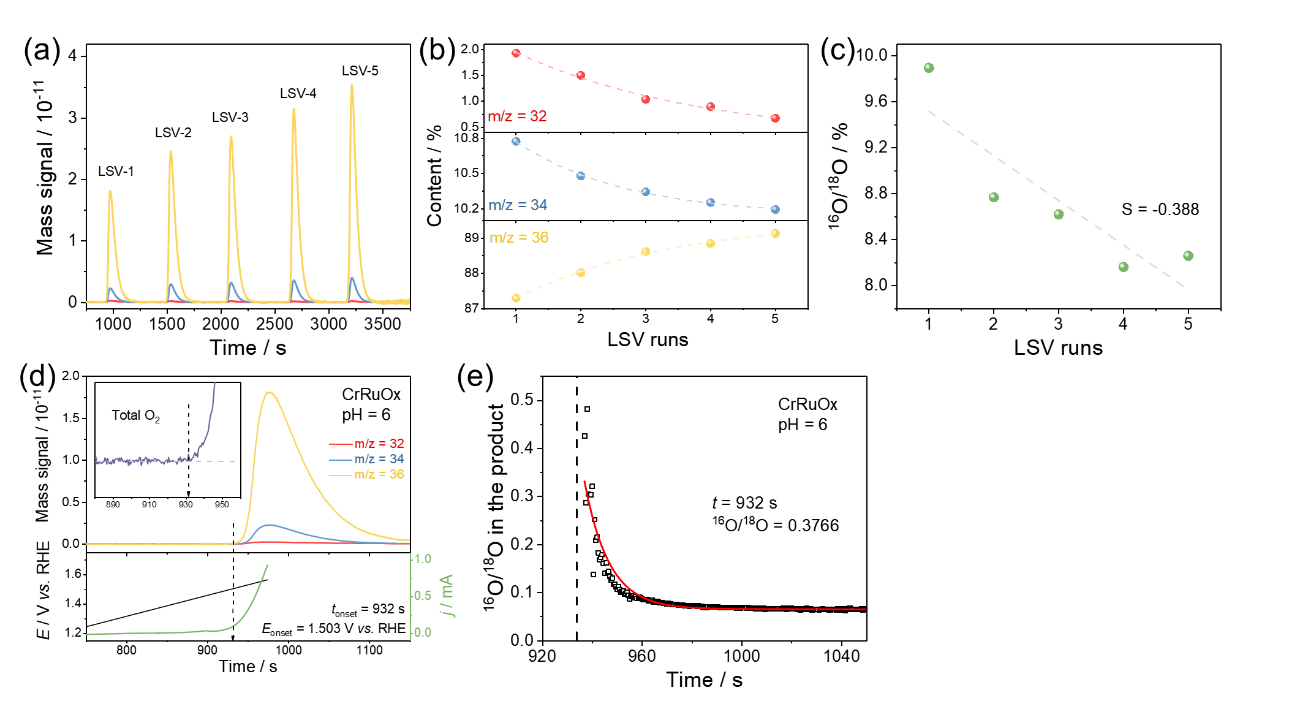


**Supplementary Fig. 63 |** ***In situ* DEMS results of CrRuO_x_** **in 0.1 M PBS.** (a) DEMS mass spectrum of CrRuO_x_ for 5 consecutive LSV run; (b) content of ^32^O_2_, ^34^O_2_, ^36^O_2_ in the products of 5 LSV run; (c) the ratio of ^16^O/^18^O in the products of 5 LSV run; (d) the mass signal of O_2_, current and potential as a function of time in the first LSV run, the insert show the total O_2_ evolved, which is used to determine the onset time, thus potential of OER; (e) extrapolation to calculate the proportion of LOM during OER. The test are performed in ^18^O-labelled 0.1 M PBS.

**Supplementary Note 7 | Comparison of the LOM ratio with the previous reports.**

Based on the DEMS measurements employed, the LOM content during OER is 12.6 % and 36.7 % for RuO_x_ and CrRuO_x_, respectively, which is different from the results in the previous work from Kwang S. Kim et al.^5^ and Chorkendorff et al.^6^. Specifically, only ~1% ^36^O_2_ is detected in the OER products catalyzed by Ni-Ru@Ru^16^O_x_ in 0.5 M H_2_SO_4_ made from ^18^O-water according to Kwang S. Kim et al., implying the low AEM content and high LOM ratio during OER. However, according to results from Chorkendorff et al., the amorphous RuO_x_ prepared show low content (~0.2%) of LOM during OER. Such difference may derive from the different property of the catalysts used. For instance, the recent work from Haotian Wang et al.^7^ revealed the absence of LOM during OER for Ni-RuO_2_. The LOM ratio is calculated to be ~14 % for RuO_2_ during OER according to the results from Sargent et al.^8^. Such difference has been discussed in the revised manuscript.

**Supplementary Note 8 | Determine the optimal loading of SnRuO_x_ on the electrode.**

As shown in Supplementary Fig. 64a, the geometrical area normalized current density is highly dependent on the catalyst loading on the working electrode. Higher catalyst loadings lead to lower overpotentials at the geometrical area normalized current density of 10 mA cm_geo_^-2^ (Supplementary Fig. 64b). To figure out whether the catalyst on the electrode fully expresses its activity, ECSAs under various catalyst loadings were collected and calculated (Supplementary Fig. 65). As summarized in Supplementary Fig. 64b, the ECSA normalized current density at 1.53 V *vs.* RHE keeps consistent with catalyst loading ranging between 3.54 and 41.65 μg_cat_ cm^-2^. With loading exceeding 41.65 μg_cat_ cm^-2^, *j*_ECSA_ decreased drastically. Thus, an optimized catalyst loading of 41.65 μg_cat_ cm^-2^ is selected for performance investigation.


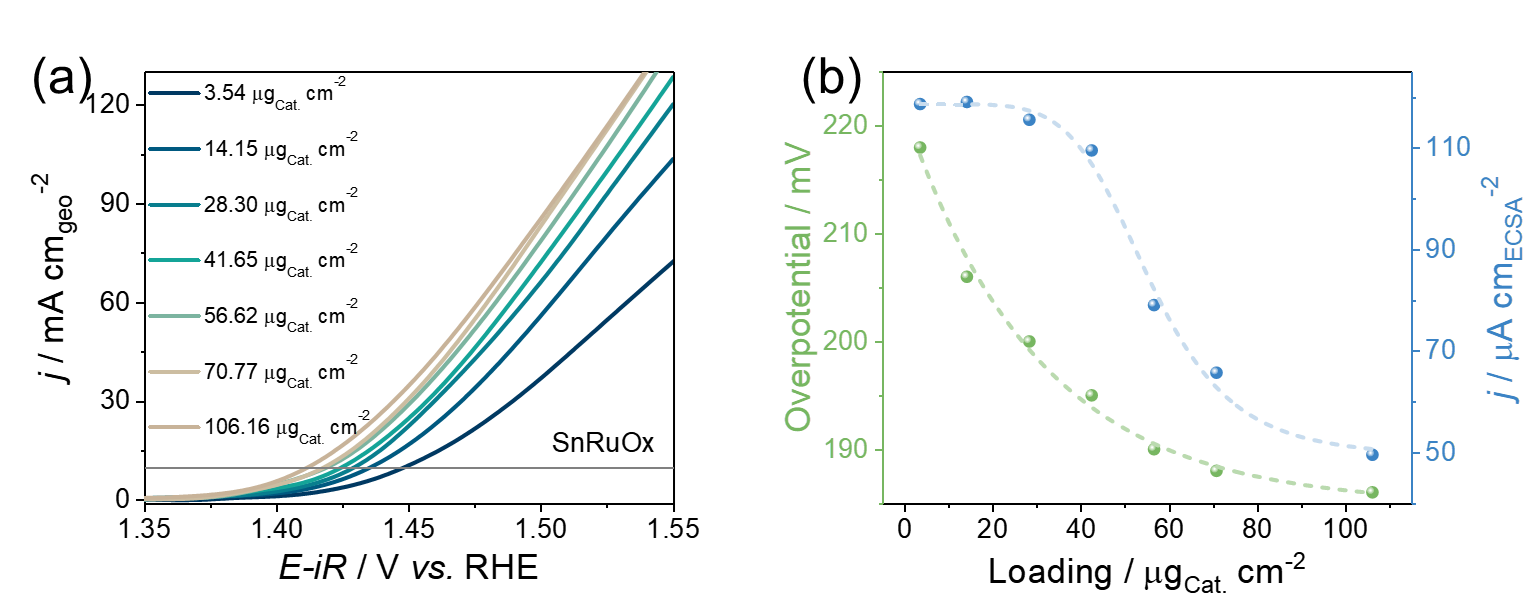


**Supplementary Fig. 64 |** **Catalytic activity with different catalyst loading.** (a) LSV curves of SnRuO_x_ with different catalyst loading; (b) the relation between the overpotential@10 mA cm^-2^, *j*_ECSA_@1.48 V *vs.* RHE and the catalyst loading.


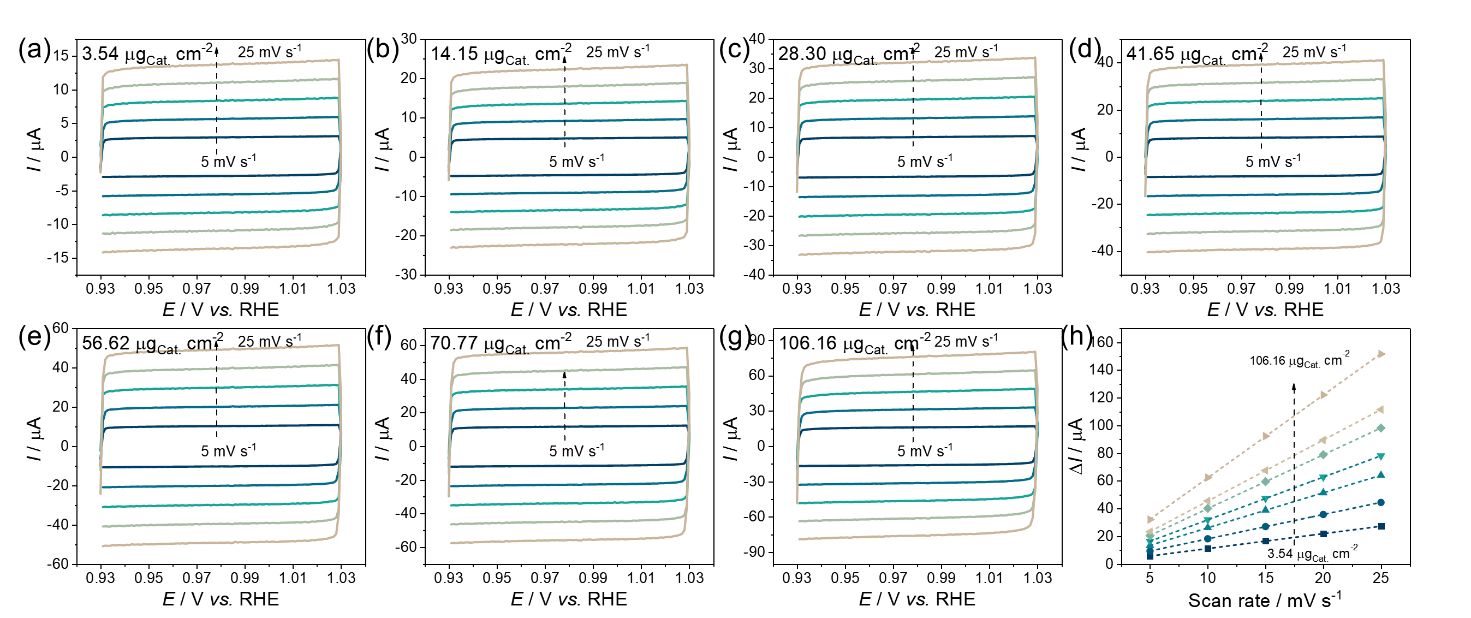


**Supplementary Fig. 65 | ECSA with various catalyst loading on the electrode.** (a-g) CV profiles of SnRuO_x_ with different catalyst loading in the non-Faradaic region of 0.93 – 1.03 V *vs.* RHE with the scan rate of 5, 10, 15, 20, 25 mV s^-1^, (h) C_dl_ plots obtained from the CV curves.


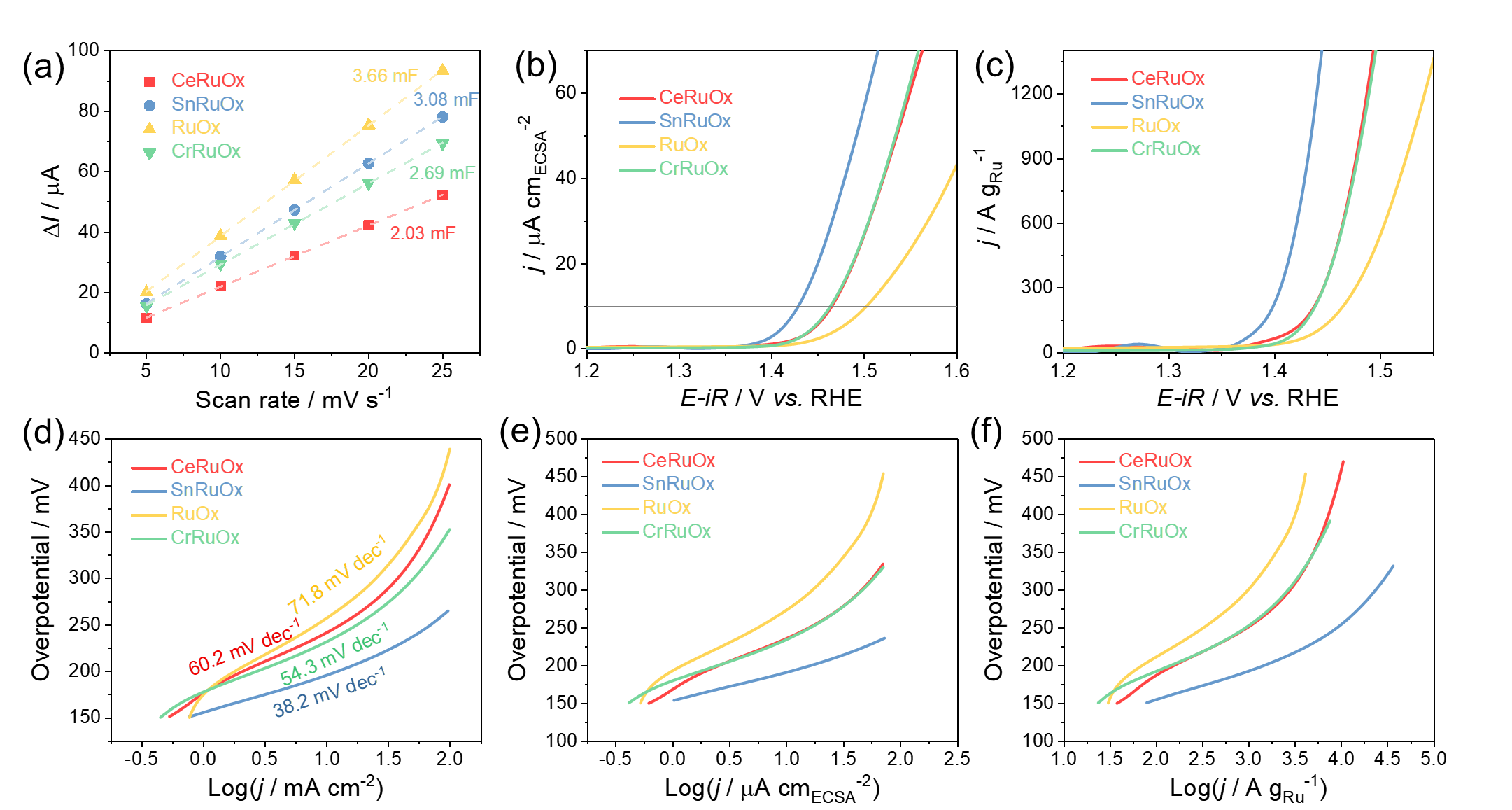


**Supplementary Fig. 66 | OER performance of MRuO_x_.** (a) C_dl_ plots for ECSA determination; (b) Normalized LSV curves to the ECSA; (c) LSV curves of MRuO**_x_** normalized by the Ru loading on the electrode; (d) Tafel plots derived from the LSV curves normalized by the geometry area of electrode, (e) Tafel plots derived from the LSV curves normalized by the Ru loading on the electrode, (f) Tafel plots derived from the LSV curves normalized by the ECSA.

**Supplementary Note 9 | Relation between the Tafel slope and OER rate determine step under AEM**

According to the previous reports, the Tafel slope can be used to evaluate the rate determine step during OER on AEM path. Their theoretical correspondence is^9-13^:

Step 1 (CUS + H_2_O → CUS-OH + H^+^ + e^-^) Tafel slope = 120 mV dec^-1^

Step 2 (CUS-OH → CUS-O + H^+^ + e^-^) Tafel slope = 40 mV dec^-1^

Step 3 (CUS-O + H_2_O → CUS-OOH + H^+^ + e^-^) Tafel slope = 24 mV dec^-1^

Step 4 (CUS-OOH → CUS + H^+^ + O_2_ + e^-^) Tafel slope = 17 mV dec^-1^

However, these kinetic analyses are based on the highest intermediates coverage on the active sites. More accurate microkinetic analyses indicate that moderate coverage will lead to a mixture in the value of Tafel slope. For instance, if we assume step 2 as the rate determine step, a Tafel slope of 40 mV dec^-1^ can be obtained if the coverage of OH (*θ*_OH_) is 1. With the decrease in the *θ*_OH_, an increase in the Tafel slope can be observed. When the *θ*_OH_ equals 0, then a Tafel slope of 120 mV dec^-1^ can be calculated.

Practically, the coverage of one certain intermediate on the active sites will not be too high or too low. Thus, the Tafel slopes experimentally obtained are usually within a range instead of exactly equal to 120 mV dec^-1^, 40 mV dec^-1^, 24 mV dec^-1^ and 17 mV dec^-1^. For instance, the recently reported RuO_2_ NS and Sr_1.3_Ru_5_Ir_1_O_13.7_ exhibited Tafel slope of 38.2 mV dec^-1^ and 39 mV dec^-1^, respectively^8, 14^.

Here, the Tafel slope of CeRuO**_x_** and SnRuO**_x_** was calculated to be 60.2 mV dec^-1^ and 38.2 mV dec^-1^, implying that the rate determine step of OER is step 2 and step 3 on CeRuO**_x_** and SnRuO**_x_** respectively, which is in line with the DFT calculation.


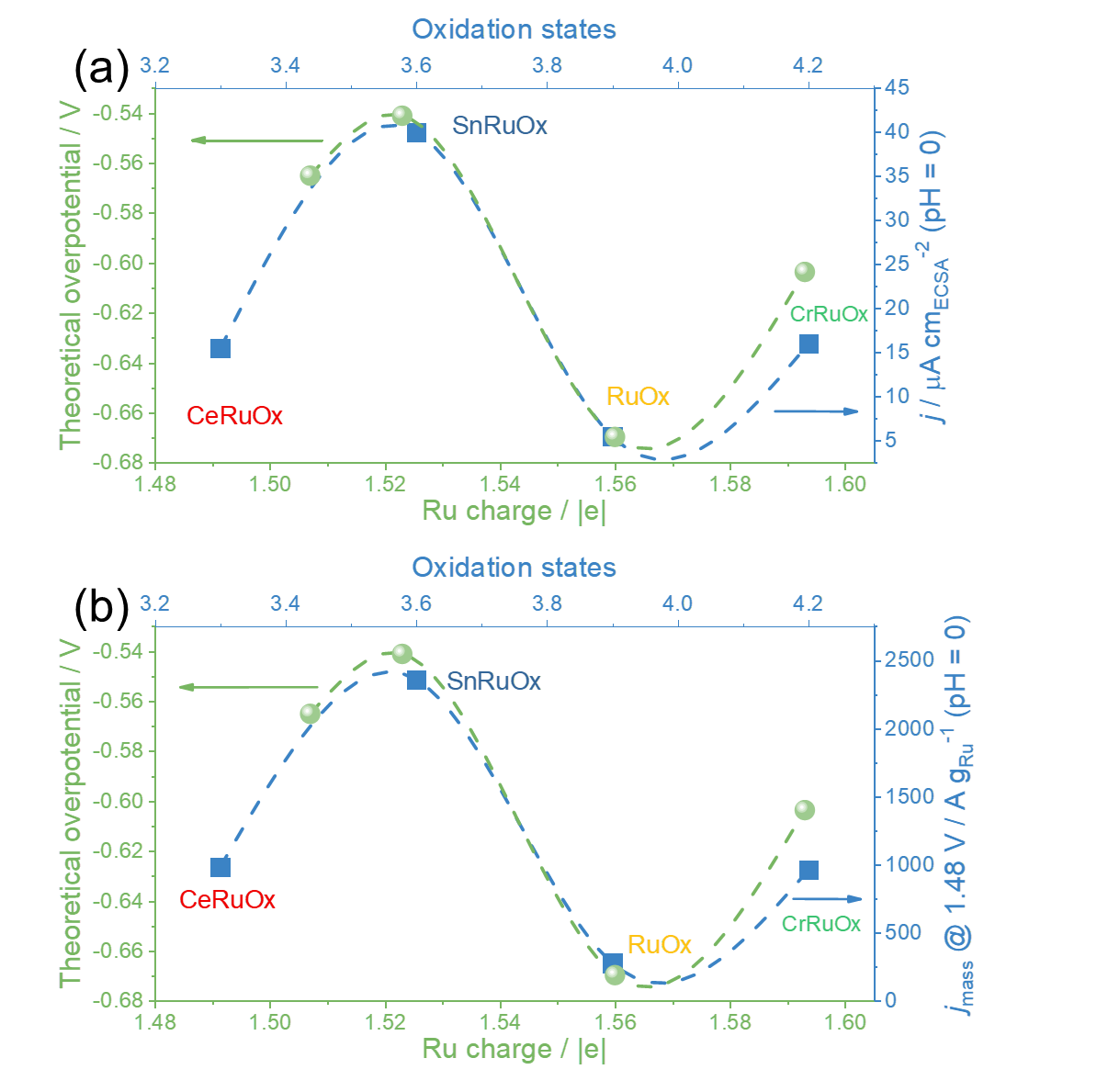


**Supplementary Fig. 67 | Relation between the Ru oxidation states and OER activity.** The OER catalytic activity of MRuO**_x_** is compared on the scale of (a) specific activity normalized by ECSA at 1.48 V *vs.* RHE and (b) mass activity at 1.48 V *vs.* RHE.


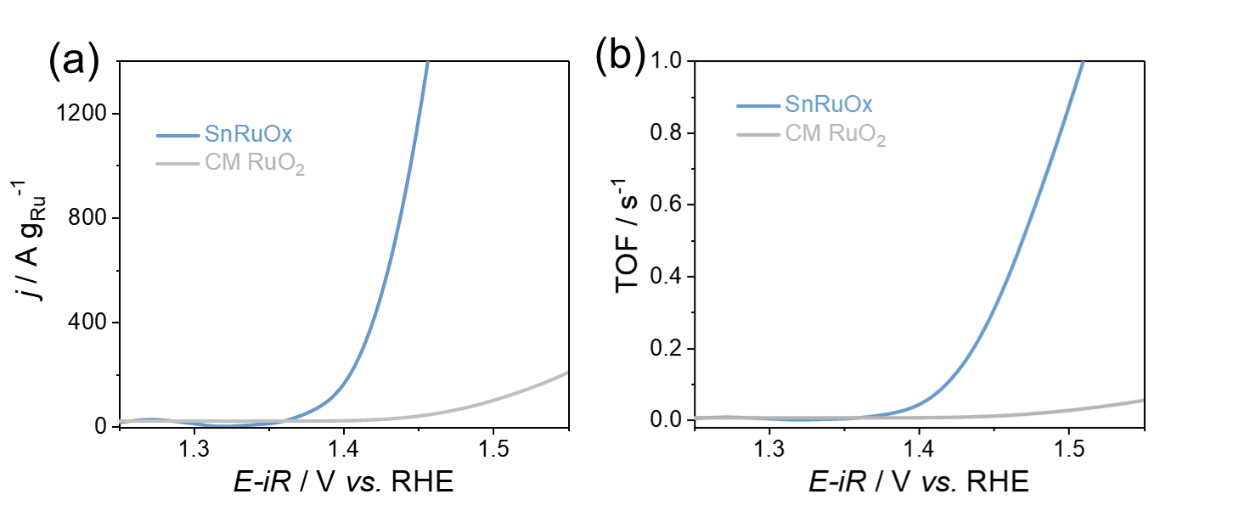


**Supplementary Fig. 68 | OER performance compared with commercial RuO_2_.** The mass activity (a) and TOF (b) of SnRuO**_x_** and CM RuO_2_.


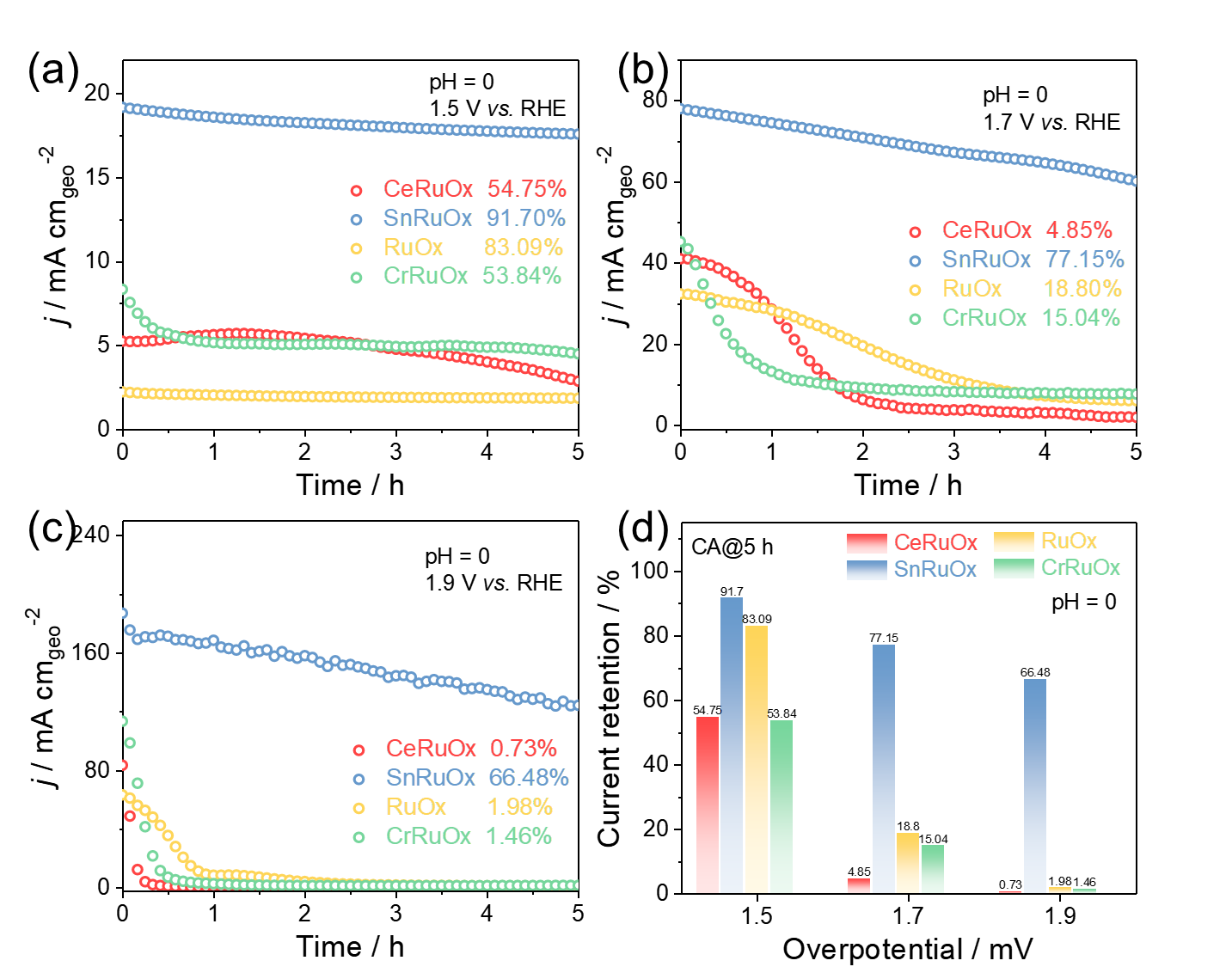


**Supplementary Fig. 69 | OER stability.** Chronoamperometry curves of MRuO**_x_** operated at (a) 1.5 V *vs.* RHE, (b) 1.7 V *vs.* RHE and (c) 1.9 V *vs.* RHE; (d) Current retention of MRuO_x_ after chronoamperometry test at various potential (1.5 V, 1.7 V and 1.9 V) for 5 h.

**Supplementary Fig. 70 | The relation between the S-number, Δ*G*_VRu_ and the Ru oxidation states in 0.5 M H_2_SO_4_.**


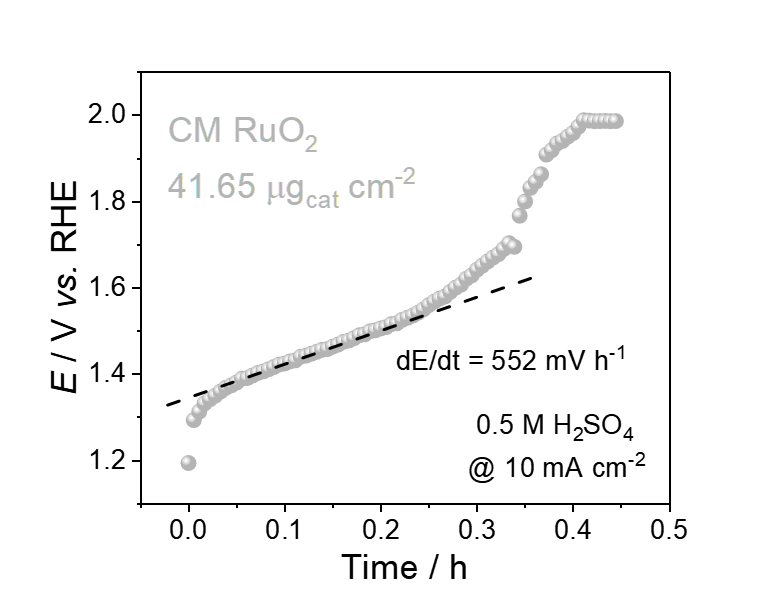


**Supplementary Fig. 71 | OER stability of commercial RuO_2_.** Chronopotentiometry curves of CM RuO_2_ operated at 10 mA cm^-2^. The degradation rate is estimated to be 552 mV h^-1^.

**Supplementary Note 10 | Clarify the contribution of Ru dissolution and M dissolution on the catalytic stability of MRuO_x_.**

To eliminate the dissolution of M on determine the stability of MRuO_x_, stability tests are also carried out in 0.1 M PBS (pH = 6) (Supplementary Fig. 72). Though the S-number based on M on the scale of Ru oxidation states takes almost identical trend to the variation of current retention for CeRuO_x_, SnRuO_x_, RuO_x_ and CrRuO_x_ (Supplementary Fig. 73a), which may lead to the conclusion that it is the stability of M that determine the stability of MRuO_x_. However, we think it is arbitrary to make such a conclusion, as the S-number based on Ru shares the same variation trend (Supplementary Fig. 73b). Thus, it cannot be confirmed whether it is the leaching of Ru or M that leads to the performance. To clarify the contribution of Ru and M leaching on performance degradation, the dissolved amount of Ru and M (compared to the total amount in catalyst) were analyzed (Supplementary Fig. 73c, d). Clearly, the variation in the dissolved Ru content is consistent with the performance degradation at pH = 6 (Supplementary Fig. 73c). While the M dissolution amount shows no dependence on the variation in activity decay (Supplementary Fig. 73d). Thus, we claim that it is the leaching of Ru rather than M during OER that is responsible for the performance degradation of MRuO_x_.


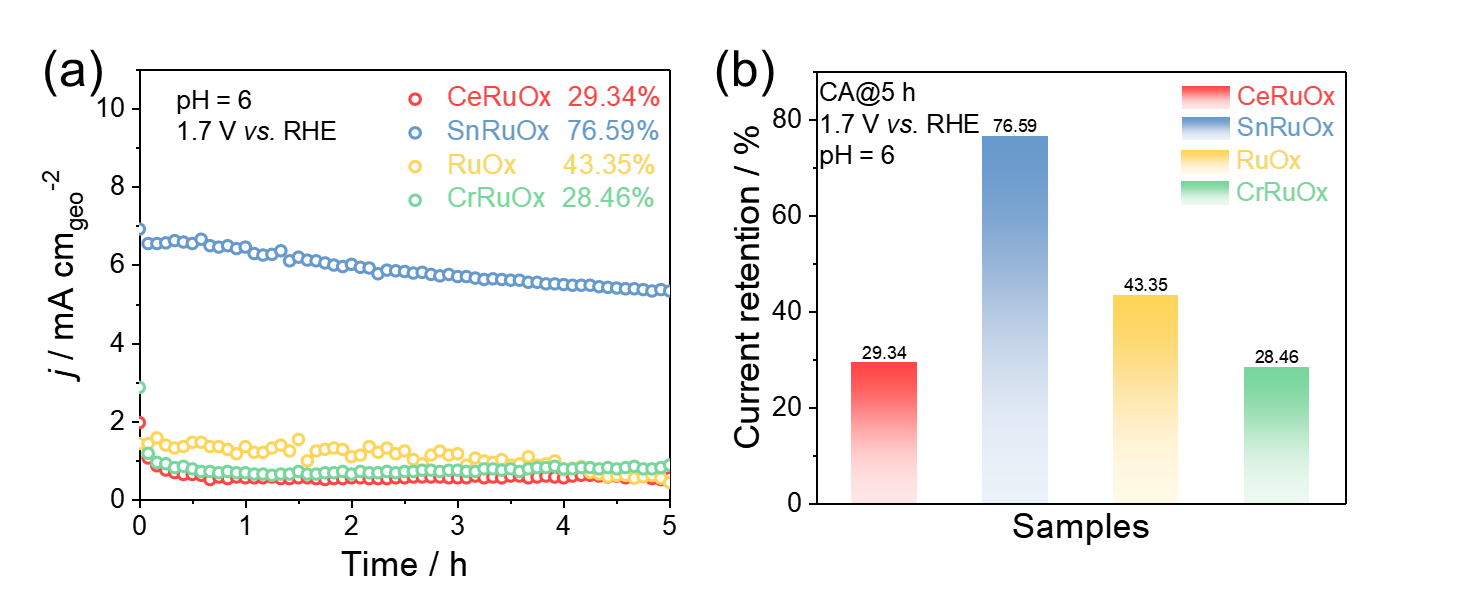


**Supplementary Fig. 72 | OER stability in 0.1 M PBS (pH = 6).** (a) Chronoamperometry curves of MRuO**_x_** operated at 1.7 V *vs.* RHE; (b) Current retention of MRuO_x_ after chronoamperometry test at 1.5 V vs. RHE for 5 h.


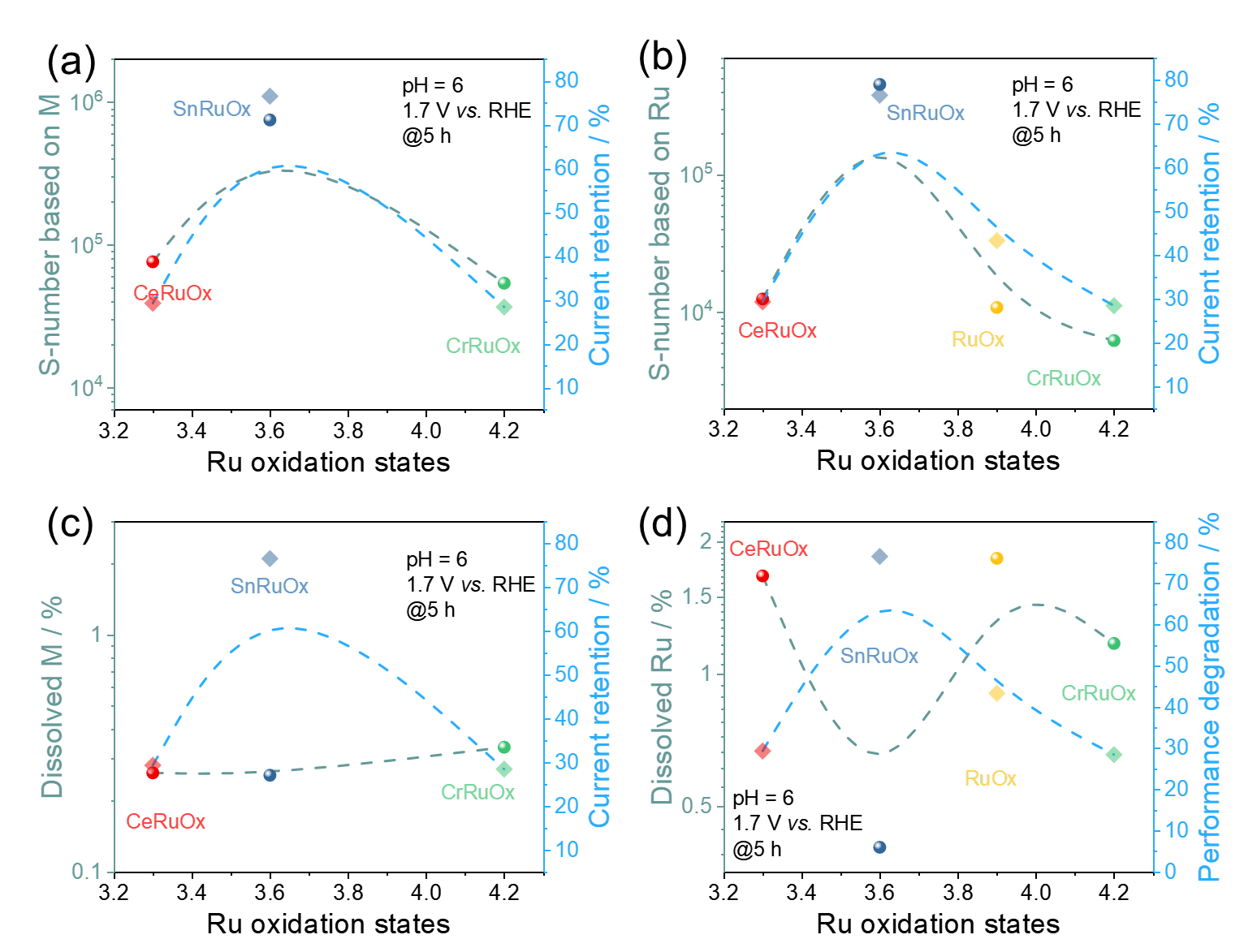


**Supplementary Fig. 73 | The relation between the S-number, Δ*G*_VRu_ and the Ru oxidation states in 0.1 M PBS.** (a, b) The relation between stability (represented by the current retention) and S-number based on (a) M and (b) Ru; (c, d) The relation between stability (represented by the current retention) and dissolution amount of (c) M and (d) Ru


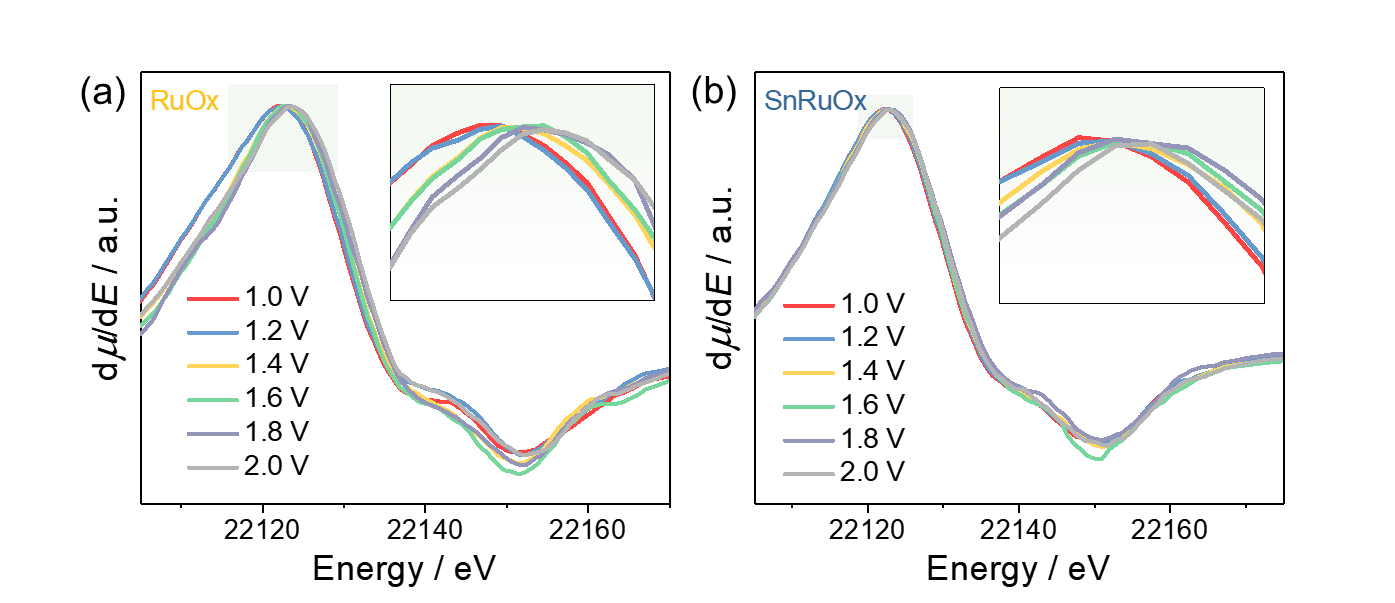


**Supplementary Fig. 74 | Valence variation during OER.** The first derivative of *in situ* XANES spectra obtain under various applied potential on (a) RuO**_x_** and (b) SnRuO**_x_**.


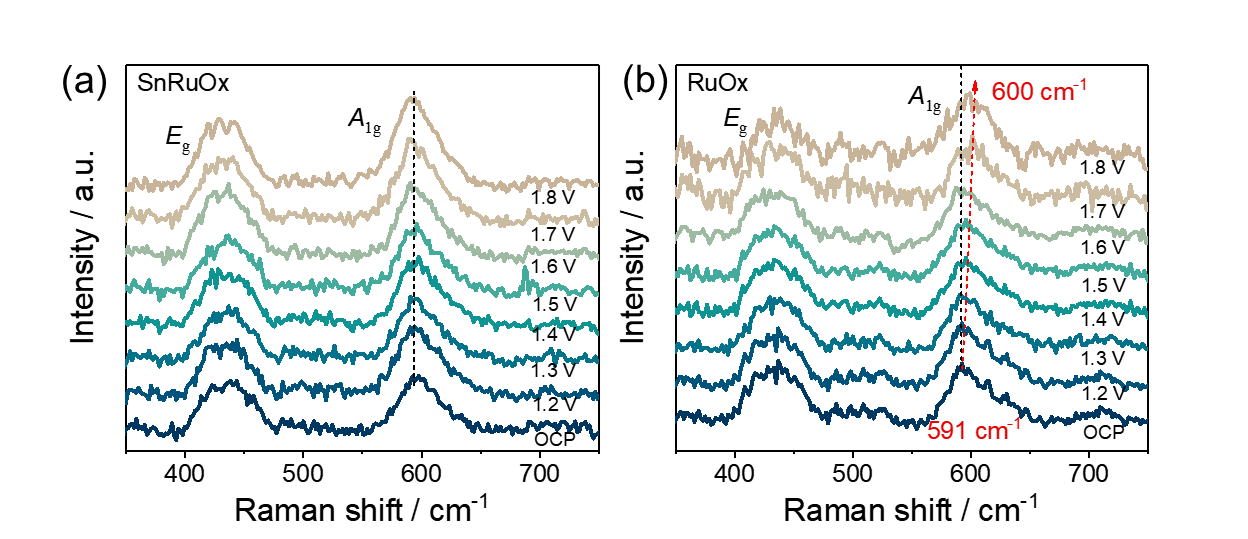


**Supplementary Fig. 75 | Structure variation during OER.** *In situ* Raman spectra obtained under various applied potential on (a) SnRuO**_x_** and (b) RuO**_x_**. The Raman peak assign to *E*_g_ and *A*_1g_ are similar to those previously reported on homemade RuO**_x_**^15^.


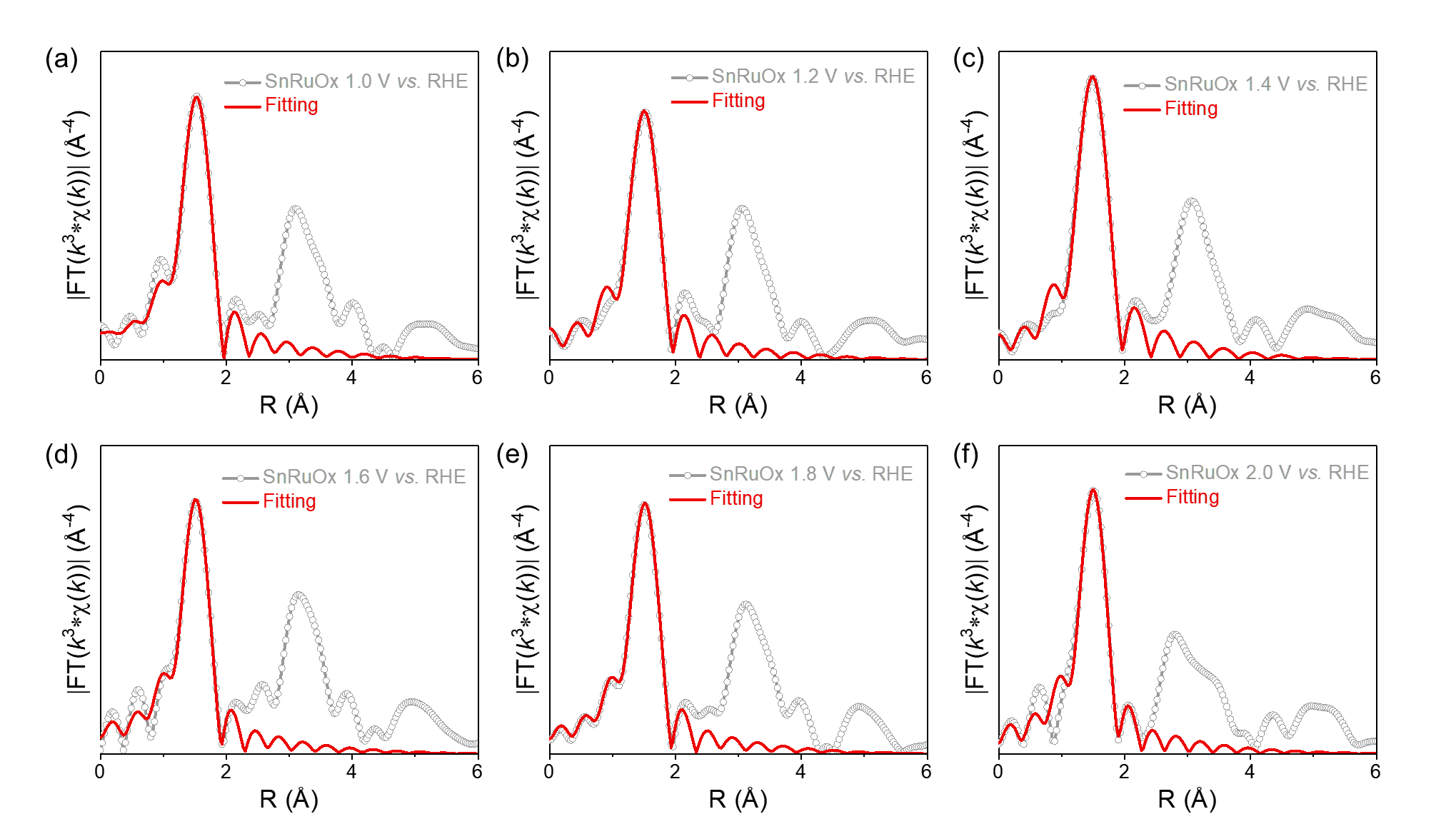


**Supplementary Fig. 76 | Fine structure analysis.** Fourier transforms of the *in situ* EXAFS spectra at Ru *K*-edge of SnRuO**_x_** and the corresponding fitting curve, without phase correction. (a) 1.0 V *vs.* RHE, (b) 1.2 V *vs.* RHE, (c) 1.4 V *vs.* RHE, (d) 1.6 V *vs.* RHE, (e) 1.8 V *vs.* RHE, (f) 2.0 V *vs.* RHE. The fitting results are presented in Supplementary Table 7


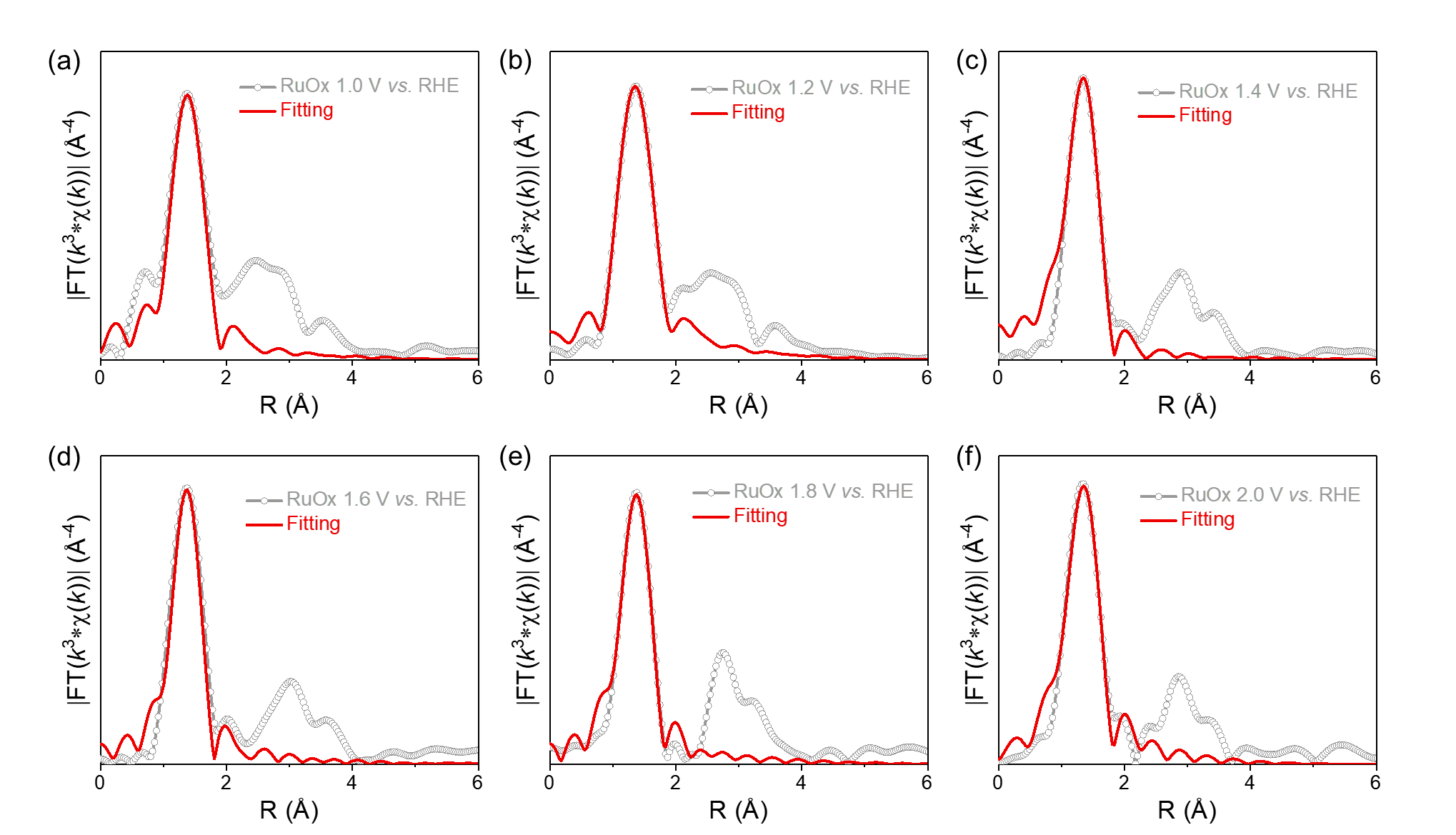


**Supplementary Fig. 77 | Fine structure analysis.** Fourier transforms of the *in situ* EXAFS spectra at Ru *K*-edge of RuO**_x_** and the corresponding fitting curve, without phase correction. (a) 1.0 V *vs.* RHE, (b) 1.2 V *vs.* RHE, (c) 1.4 V *vs.* RHE, (d) 1.6 V *vs.* RHE, (e) 1.8 V *vs.* RHE, (f) 2.0 V *vs.* RHE. The fitting results are presented in Supplementary Table 7


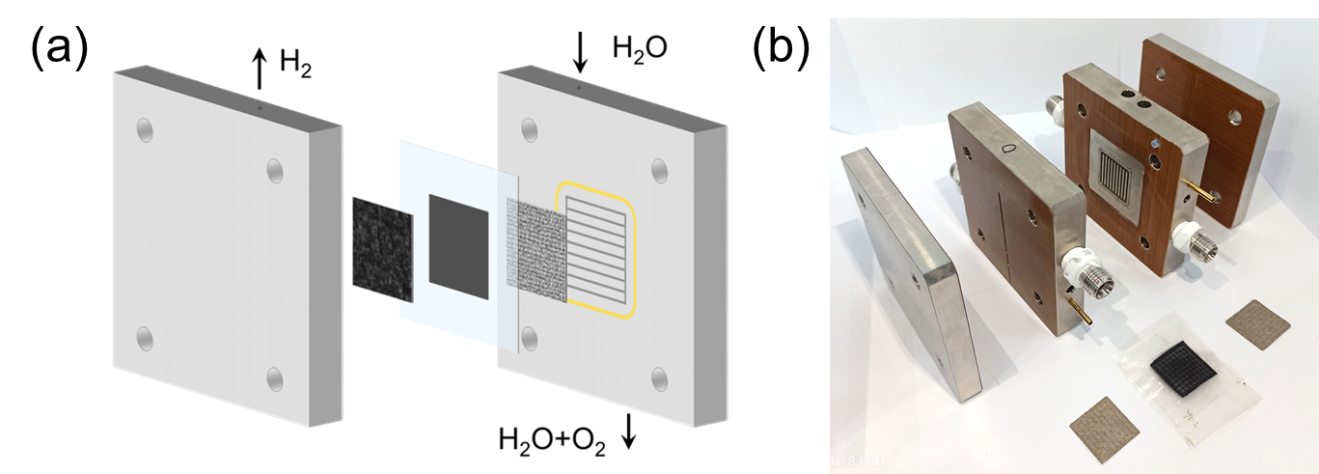


**Supplementary Fig. 78 | PEM electrolyzer demonstration.** (a) Schematic diagram of the PEM electrolyzer, (b) optical photo of the component of a PEM electrolyzer

**Supplementary Fig. 79 | PEM electrolyzer performance.** Polarization curve of the PEM electrolyzer using commercial RuO_2_ as anode catalyst.

**Supplementary Note 12 | To accurately determine the S-number of SnRuOx in PEM electrolyzer.**

During OER, the redeposition on metal parts, deposition in the membrane and escaping to the cathode side along with the water (determined by the electroosmotic drag) should be considered when monitoring the dissolved Ru in PEMWE. We thus performed modified ICP-PEMWE measurements according to the procedure and set-up proposed by Serhiy Cherevko et al.^16^ As presented in Supplementary Fig. 80a and b, the modified MEA setup without metallic parts in the anode and cathode water cycle is developed. Cell components involved in electronic conduction, namely Ti flowfields and current collectors are coated with Au to prevent the galvanic replacement of dissolved Ru. To suppress the redeposition of Ru and accurately determine the Ru in the cathode, MilliQ water is supplied with a flow rate of 25 mL min^-1^ for both the anode and cathode, which was not cycled during operation (Supplementary Fig. 80c-e). Considering the long-term operation will lead to the corrosion damage of Au-coated flow fields^16^, the measurement was limited to 10 h. The modified MEA setup is used to assemble a PEMWE single cell with the same catalyst loading as that presented in the manuscript. During a 10 hour operation at 1 A cm^-2^, the dissolved Ru in both the anode and cathode side of PEMWE are monitored by ICP-OES each hour. The Ru deposited in the membrane is estimated from the EDS spectrum of the ultrathin section of membrane after CP test (nominal thickness of 100 nm). The results are as follows.

As shown in Supplementary Fig. 81a, the cell voltage shows no sign of increase in the 10 h testing, in line with the robustness of SnRuO_x_. The Ru dissolution amount in the anode side gradually decreases with elongated running time (Supplementary Fig. 81b), i.e. from 0.144 % for 1^st^ hour to 0.075 % for 10^th^ hour, which is consistent with the exponentially decreased dissolution rate of Ru with time during a 1300 h testing (Supplementary Fig. 82). In 10 h operating, the dissolved amount of Ru in the cathode side is very low and show no variation trend, giving an average Ru dissolution amount of 0.01 %. We then move on to access the amount of Ru in the membrane with SEM and TEM. From the cross-section of MEA after stability test (Supplementary Fig. 83a), it is clear that the cathode catalyst layer, membrane and anode catalyst layer are well-maintained. EDS spectrum of membrane is collected from the cathode to anode catalyst layer (Supplementary Fig. 83b), with Ru and Sn content presented in Figure Supplementary Fig. 83c. Clearly, closer to the catalyst layer, the deposited metal content in the membrane is higher. In the TEM images of the ultrathin section of membrane, the catalyst layer and membrane are also observable (Supplementary Fig. 84a-c). The content of Ru and Sn was analyzed based on the EDS spectrum, with results presented in Supplementary Fig. 84d, e and Supplementary Table 10.


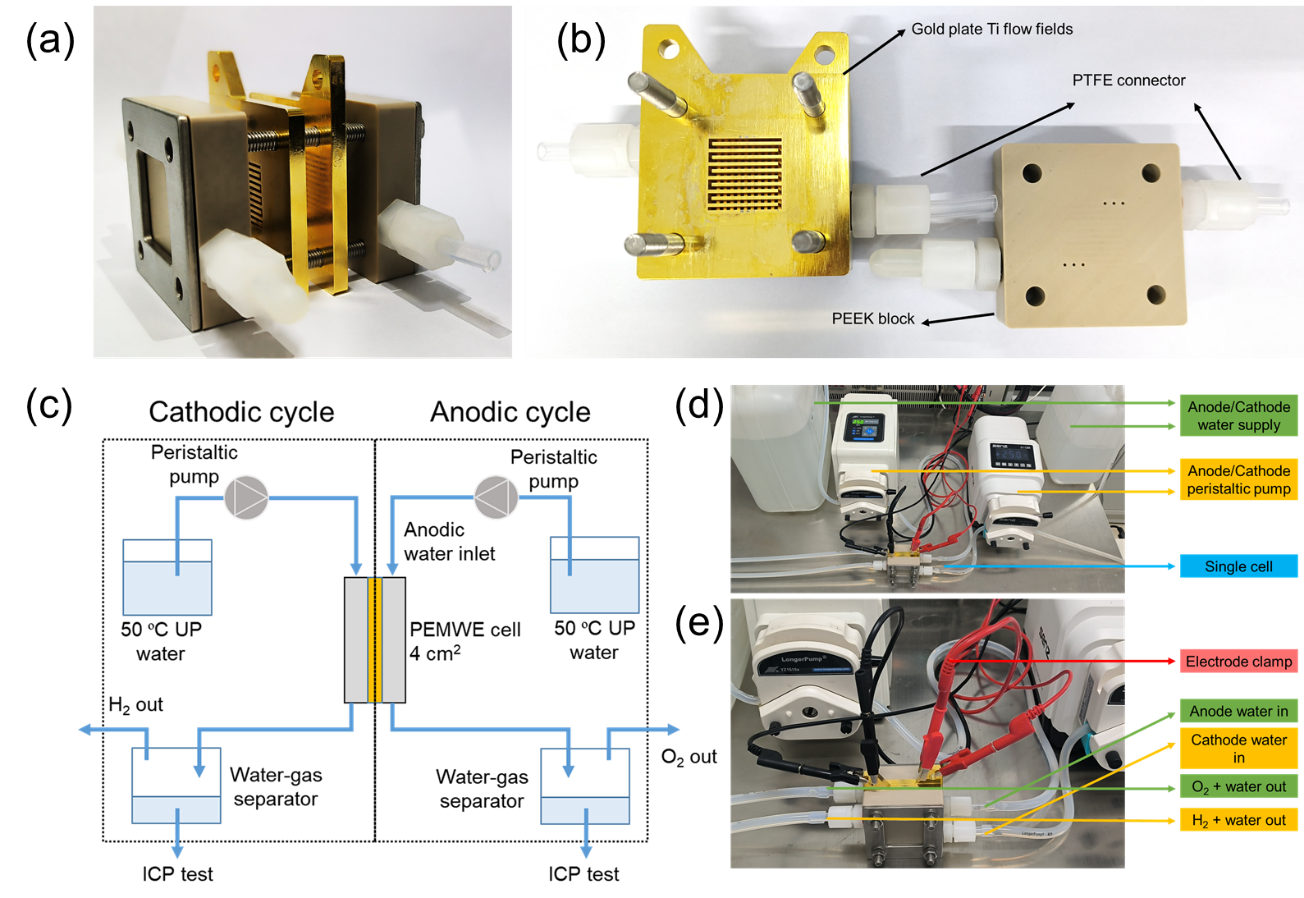


**Supplementary Fig. 80 |** **Precipitation free MEA setup for S-number determination.** (a, b) Optical photo of the component of a modified PEM electrolyzer; (c) Flow scheme of the precipitation free MEA setup, this scheme is originated from the previous work (Knoppel, J.; Mockl, M.; Escalera-Lopez, D.; Stojanovski, K.; Bierling, M.; Bohm, T.; Thiele, S.; Rzepka, M.; Cherevko, S., On the limitations in assessing stability of oxygen evolution catalysts using aqueous model electrochemical cells, *Nat. Commun.* 2021, 12, 2231)^16^ with modification; (d-e), photograph of the modified PEM electrolyzer employed during operation.


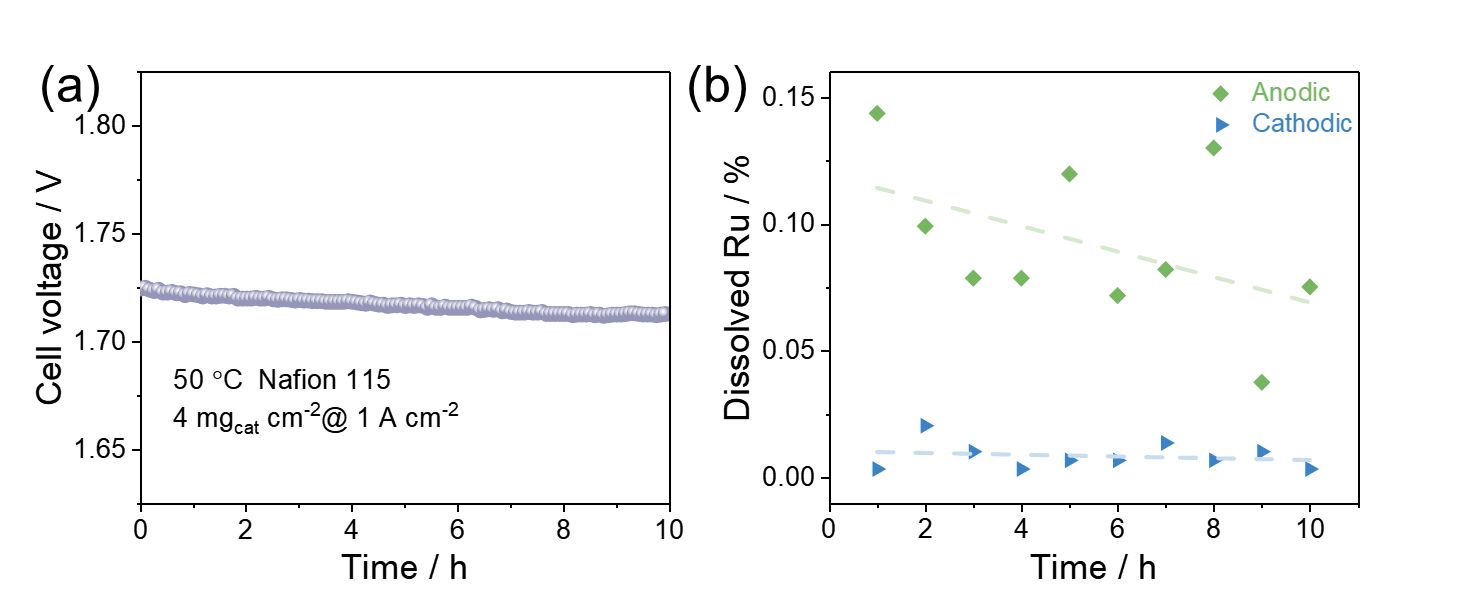


**Supplementary Fig. 81 | Stability of SnRuO_x_ in precipitation free MEA setup.** (a) Chronopotentiometry performance of SnRuO_x_ in PEM electrolyzer with Nafion 115 membrane; (b) the dissolved Ru content in the anode and cathode side.

**Supplementary Fig. 82 | Ru dissolution during CP test.** The total dissolution amount of Ru and the dissolution rate during the1300 h test. The metal dissolution amount are normalized to the total amount on the MEA.


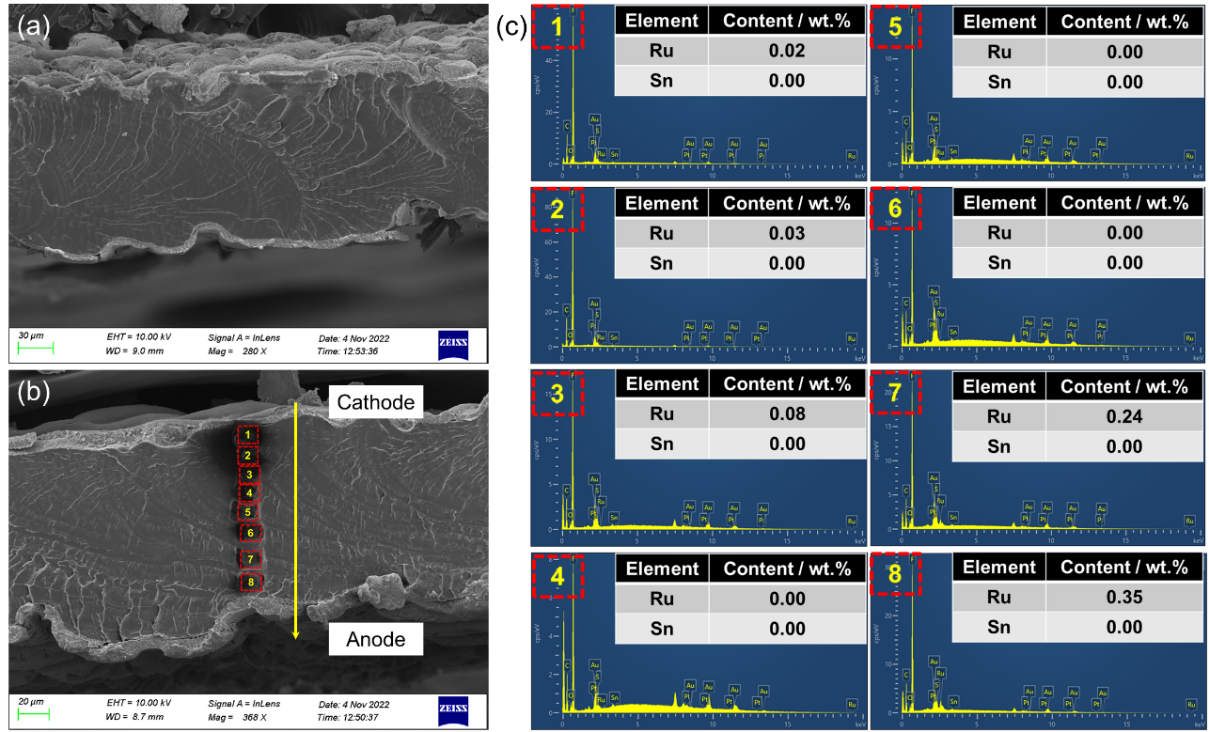


**Supplementary Fig. 83 | Structure of MEA after CP test.** (a, b) SEM images of the cross section of MEA after CP test. The red dotted box with serial number represents the position where EDS spectrum are collected, which is indexable to (c).


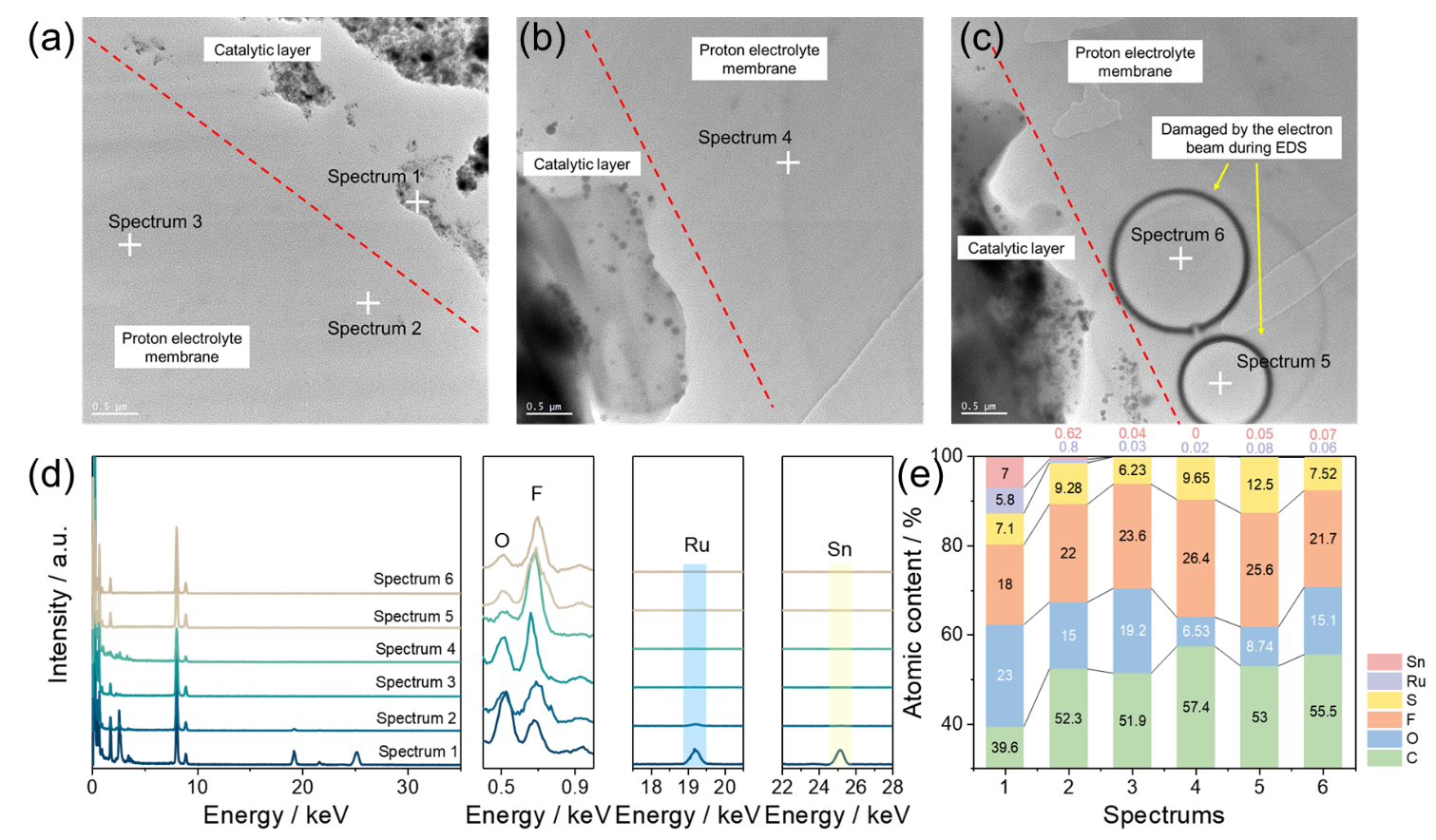


**Supplementary Fig. 84 |** (a-c) TEM images of the ultrathin section of membrane after CP test (nominal thickness of 100 nm); (d) EDS spectrums obtained at the position denoted in (a-c); (e) the atomic content of C, O, F, S, Ru, Sn in the EDS spectrums. The last 5 spectrums are averaged for Ru content calculation.

**Supplementary Table 1 |** Refined structural parameters for MRuO**_x_** from XRD.

| **Parameters** | **CeRuO_x_** | **SnRuO_x_** | **RuO_x_** | **CrRuO_x_** |
| --- | --- | --- | --- | --- |
| Space group | P42/MNM | P42/MNM | P42/MNM | P42/MNM |
| a / Å | 4.56230 | 4.54950 | 4.51130 | 4.50110 |
| b / Å | 4.56230 | 4.54950 | 4.51130 | 4.50110 |
| c / Å | 3.14270 | 3.12740 | 3.01980 | 3.00850 |
| α / º | 90.0000 | 90.0000 | 90.0000 | 90.0000 |
| β / º | 90.0000 | 90.0000 | 90.0000 | 90.0000 |
| γ / º | 90.0000 | 90.0000 | 90.0000 | 90.0000 |
| V / Å^3^ | 65.4140 | 64.7308 | 61.4584 | 60.9519 |
| *R_wp_* / % | 6.73 | 5.78 | 6.02 | 5.22 |
| *R_p_* / % | 5.01 | 4.55 | 4.88 | 3.91 |
| *x*^2^ | 1.948 | 1.629 | 1.812 | 1.573 |

**Supplementary Table 2 |** Concentration of M and Ru in MRuO**_x_** by ICP-OES and EDX measurement.

| **Method** | **ICP-OES** | | | | **EDX** | | | |
| --- | --- | --- | --- | --- | --- | --- | --- | --- |
|  | ppm | | mol L^-1^ | | wt. % | | at. % | |
|  | M | Ru | M | Ru | M | Ru | M | Ru |
| CeRuO**_x_** | 468.0 | 322.3 | 3.343 | 3.191 | 29.51 | 22.40 | 6.32 | 6.65 |
| SnRuO**_x_** | 133.8 | 119.6 | 1.127 | 1.184 | 33.48 | 27.88 | 9.23 | 9.04 |
| RuO**_x_** | - | 429.4 | - | 4.251 | - | 78.52 | - | 37.60 |
| CrRuO**_x_** | 157.7 | 291.5 | 3.033 | 2.886 | 12.57 | 26.26 | 5.77 | 5.73 |

**Supplementary Table 3 |** Fitting results of the O 1*s* XPS spectra for MRuO**_x._**

| **Samples** | **O_L_** | | **O_V_** | | **O_surf_** | | **O_adv_** | |
| --- | --- | --- | --- | --- | --- | --- | --- | --- |
|  | Position /eV | Area | Position /eV | Area | Position /eV | Area | Position /eV | Area |
| CeRuO**_x_** | 529.47 | 17995 | 531.30 | 7104 | 532.20 | 2383 | 533.23 | 599 |
| SnRuO**_x_** | 529.39 | 20601 | 531.30 | 5135 | 532.20 | 4648 | 533.52 | 3304 |
| RuO**_x_** | 529.25 | 13545 | 531.30 | 12799 | 532.20 | 8242 | 533.45 | 1115 |
| CrRuO**_x_** | 529.16 | 12098 | 531.30 | 22576 | 532.13 | 17632 | 533.50 | 2326 |

**Supplementary Table 4 |** Comparison on the OER activity with those previously reported catalysts.

| **Samples** | **η @10 mA cm^-2^ / mV** | **TOF @1.48 V / s^-1^** | **Tafel / mv dec^-1^** | **J_mass_ / A g_Ru_^-1^** | **References** |
| --- | --- | --- | --- | --- | --- |
| SnRuO**_x_** | 194 | 0.63 | 38.2 | 2360 | This work |
| RuO_2_ NS | 190 | - | 38.2 | - | ^14^ |
| a-PN-IN/C | 308 | 0.025 | 47.49 | 51.2 | ^17^ |
| FeCoNiIrRu HEA | 241 |  | 153 | 148 | ^18^ |
| PtCo-RuO_2_/C | 212 | 0.68 | 48.5 | 1619 | ^19^ |
| 9R-BaIrO_3_ | 230 | - | 80 | 99.56 | ^20^ |
| Ag_1_/IrO_x_ | 224 | - | 50.43 | 139 | ^21^ |
| Co_0.11_Ru_0.89_O_2_ | 169 | - | 49 | - | ^22^ |
| Ir-MoO_3_ | 156 | - | 48 | - | ^23^ |
| Ir/NiCo_2_O_4_ NSs | 240 | 0.33 | 60 | 1361 | ^24^ |
| Cex-IrO_2_ | 224 | 0.57 | 55.9 | 1146 | ^25^ |
| AD-HN-Ir | 216 | 3.69 | 39 | 7436 | ^26^ |
| Ru_1_Ir_1_Ox | 204 | 0.123 | 71.3 | 296 | ^27^ |
| RuIr-NC | 165 | - | 38.3 | - | ^28^ |
| 1T-IrO_2_ | 197 | - | 49 | 211 | ^29^ |
| Ru/MnO_2_ | 161 | - | 29.4 | - | ^30^ |
| Ir_0.06_Co_2.94_O_4_ | 294 | 0.125 |  | 251 | ^31^ |
| W_0.2_Er_0.1_Ru_0.7_O_2-δ_ | 168 | - | 66.8 | 1323 | ^32^ |
| GB-Ta_0.1_Tm_0.1_Ir_0.8_O_2-δ_ | 198 | - | 64 | 1953 | ^33^ |
| 3R-IrO_2_ | 188 | - | 52 | 418 | ^34^ |
| IrWB ally | 291 | - | 78 | 80 | ^35^ |
| Sr_1.7_Ru_5_Ir_1_O_13.7_ | 190 | - | 39 | - | ^8^ |
| S-300 | 188 | 0.0528 | 43.96 | - | ^36^ |

**Supplementary Table 5 |** Comparison on the OER activity with those previously reported.

| **Samples** | **Operation time / h** | **Degradation rate / μV h^-1^** | **References** |
| --- | --- | --- | --- |
| SnRuO**_x_** | 250@100 mA cm^-2^ | 107.2 | This work |
| RuO_2_ NS | 6@10 mA cm^-2^ | 6233 | ^14^ |
| a-PN-IN/C | 5@10 mA cm^-2^ | 11100 | ^17^ |
| S-300 | 8@10 mA cm^-2^ | 10375 | ^36^ |
| FeCoNiIrRu HEA | 14@1.516 V *vs.* RHE | - | ^18^ |
| PtCo-RuO_2_/C | 20@10 mA cm^-2^ | 4470 | ^19^ |
| 9R-BaIrO_3_ | 48@10 mA cm^-2^ | 1250 | ^20^ |
| Ag_1_/IrOx | 50@10 mA cm^-2^ | 760 | ^21^ |
| Co_0.11_Ru_0.89_O_2_ | 50@10 mA cm^-2^ | 1340 | ^22^ |
| Ir-MoO_3_ | 48@100 mA cm^-2^ | 2021 | ^23^ |
| Ir/NiCo_2_O_4_ NSs | 70@10 mA cm^-2^ | 743 | ^24^ |
| Cex-IrO_2_ | 100@10 mA cm^-2^ | 400 | ^25^ |
| AD-HN-Ir | 100@1.446 V *vs.* RHE | - | ^26^ |
| Ru_1_Ir_1_Ox | 110@100 mA cm^-2^ | 236 | ^27^ |
| RuIr-NC | 120@1 mA cm^-2^ | 333 | ^28^ |
| 1T-IrO_2_ | 126@250 mA cm^-2^ | 262 | ^29^ |
| Ru/MnO_2_ | 200@10 mA cm^-2^ | 845 | ^30^ |
| Ir_0.06_Co_2.94_O_4_ | 200@10 mA cm^-2^ | 435 | ^31^ |
| W_0.2_Er_0.1_Ru_0.7_O_2-δ_ | 500@10 mA cm^-2^ | 176 | ^32^ |
| GB-Ta_0.1_Tm_0.1_Ir_0.8_O_2-δ_ | 500@10 mA cm^-2^ | 6 | ^33^ |
| 3R-IrO_2_ | 511@10 mA cm^-2^ | 58.7 | ^34^ |
| IrWB ally | 800@100 mA cm^-2^ | 5 | ^35^ |
| Sr1.7Ru_5_Ir_1_O_13.7_ | 1500@10 mA cm^-2^ | 10 | ^8^ |

**Supplementary Table 6 |** Comparison on the S-number of SnRuO**_x_** (based on CA test in three-electrode set-up) and the previous reported Ir-based catalysts^37^.

| **Samples** | **S-number** |
| --- | --- |
| SnRuO**_x_** | 1.98×10^6^ |
| Ir_0.1_Ta_0.9_O_2.45_ NPs | 3.6×10^6^ |
| Ir_0.1_Ta_0.9_O_2.45_ film | 1.0×10^6^ |
| Rutile IrO_2_ (Alfa-Aesar) | 1.7×10^6^ |
| Rutile IrO_2_ (Sigma Aldrich) | 1.0×10^5^ |
| Hydrous IrOx | 6.6×10^4^ |
| Ir metal | 2.2×10^4^ |
| SrIrO_3_ film | 7.0×10^4^ |
| Ba_2_SrIrO_6_ | 2.2×10^4^ |
| SrCo_0.9_Ir_0.1_O_3-δ_ | 7.7×10^4^ |
| Sr_2_IrO_4_ | 1.3×10^5^ |

**Supplementary Table 7 |** *In situ* EXAFS fitting parameters at the Ru *K*-edge of SnRuO**_x_** and RuO**_x_**.

| Catalysts | Scattering path | CN | R(Å) | *R*-factor | σ^2^(10^-3^ Å^2^) |
| --- | --- | --- | --- | --- | --- |
| SnRuO**_x_** 1.0 V *vs.* RHE | Ru-O | 6.3 | 1.973 | 0.001 | 4.02 |
| SnRuO**_x_** 1.2 V *vs.* RHE | Ru-O | 6.1 | 1.973 | 0.002 | 3.55 |
| SnRuO**_x_** 1.4 V *vs.* RHE | Ru-O | 5.9 | 1.966 | 0.003 | 3.45 |
| SnRuO**_x_** 1.6 V *vs.* RHE | Ru-O | 6.0 | 1.972 | 0.002 | 3.81 |
| SnRuO**_x_** 1.8 V *vs.* RHE | Ru-O | 6.1 | 1.973 | 0.001 | 3.89 |
| SnRuO**_x_** 2.0 V *vs.* RHE | Ru-O | 5.5 | 1.955 | 0.006 | 2.90 |
| RuO**_x_** 1.0 V *vs.* RHE | Ru-O | 6.4 | 1.962 | 0.012 | 2.20 |
| RuO**_x_** 1.2 V *vs.* RHE | Ru-O | 5.4 | 1.948 | 0.005 | 3.56 |
| RuO**_x_** 1.4 V *vs.* RHE | Ru-O | 5.6 | 1.944 | 0.012 | 4.98 |
| RuO**_x_** 1.6 V *vs.* RHE | Ru-O | 5.2 | 1.928 | 0.017 | 1.06 |
| RuO**_x_** 1.8 V *vs.* RHE | Ru-O | 4.9 | 1.904 | 0.017 | 4.29 |
| RuO**_x_** 2.0 V *vs.* RHE | Ru-O | 4.7 | 1.865 | 0.013 | 0.99 |

CN, coordination number; R, the distance between absorber and backscatter atoms; σ^2^, Debye-Waller factor to account for both thermal and structural disorders; R-factor indicates the goodness of the fit.

**Supplementary Table 8 |** Comparison on the PEM electrolyzer performance and anode noble metal cost with those previously reported.

| **Samples** | **Anode Noble metal cost / US $ cm^-2^** | **Cell voltage / V** | | **Energy efficiency / %** | | **Energy consumption / kWh kg^-1^H**_2_ | | **References** |
| --- | --- | --- | --- | --- | --- | --- | --- | --- |
|  |  | @1 A cm^-2^ | @2 A cm^-2^ | @1 A cm^-2^ | @2 A cm^-2^ | @1 A cm^-2^ | @2 A cm^-2^ |  |
| SnRuO**_x_** | 0.0194 | 1.565 | 1.655 | 78.7 | 74.3 | 41.9 | 44.4 | This work |
| 30Ir/Au/CP | 0.0238 | 1.652 | 1.756 | 74.5 | 70.0 | 44.3 | 47.1 | ^38^ |
| IrOx hybride | 0.0262 | 1.602 | 1.689 | 76.8 | 72.8 | 42.9 | 45.3 | ^39^ |
| Y_1.85_Ba_0.15_Ru_2_O_7_ | 0.0288 | 1.638 | 1.824 | 75.1 | 67.4 | 43.9 | 48.9 | ^40^ |
| Y_1.75_Ca_0.25_Ru_2_O_7_ | 0.0313 | 1.663 | 1.805 | 74.0 | 68.1 | 44.6 | 48.4 | ^41^ |
| e-IrO_2_/Ti | 0.0394 | 1.606 | 1.869 | 76.6 | 65.8 | 43.0 | 50.1 | ^42^ |
| Heraeus | 0.0446 | 1.613 | 1.756 | 76.3 | 70.0 | 43.2 | 47.1 | ^43^ |
| IrO_2_@TiO_2_ | 0.0525 | 1.675 | 1.775 | 73.4 | 69.3 | 44.9 | 47.6 | ^44^ |
| RuOx | 0.0551 | 1.733 | 1.883 | 71.0 | 65.3 | 46.4 | 50.5 | This work |

**Supplementary Note 11 | Calculation of the anode noble metal cost, energy efficiency and energy consumption at 1 A cm^-2^ and 2 A cm^-2^.**

Considering the fact that the cost of noble metal in the anode accounts for a high proportion in the MEA cost (~40 %), cost accounting of the anode noble metal is thus very necessary. The price of the noble metal (Pt, Ir, Au, Ru) is obtained from the *Johnson Matthey Price Charts*.

The energy efficiency of a PEM electrolyzer can be calculated by the following equation:

Energy efficiency = $\frac{1.23 V}{U_{cell}}$ equation (1)

where 1.23 V represent the theoretical energy of the products, $U_{cell}$ is the cell voltage (V) required to deliver a current density of 1 A cm^-2^ or 2 A cm^-2^.

The energy consumption of a PEM electrolyzer are calculated by the following equation:

Energy consumption = $\frac{U_{cell}I_{cell}t}{m_{H_{2}}}$ equation (2)

where $I_{cell}$ is the current delivered (A), t is the operation time (h), $m_{H_{2}}$ is the mass of hydrogen produced in a *t* duration, which can be calculated by the Faraday's laws of electrolysis:

$m_{H_{2}}=\frac{I_{cell}\times t}{z\times F}\times M_{H_{2}}$ equation (3)

z in equation (3) is the number of electrons transferred to produce one hydrogen molecule (2), $M_{H_{2}}$relative molecular mass (2 g mol^-1^).

**Supplementary Table 9 |** Comparison on the PEM electrolyzer stability and degradation rate with those previously reported.

| **Samples** | **Operation time / h** | **Degradation rate / μV h^-1^** | **References** |
| --- | --- | --- | --- |
| Ir/CP | 12@1 A cm^-2^ | 14576 | ^45^ |
| Y_1.85_Ba_0.15_Ru_2_O_7_ | 14.3@0.2 A cm^-2^ | 40601 | ^40^ |
| Y_1.75_Ba_0.25_Ru_2_O_7_ | 16.7@0.15 A cm^-2^ | 9108 | ^41^ |
| 30Ir/Au/CP | 20@1 A cm^-2^ | 2407 | ^38^ |
| PtCo-RuO_2_/C | 24@1 A cm^-2^ | 546 | ^19^ |
| Ir/B_4_C | 48@1 A cm^-2^ | 2288 | ^46^ |
| RuOx | 50@1 A cm^-2^ | 3843 | This work |
| IrSn | 100@1 A cm^-2^ | 480 | ^47^ |
| Sr_1.7_Ru_5_Ir_1_O_13.7_ | 150@1 A cm^-2^ | 553 | ^8^ |
| F108-Ir_0.6_Sn_0.4_O_2_ | 200@0.5 A cm^-2^ | 135 | ^48^ |
| GB-Ta_0.1_Tm_0.1_Ir_0.8_O_2-δ_ | 500@1.5 A cm^-2^ | 160 | ^33^ |
| Ir_0.7_Ru_0.3_O_2_ | 400@1 A cm^-2^ | 43 | ^49^ |
| RuO_2_ | 300@2 A cm^-2^ | 450 | ^50^ |
| RuO_2_/SnO_2_ | 235@0.25 A cm^-2^ | - | ^51^ |
| Ru_0.8_Pd_0.2_O_2_ | 100@1 A cm^-2^ | - | ^52^ |
| RuO_2_ | 2000@1.1 A cm^-2^ | - | ^53^ |
| Ni-RuO_2_ | 1500@0.2 A cm^-2^ | - | ^7^ |
| SnRuO**_x_** | 1300@1 A cm^-2^ | 53 | This work |

**Supplementary Note 13 | Calculation of the S-number and lifetime of SnRuO_x_ in a PEM electrolyzer.**

The S-number of SnRuO**_x_** was calculated based on the following equation:

S-number = $\frac{n_{O_{2}}}{n_{Ru}}$ equation (4)

where $n_{O_{2}}$ is the amount of oxygen molecules produced during a 10 h test at 1 A cm^-2^, according to the Faraday's laws of electrolysis:

$n_{O_{2}}=\frac{j\times s\times t}{z\times F}$ equation (5)

where j is the current density (1 A cm^-2^), s is the geometry area of the electrode (2×2 cm^-2^), t is the electrolysis time (10×3600 s), z is the is the number of electrons transferred to produce an oxygen molecule (4), F is the Faraday constant (96484 C mol^-1^).

$n_{Ru}$ in equation (4) is the total amount of Ru dissolved in the electrolyte. The dissolved Ru amount is determined to be 36.225 μg (see Supplementary Note 12 for detail). The stability number of SnRuO**_x_** is calculated to be 1.04×10^6^.

The lifetime of SnRuO**_x_** in a PEM electrolyzer are calculated based on the previous reports:

Lifetime = $\frac{S\times z\times F\times m}{j\times M}$ equation (6)

where S is the stability number of SnRuO**_x_** obtained in a PEM electrolyzer, z is the number of electrons per evolved O_2_, F is the Faraday constant (96484 C mol^-1^), m is the loading of the Ru on the electrode (g cm^-2^), j is the applied current density (A cm^-2^), and M is the molar mass of Ru (101.07 g mol^-1^). Here, the lifetime of SnRuO**_x_** is estimated based on a SnRuO**_x_** loading of 2 mg_Ru_ cm^-2^ and operating current density of 1 A cm^-2^.

**Supplementary Table 10 |** Atomic ratio and mass content of different element in the EDS spectrum (averaged by the last 5 spectrums show in Supplementary Fig. 84d).

| **Element** | **at.%** | **wt.%** |
| --- | --- | --- |
| C | 54.28 | 40.8601 |
| O | 11.925 | 11.96897 |
| F | 24.6 | 29.32021 |
| S | 8.585 | 17.23331 |
| Ru | 0.045 | 0.28511 |
| Sn | 0.045 | 0.33507 |

**Supplementary References**

1. Man IC*, et al.* Universality in Oxygen Evolution Electrocatalysis on Oxide Surfaces. *ChemCatChem* **3**, 1159 – 1165 (2011).

2. Lou Y*, et al.* Ultralow-temperature CO oxidation on an In2O3–Co3O4 catalyst: a strategy to tune CO adsorption strength and oxygen activation simultaneously. *Chem. Commun.* **50**, 6835-6838 (2014).

3. Klyukin K*, et al.* Role of Dissolution Intermediates in Promoting Oxygen Evolution Reaction at RuO2(110) Surface. *J. Phys. Chem. C* **123**, 22151-22157 (2019).

4. Zheng Y-R*, et al.* Monitoring oxygen production on mass-selected iridium–tantalum oxide electrocatalysts. *Nat. Energy*, 10.1038/s41560-021-00948-w, (2021).

5. Harzandi AM*, et al.* Ruthenium Core–Shell Engineering with Nickel Single Atoms for Selective Oxygen Evolution via Nondestructive Mechanism. *Adv. Energy Mater.* **11**, 2003448 (2021).

6. Scott SB*, et al.* The low overpotential regime of acidic water oxidation part II: trends in metal and oxygen stability numbers. *Energy Environ Sci* **15**, 1988-2001 (2022).

7. Wu ZY*, et al.* Non-iridium-based electrocatalyst for durable acidic oxygen evolution reaction in proton exchange membrane water electrolysis. *Nat. Mater.*, 10.1038/s41563-022-01380-5, (2022).

8. Wen Y*, et al.* Stabilizing Highly Active Ru Sites by Suppressing Lattice Oxygen Participation in Acidic Water Oxidation. *J. Am. Chem. Soc.* **143**, 6482-6490 (2021).

9. Chen J*, et al.* Energetic Span as a Rate-Determining Term for Electrocatalytic Volcanos. *ACS Catal.* **8**, 10590-10598 (2018).

10. Exner KS*, et al.* A Universal Approach To Determine the Free Energy Diagram of an Electrocatalytic Reaction. *ACS Catal.* **8**, 1864-1879 (2018).

11. Exner KS. Why approximating electrocatalytic activity by a single free‐energy change is insufficient. *Electrochim. Acta* **375**, 137975 (2021).

12. Dam AP*, et al.* On the role of microkinetic network structure in the interplay between oxygen evolution reaction and catalyst dissolution. *Sci. Rep.* **10**, 14140 (2020).

13. Shinagawa T*, et al.* Insight on Tafel slopes from a microkinetic analysis of aqueous electrocatalysis for energy conversion. *Sci. Rep.* **5**, 13801 (2015).

14. Zhao ZL*, et al.* Boosting Oxygen Evolution Reaction Using Defect-rich Ultra-Thin Ruthenium Oxide Nanosheets in Acidic Media. *Energy Environ. Sci.* **13**, 5143-5151 (2020).

15. Fang W-C*, et al.* Effect of temperature annealing on capacitive and structural properties of hydrous ruthenium oxides. *J. Power Sources* **160**, 1506-1510 (2006).

16. Knoppel J*, et al.* On the limitations in assessing stability of oxygen evolution catalysts using aqueous model electrochemical cells. *Nat. Commun.* **12**, 2231 (2021).

17. Choi S*, et al.* Pt Dopant: Controlling the Ir Oxidation States toward Efficient and Durable Oxygen Evolution Reaction in Acidic Media. *Adv. Funct. Mater.* **30**, 2003935 (2020).

18. Zhu H*, et al.* High-entropy alloy stabilized active Ir for highly efficient acidic oxygen evolution. *Chem. Eng. J.* **431**, 133251 (2021).

19. Jin H*, et al.* Safeguarding RuO_2_ phase against lattice oxygen oxidation during acidic water electrooxidation. *Energy Environ. Sci.* **15**, 1119-1130 (2021).

20. Li N*, et al.* Identification of the Active-Layer Structures for Acidic Oxygen Evolution from 9R-BaIrO_3_ Electrocatalyst with Enhanced Iridium Mass Activity. *J. Am. Chem. Soc.* **143**, 18001-18009 (2021).

21. Zhang F-F*, et al.* Iridium Oxide Modified with Silver Single Atom for Boosting Oxygen Evolution Reaction in Acidic Media. *ACS Energy Lett.* **6**, 1588-1595 (2021).

22. Tian Y*, et al.* A Co-doped nanorod-like RuO_2_ electrocatalyst with abundant oxygen vacancies for acidic water oxidation. *iScience* **23**, 100756 (2019).

23. Liu X*, et al.* Restructuring highly electron-deficient metal-metal oxides for boosting stability in acidic oxygen evolution reaction. *Nat. Commun.* **12**, 5676 (2021).

24. Yin J*, et al.* Iridium Single Atoms Coupling with Oxygen Vacancies Boosts Oxygen Evolution Reaction in Acid Media. *J. Am. Chem. Soc.* **142**, 18378-18386 (2020).

25. Wang Y*, et al.* Ce-Doped IrO_2_ Electrocatalysts with Enhanced Performance for Water Oxidation in Acidic Media. *ACS Appl. Mater. Interfaces* **12**, 37006-37012 (2020).

26. Su H*, et al.* In-situ spectroscopic observation of dynamic-coupling oxygen on atomically dispersed iridium electrocatalyst for acidic water oxidation. *Nat. Commun.* **12**, 6118 (2021).

27. He J*, et al.* Regulating Electron Redistribution of Intermetallic Iridium Oxide by Incorporating Ru for Efficient Acidic Water Oxidation. *Adv. Energy Mater.* **11**, 2102883 (2021).

28. Wu D*, et al.* Efficient overall water splitting in acid with anisotropic metal nanosheets. *Nat. Commun.* **12**, 1145 (2021).

29. Dang Q*, et al.* Iridium metallene oxide for acidic oxygen evolution catalysis. *Nat. Commun.* **12**, 6007 (2021).

30. Lin C*, et al.* In-situ reconstructed Ru atom array on α-MnO_2_ with enhanced performance for acidic water oxidation. *Nat. Catal.* **4**, 1012-1023 (2021).

31. Shan J*, et al.* Short-Range Ordered Iridium Single Atoms Integrated into Cobalt Oxide Spinel Structure for Highly Efficient Electrocatalytic Water Oxidation. *J. Am. Chem. Soc.* **143**, 5201-5211 (2021).

32. Hao S*, et al.* Dopants fixation of Ruthenium for boosting acidic oxygen evolution stability and activity. *Nat. Commun.* **11**, 5368 (2020).

33. Hao S*, et al.* Torsion strained iridium oxide for efficient acidic water oxidation in proton exchange membrane electrolyzers. *Nat. Nanotechnol.* **16**, 1371-1377 (2021).

34. Fan Z*, et al.* Extraordinary acidic oxygen evolution on new phase 3R-iridium oxide. *Joule* **5**, 3221-3234 (2021).

35. Li R*, et al.* IrW nanochannel support enabling ultrastable electrocatalytic oxygen evolution at 2 A cm^-2^ in acidic media. *Nat. Commun.* **12**, 3540 (2021).

36. Su J*, et al.* Assembling Ultrasmall Copper-Doped Ruthenium Oxide Nanocrystals into Hollow Porous Polyhedra: Highly Robust Electrocatalysts for Oxygen Evolution in Acidic Media. *Adv. Mater.* **30**, 1801351 (2018).

37. Zheng Y-R*, et al.* Monitoring oxygen production on mass-selected iridium–tantalum oxide electrocatalysts. *Nat. Energy* **7**, 55–64 (2021).

38. Kim H*, et al.* Dendritic gold-supported iridium/iridium oxide ultra-low loading electrodes for high-performance proton exchange membrane water electrolyzer. *Appl. Catal. B* **283**, 119596 (2021).

39. Hegge F*, et al.* Efficient and Stable Low Iridium Loaded Anodes for PEM Water Electrolysis Made Possible by Nanofiber Interlayers. *ACS Appl. Energy Mater.* **3**, 8276-8284 (2020).

40. Feng Q*, et al.* Influence of Surface Oxygen Vacancies and Ruthenium Valence State on the Catalysis of Pyrochlore Oxides. *ACS Appl. Mater. Interfaces* **12**, 4520-4530 (2020).

41. Feng Q*, et al.* Oxygen vacancy engineering of yttrium ruthenate pyrochlores as an efficient oxygen catalyst for both proton exchange membrane water electrolyzers and rechargeable zinc-air batteries. *Appl. Catal. B* **260**, 118176 (2020).

42. Lee B-S*, et al.* Development of electrodeposited IrO2 electrodes as anodes in polymer electrolyte membrane water electrolysis. *Appl. Catal. B* **179**, 285-291 (2015).

43. Böhm D*, et al.* Highly conductive titania supported iridium oxide nanoparticles with low overall iridium density as OER catalyst for large-scale PEM electrolysis. *Appl. Mater. Today* **24**, 101134 (2021).

44. Pham CV*, et al.* IrO_2_ coated TiO_2_ core-shell microparticles advance performance of low loading proton exchange membrane water electrolyzers. *Appl. Catal. B* **269**, 118762 (2020).

45. Oh JH*, et al.* Activity and stability of Ir-based gas diffusion electrode for proton exchange membrane water electrolyzer. *Chem. Eng. J.* **420**, 127696 (2021).

46. Islam J*, et al.* Enhancing the activity and durability of iridium electrocatalyst supported on boron carbide by tuning the chemical state of iridium for oxygen evolution reaction. *J. Power Sources* **512**, 230506 (2021).

47. Li G*, et al.* Iridium-Tin oxide solid-solution nanocatalysts with enhanced activity and stability for oxygen evolution. *J. Power Sources* **325**, 15-24 (2016).

48. Jiang G*, et al.* An effective oxygen electrode based on Ir_0.6_Sn_0.4_O_2_ for PEM water electrolyzers. *J. Energy Chem.* **39**, 23-28 (2019).

49. Wang L*, et al.* Highly active anode electrocatalysts derived from electrochemical leaching of Ru from metallic Ir_0.7_Ru_0.3_ for proton exchange membrane electrolyzers. *Nano Energy* **34**, 385-391 (2017).

50. Audichon T*, et al.* Elaboration and characterization of ruthenium nano-oxides for the oxygen evolution reaction in a Proton Exchange Membrane Water Electrolyzer supplied by a solar profile. *Electrochim. Acta* **132**, 284-291 (2014).

51. Lim JY*, et al.* Highly stable RuO2/SnO2nanocomposites as anode electrocatalysts in a PEM water electrolysis cell. *International Journal of Energy Research* **38**, 875-883 (2014).

52. Shiva Kumar S*, et al.* Preparation of RuxPd1-xO2 electrocatalysts for the oxygen evolution reaction (OER) in PEM water electrolysis. *Ionics* **24**, 2411-2419 (2017).

53. Ma H*, et al.* Study of ruthenium oxide catalyst for electrocatalytic performance in oxygen evolution. *J. Mol. Catal. A: Chem.* **247**, 7-13 (2006).
